# Supplementary figures and images for: Fine-scale genomic analyses of admixed individuals reveal unrecognized genetic ancestry components in Argentina
Source: PLoS One. 2020 Jul 16;15(7):e0233808. doi: 10.1371/journal.pone.0233808 (PMC7365470; doi:10.1371/journal.pone.0233808)

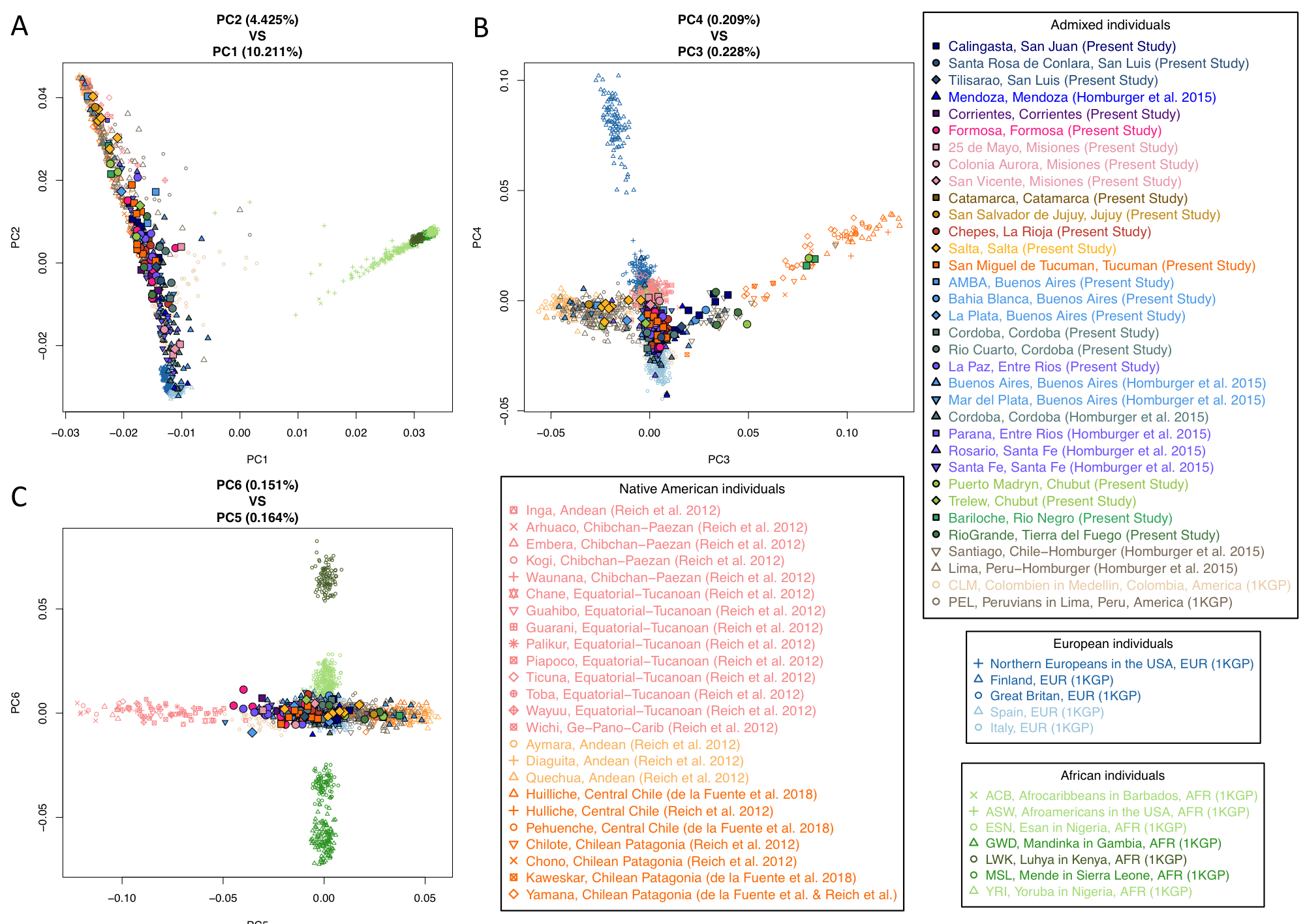

Supplement: S1 Fig — A: PC2 vs PC1; B: PC4 vs PC3; C: PC6 vs PC5. The percentage of variance explained by each principal component (PC) is given. Each point represents an individual following the color and point codes given in legend. (TIF) [file pone.0233808.s001.tif]

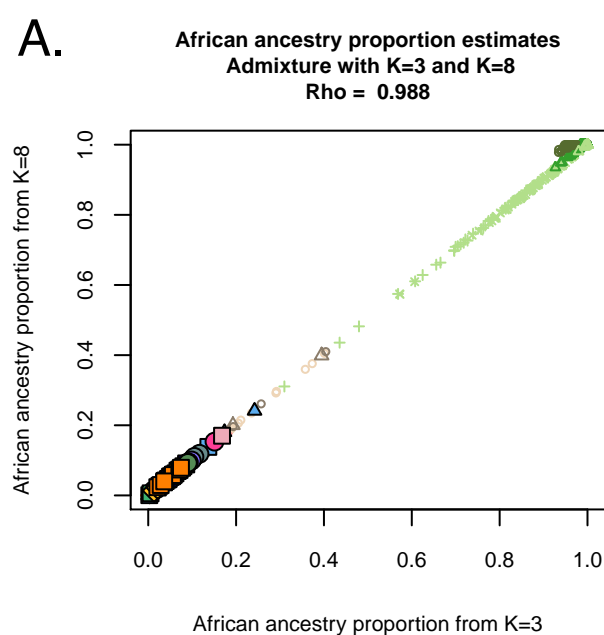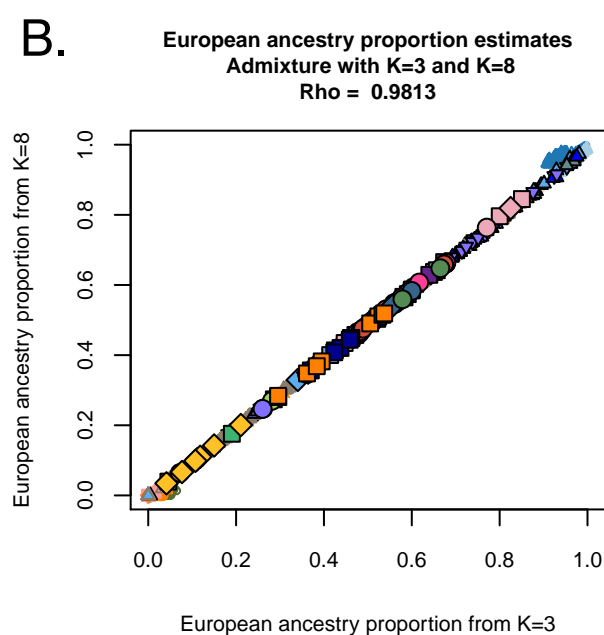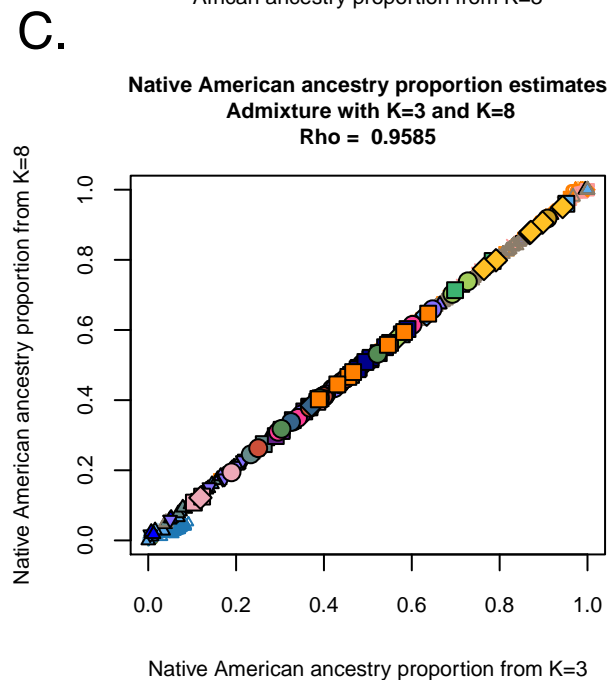

Supplement: S3 Fig — Comparison of the African, European and Native American ancestry proportion estimates obtained with Admixture models with K = 3 and K = 8 applied to DS1. (A) African ancestry proportions for K = 3 are as observed in green in S2B Fig, while for K = 8 they are estimated as the sum of the three greenish colors observed in Main Fig 2. (B) European ancestry proportions for K = 3 are as observed in blue in S2B Fig, while for K = 8 they are estimated as the sum of the two bluish colors observed in Main Fig 2. (C) Native American ancestry proportions for K = 3 are as observed in orange in S2B Fig, while for K = 8 they are estimated as the sum of the three reddish colors observed in Main Fig 2. (PDF) [file pone.0233808.s003.pdf]

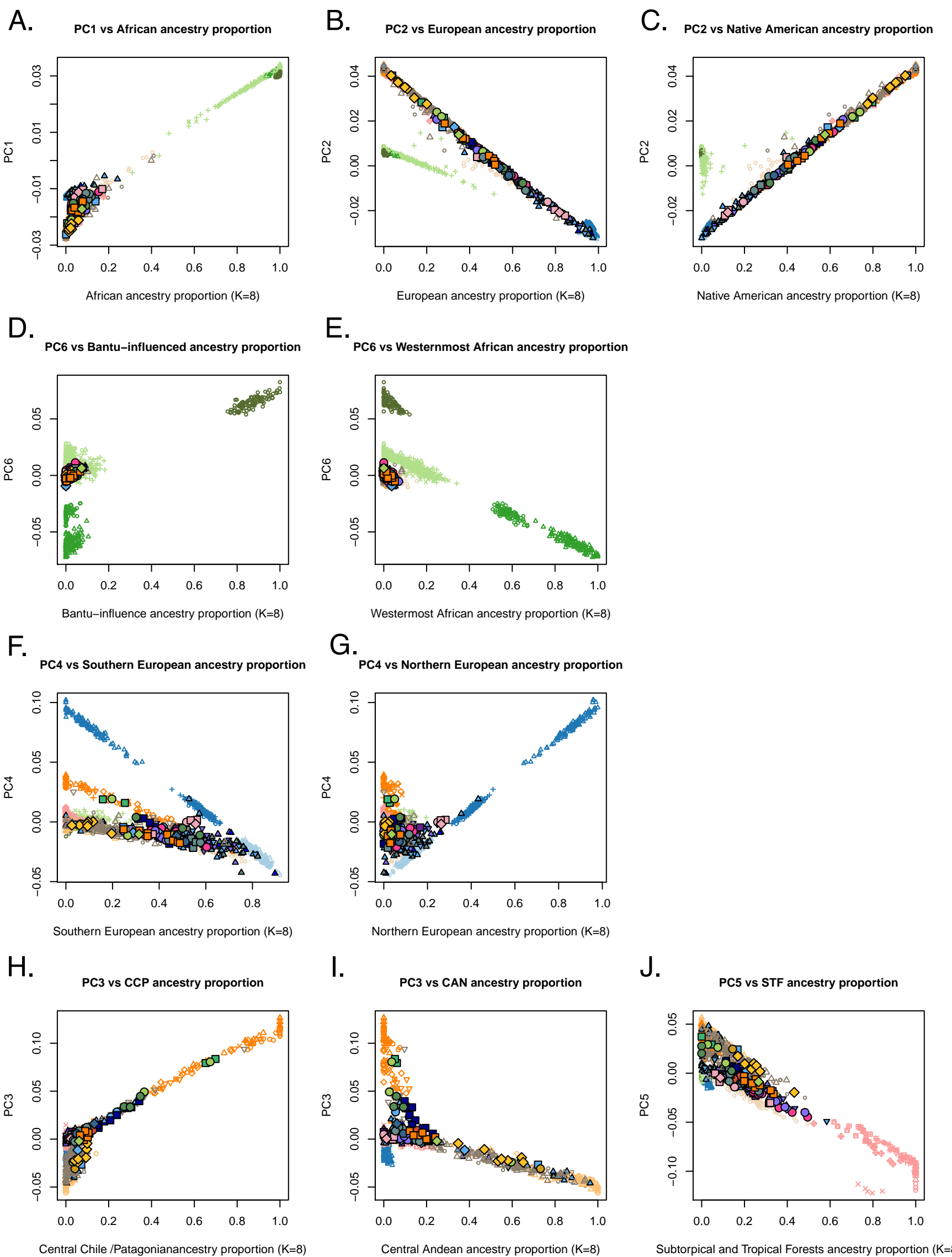

Supplement: S4 Fig — Comparison of ancestry proportion estimates from Admixture model with K = 8 and the 6 first Principal Components (PCs) in DS1. (A) PC1 vs African ancestry proportions (estimated as the sum of the three greenish colors observed in Main Main Fig 2). (B) PC2 vs European ancestry proportions (estimated as the sum of the two bluish colors observed in Main Fig 2). (C) PC2 vs Native American ancestry proportions (estimated as the sum of the three redish colors observed in Main Fig 2). (D) PC6 vs Bantu-influenced ancestry proportions (dark olive green in Main Fig 2). (E) PC6 vs Western African ancestry proportions (dark green in Main Fig 2). (F) PC4 vs Southern European ancestry proportions (light blue in Main Fig 2). (G) PC4 vs Northern European ancestry proportions (dark blue in Main Fig 2). (H) PC3 vs Cenral Chile / Patagonia ancestry proportions (orange in Main Fig 2). (I) PC3 vs Central Andes ancestry proportions (yellow in Main Fig 2). (J) PC5 vs Subtropical and Tropical Forests ancestry proportions (pink in Main Fig 2). (PDF) [file pone.0233808.s004.pdf]

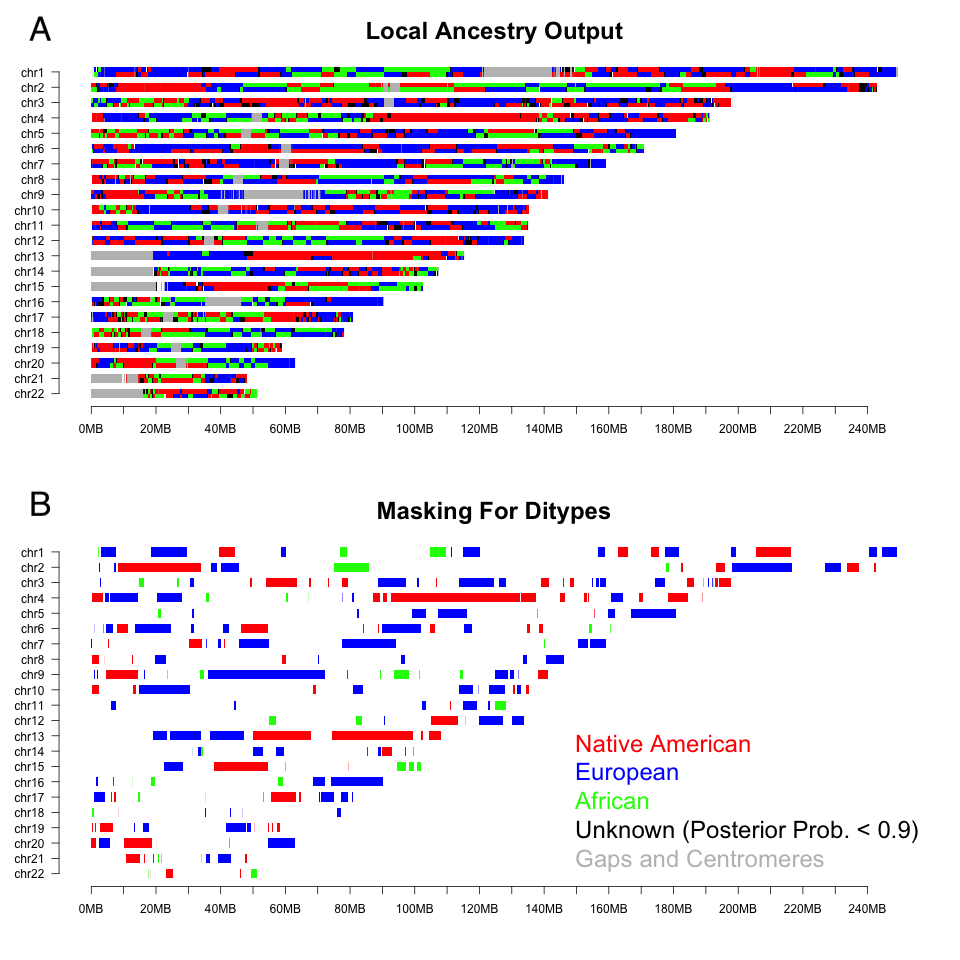

Supplement: S5 Fig — (A) RFMIX output for a given admixed individual. (B) Masked genotype showing ditypes of Native American (red), European (blue) and African (green) ancestry. Gaps are represented in grey and regions with unassigned ancestry (Unknown) are in black. (TIFF) [file pone.0233808.s005.tiff]

# Choose Number of PCs ASPCA in Europe

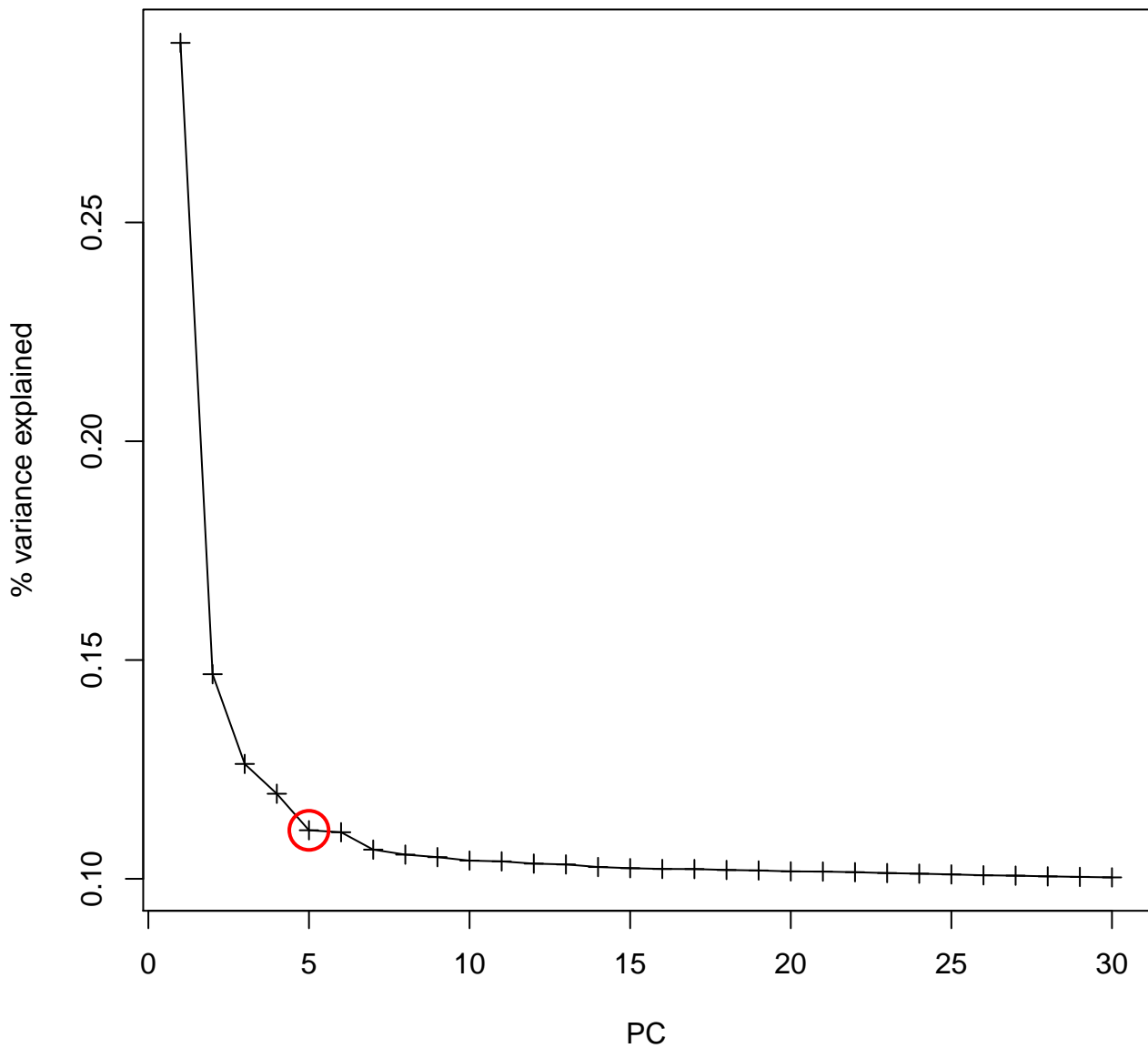

Supplement: S6 Fig — Elbow method to determine which PC minimizes the angle of the curve from the chart “Percentage of variance explained versus Number of PCs” (PDF) [file pone.0233808.s006.pdf]

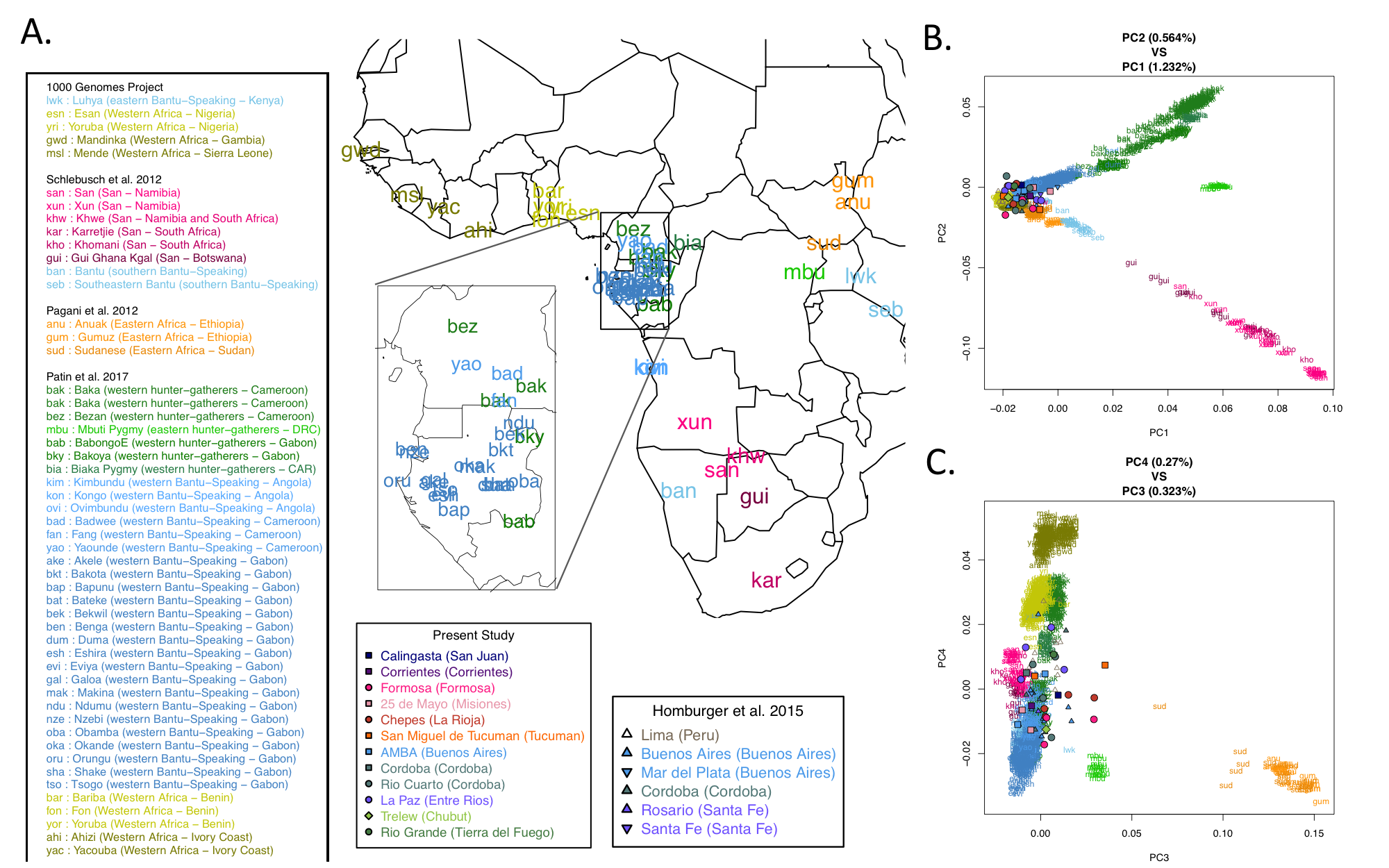

Supplement: S9 Fig — (A) Localization map of the 1685 reference samples with >99% of African ancestry. (B-C) Principal Components performed using the African reference samples (represented as in panel A), and South American samples masked for African ancestry. (TIF) [file pone.0233808.s009.tif]

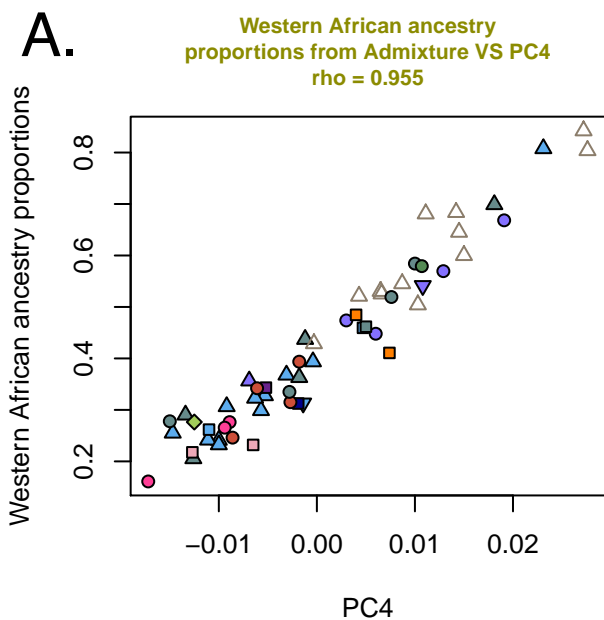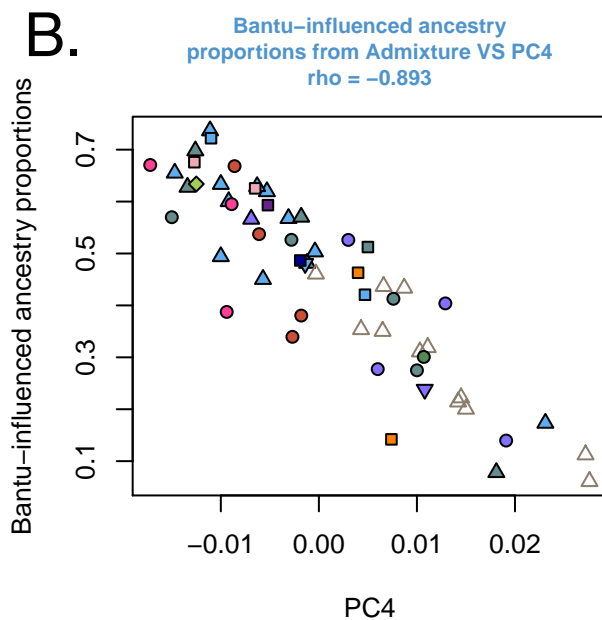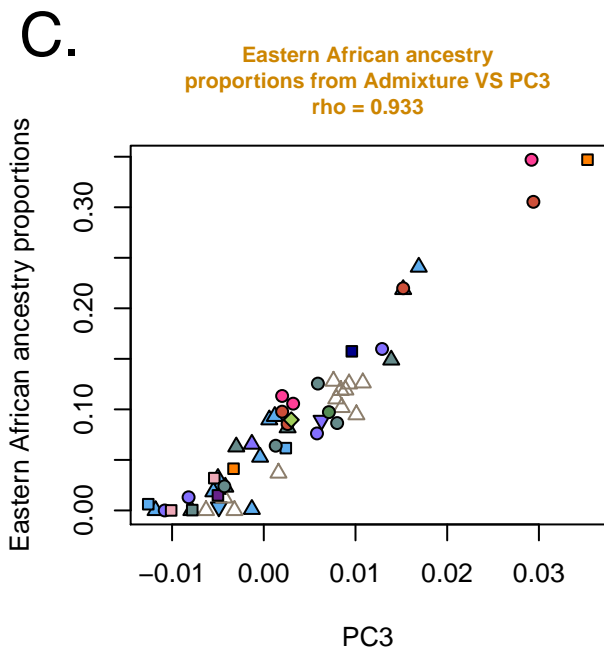

Supplement: S11 Fig — Comparison of ancestry proportion estimates from Admixture model with K = 5 and some Principal Components (PCs) in admixed samples from DS5. (A) PC3 vs Western African ancestry proportions (yellow in S10 Fig). (B) PC3 vs Bantu-influenced ancestry proportions (blue in S10 Fig). (C) PC4 vs Eastern African ancestry proportions (orange in S10 Fig). (PDF) [file pone.0233808.s011.pdf]

# Choose Number of PCs ASPCA in South America

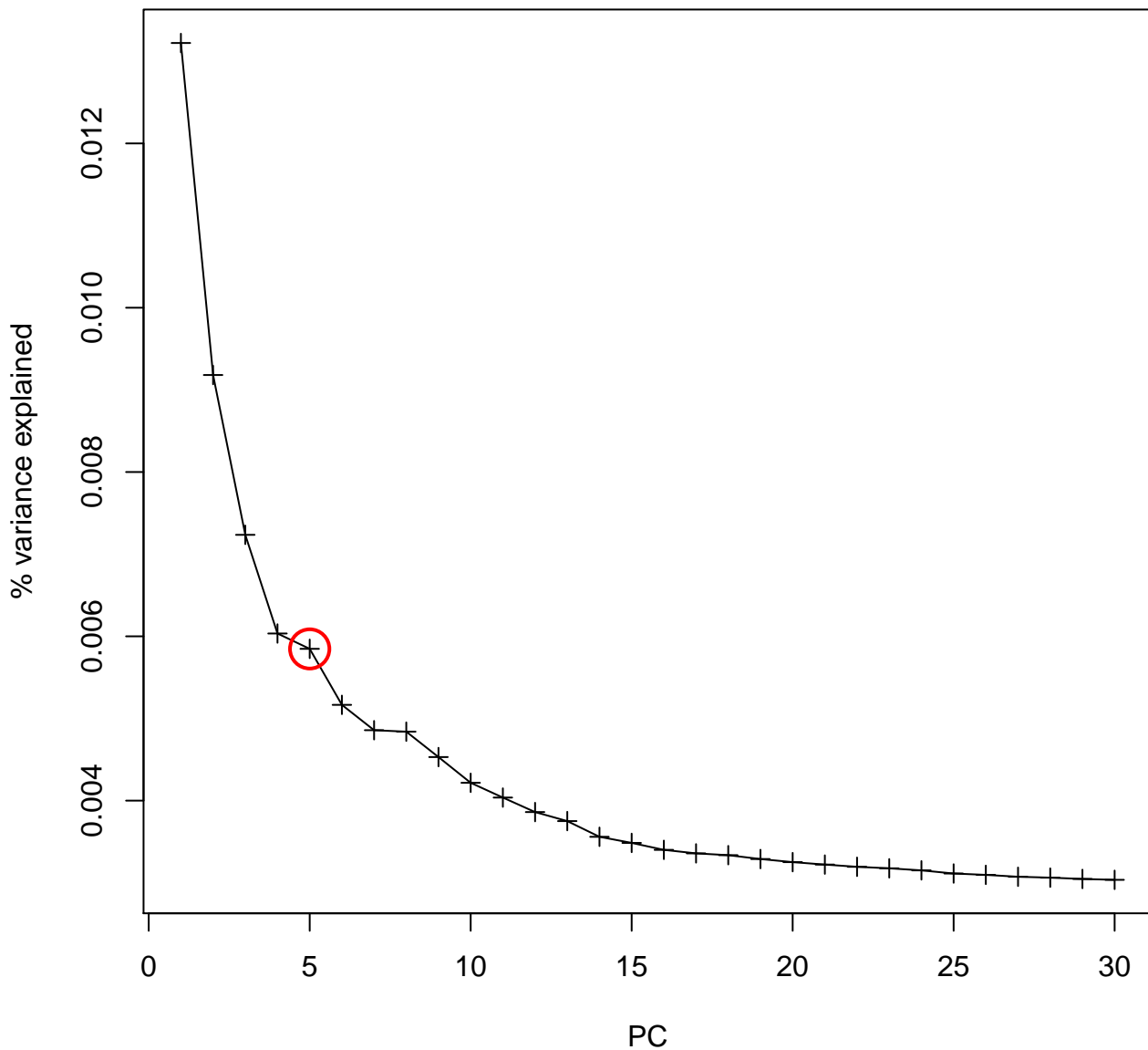

Supplement: S12 Fig — Elbow method to determine which PC minimizes the angle of the curve from the chart “Percentage of variance explained versus Number of PCs” (PDF) [file pone.0233808.s012.pdf]

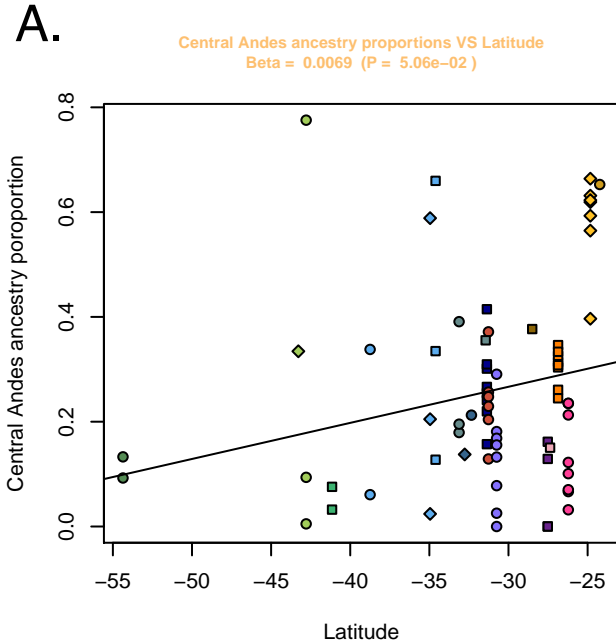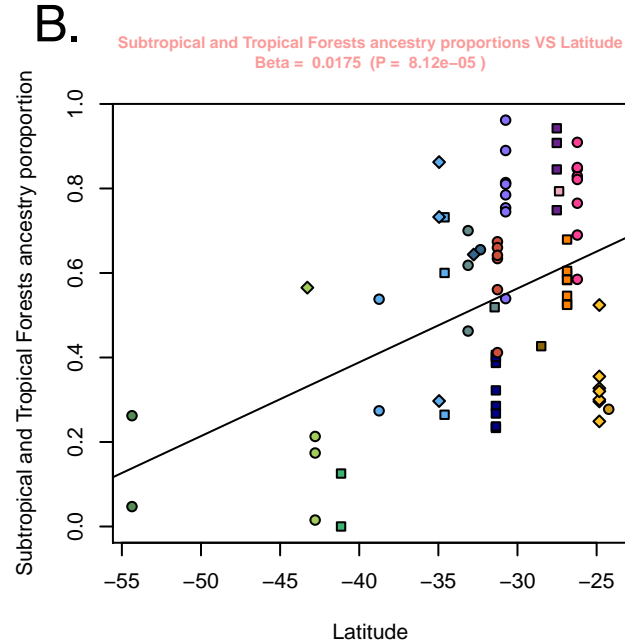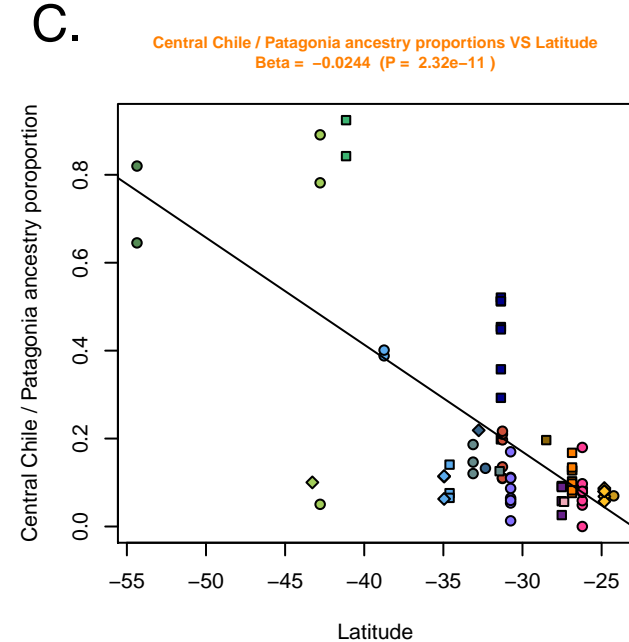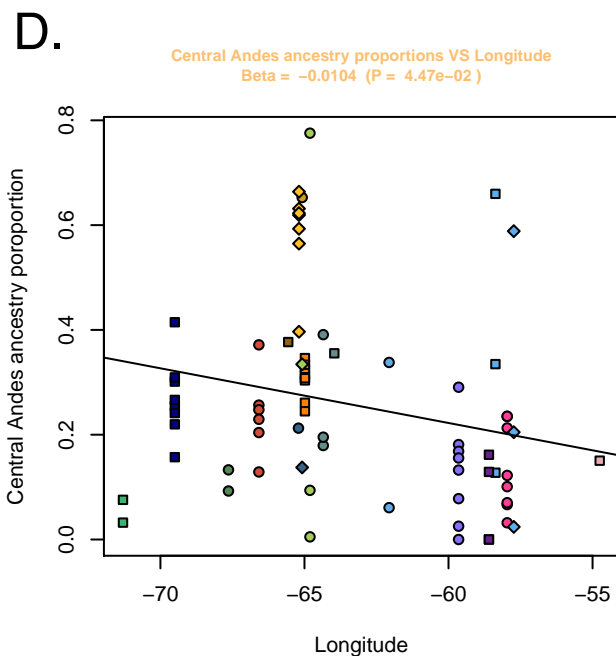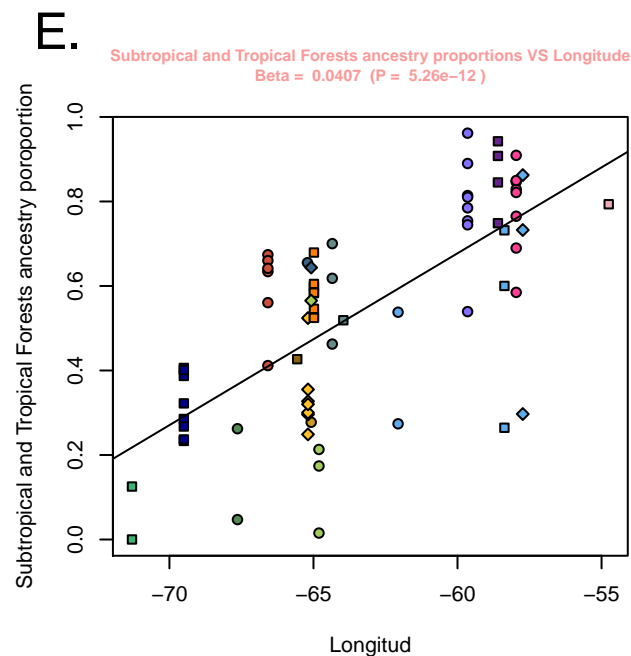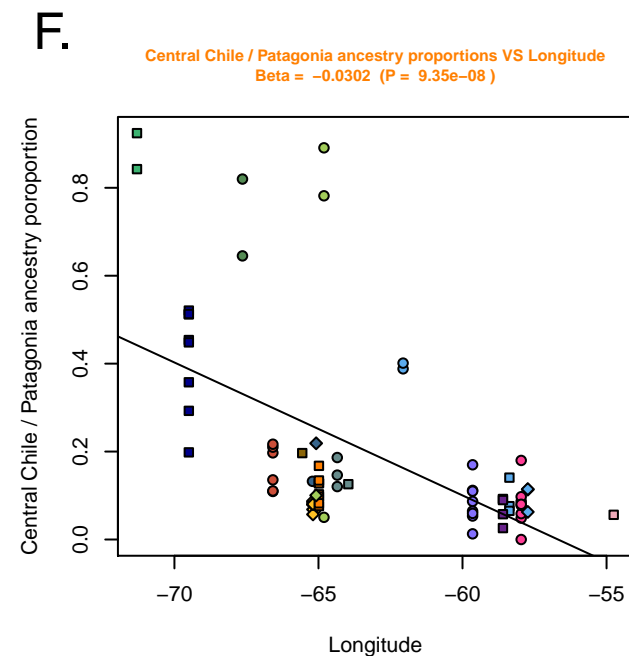

Supplement: S15 Fig — (A) Central Andes ancestry proportions vs Latitude. (B) Central Andes ancestry proportions vs Longitude. (C) Subtropical and Tropical Forests ancestry proportions vs Latitude. (D) Subtropical and Tropical Forests ancestry proportions vs Longitude. (E) Central Chile/Patagonia ancestry proportions vs Latitude. (F) Central Chile/Patagonia ancestry proportions vs Longitude. Linear regression slopes and the associated P-values are shown. (PDF) [file pone.0233808.s015.pdf]

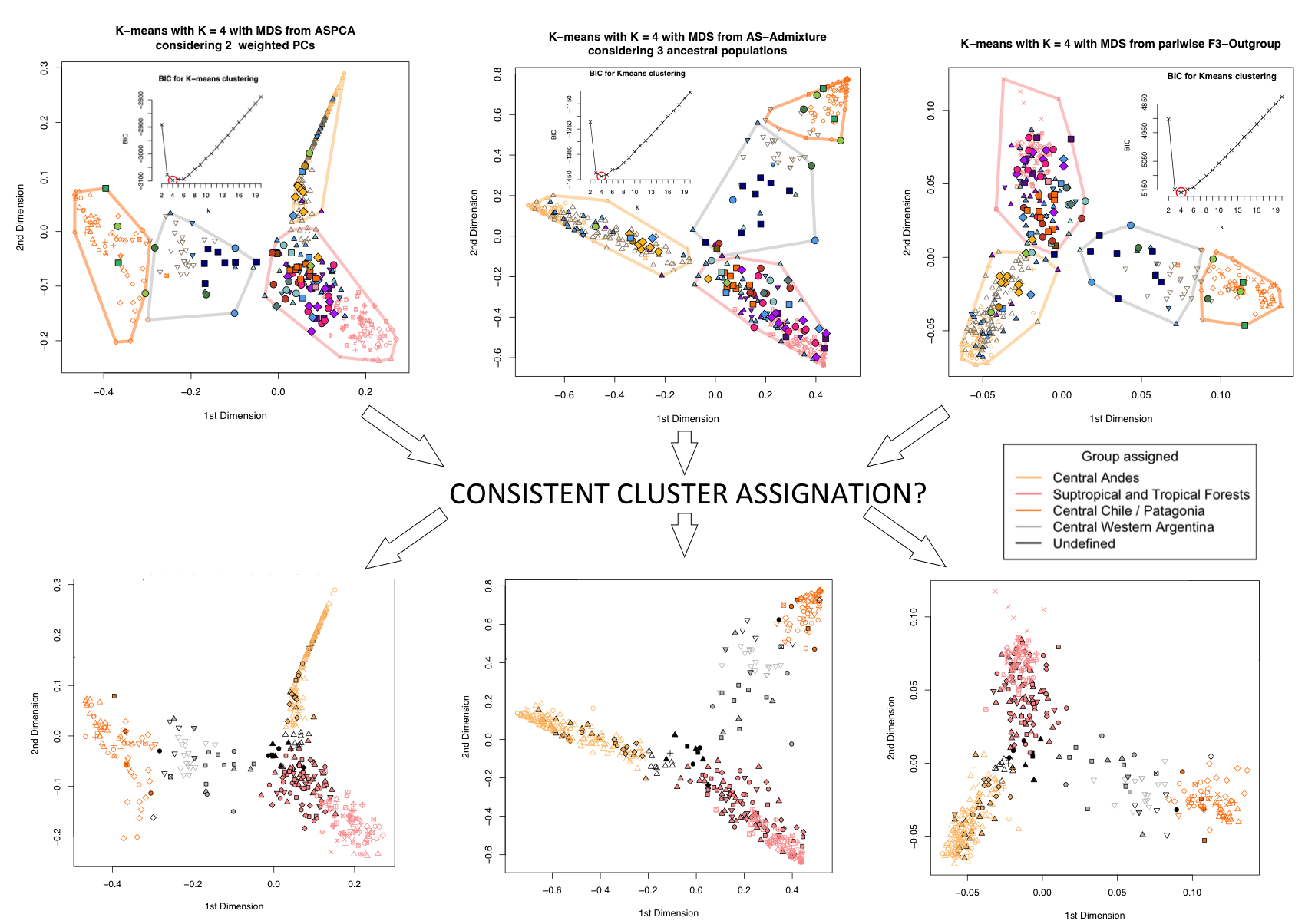

Supplement: S16 Fig — Consensus cluster assignation of South American individuals based on three K-means procedures run with different pairwise distances among individuals. (Top) K-means results using Ancestry-Specific PCA and Admixture (ASPCA and AS-Admixture), and f3 results to compute pairwise distances. Individuals are represented as in Main Fig 5. Insets: BIC score for number of clusters set to K-means ranging from 2 to 20. In all the three cases, K-means BIC was minimized when considering 4 clusters. (Bottom) Same as top with point colors corresponding to the assigned cluster. (TIF) [file pone.0233808.s016.tif]

# **1-f3(YRI;Ind1,Ind2)** with Ind1 and Ind2 belonging to Group1 and Group2, respectively

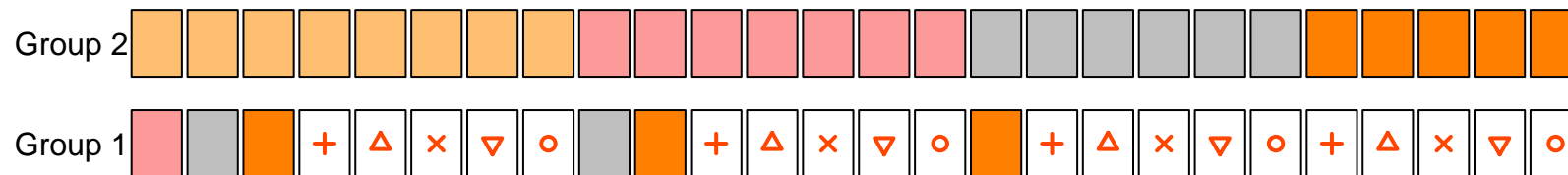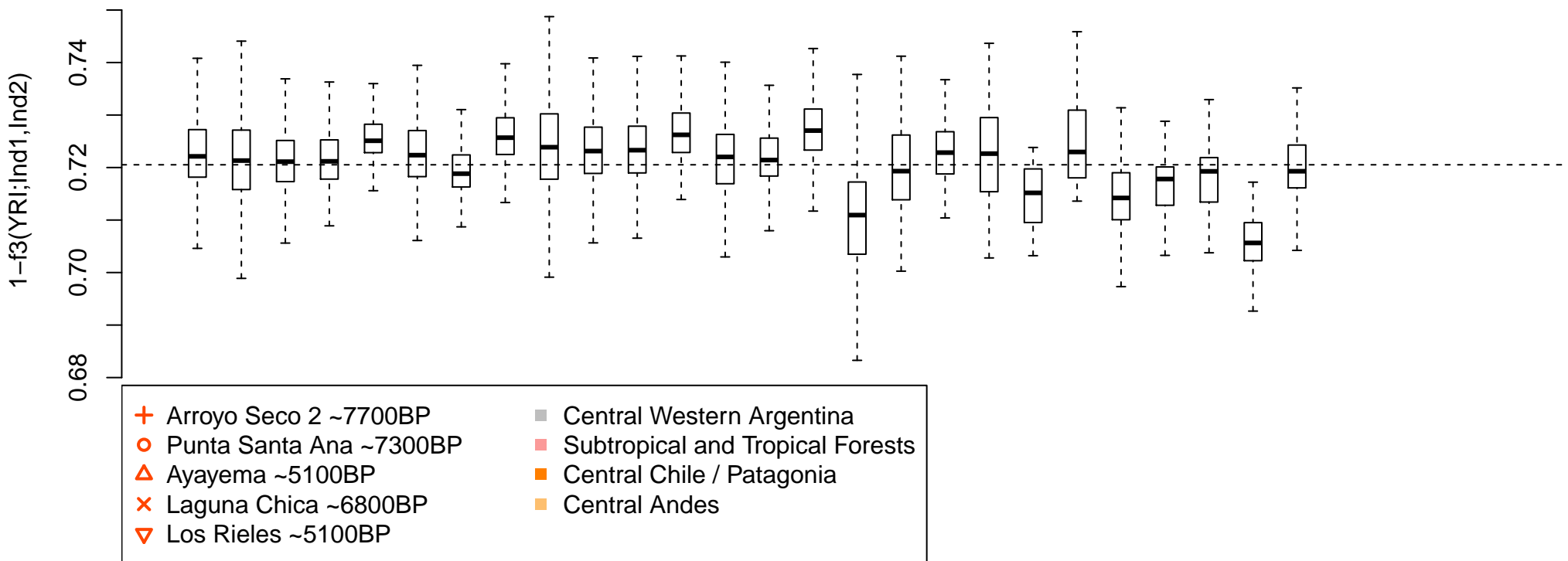

Supplement: S17 Fig — Boxplots for 1- f3(YRI; Ind1, Ind2), where Ind1 and Ind2 are two individuals belonging to Group 1 and Group 2, respectively. The groups are either the fourth Native American components identified or ancient Middle Holocene Southern Cone groups. For clarity, boxplot outliers are not shown. YRI: Yoruba from 1KGP. (PDF) [file pone.0233808.s017.pdf]

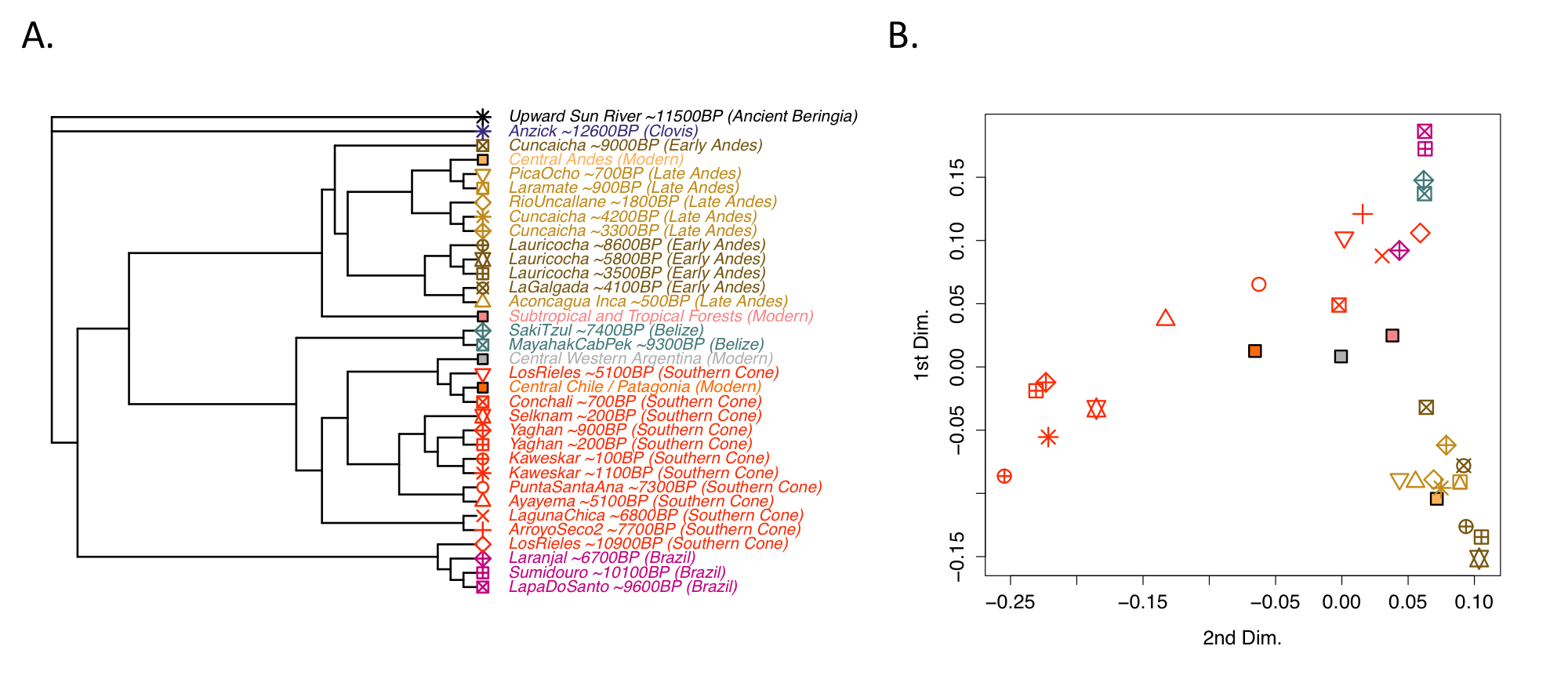

Supplement: S18 Fig — (A) Neighbor-joining tree from distances of the form 1/f3(YRI; X, Y). USR1 from Ancient Beringia was used as outgroup (B) Multidimensional-scaling from distances of the form 1-f3(YRI; X, Y). Each group is represented as appearing in the leaf of (A). USR1 and Anzick-1 were not considered in (B). YRI: Yoruba from 1KGP. (TIF) [file pone.0233808.s018.tif]

A.

f3(YRI; CAN, Ancient)

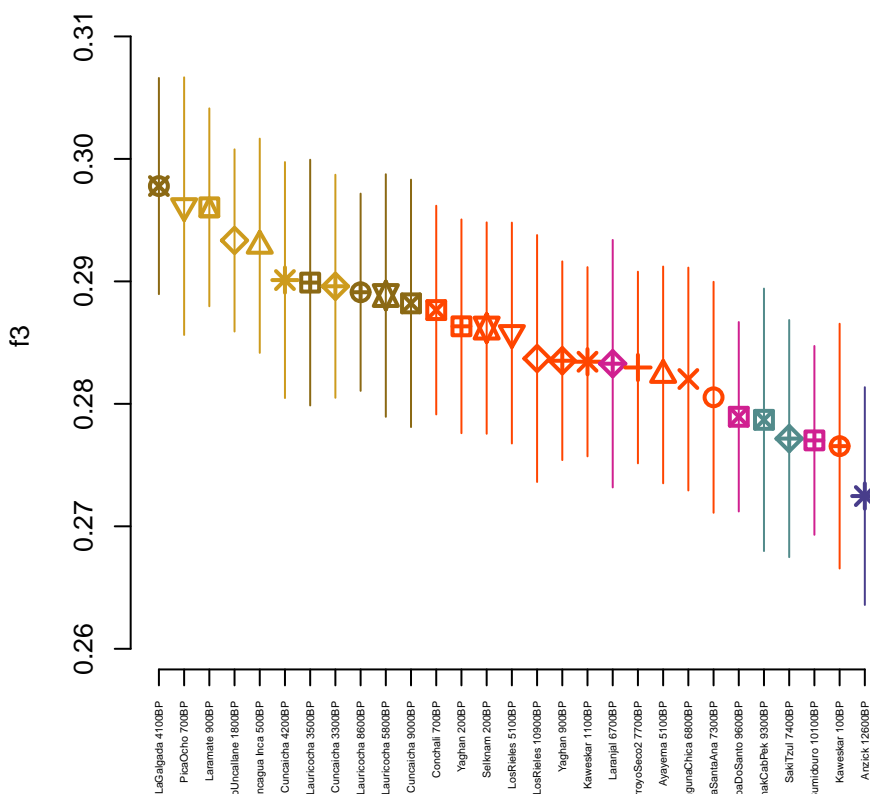

B.

f3(YRI; STF, Ancient)

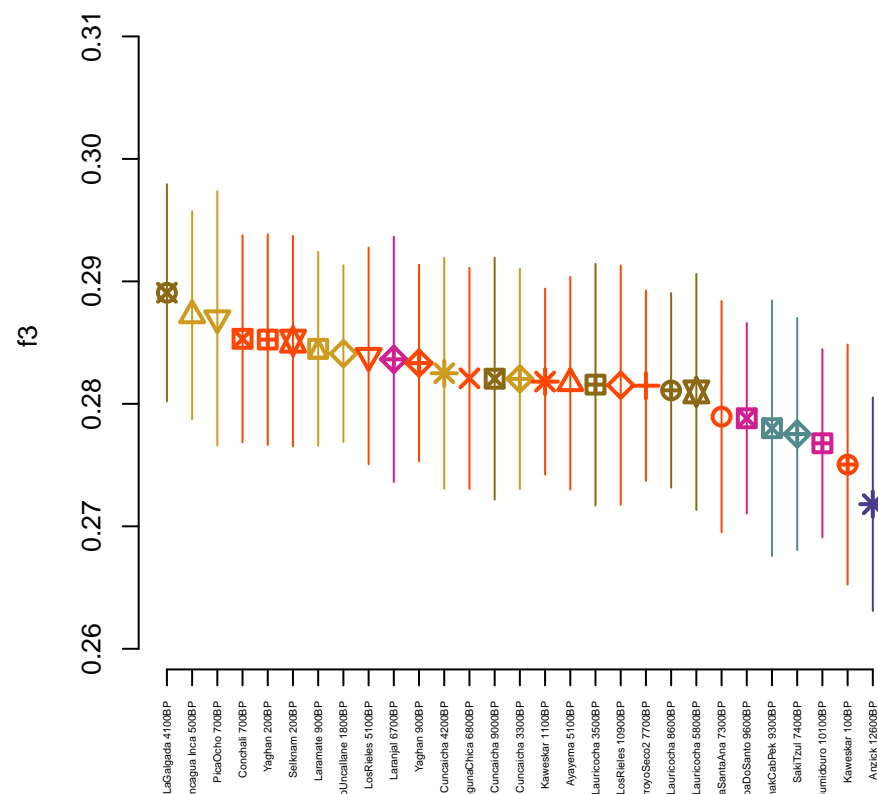

C.

f3(YRI; CCP, Ancient)

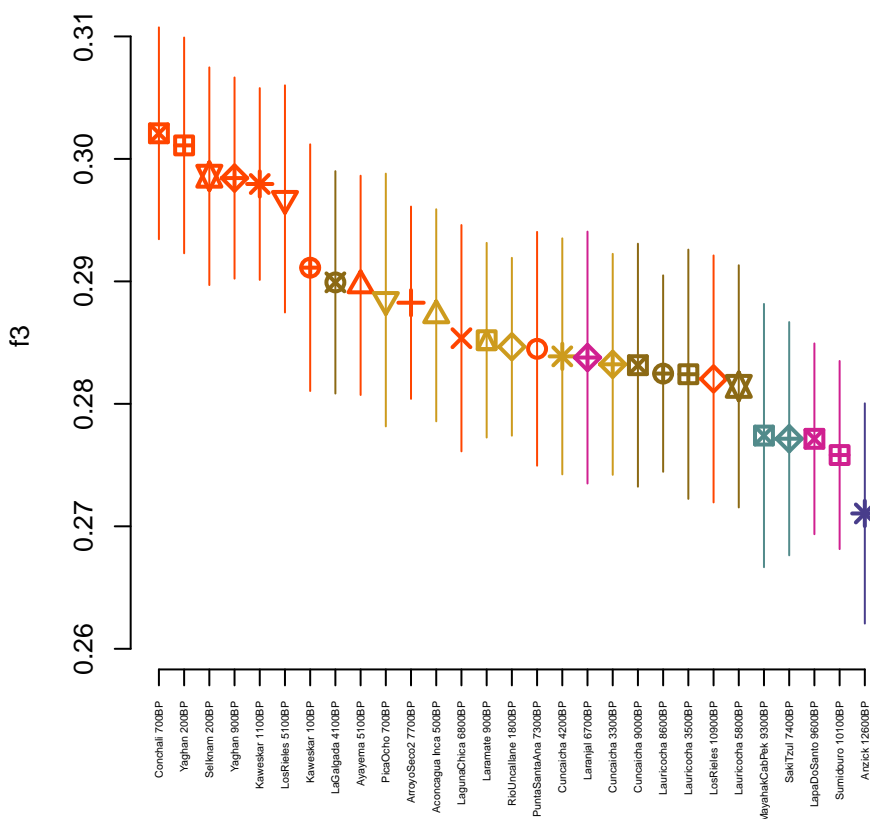

D.

f3(YRI; CWA, Ancient)

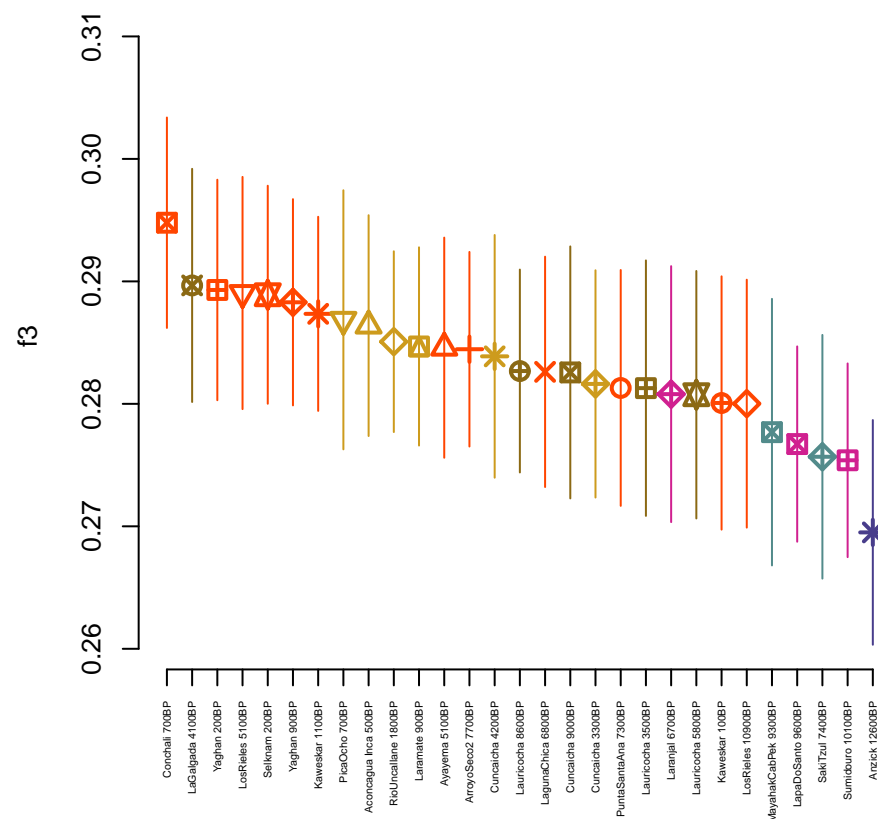

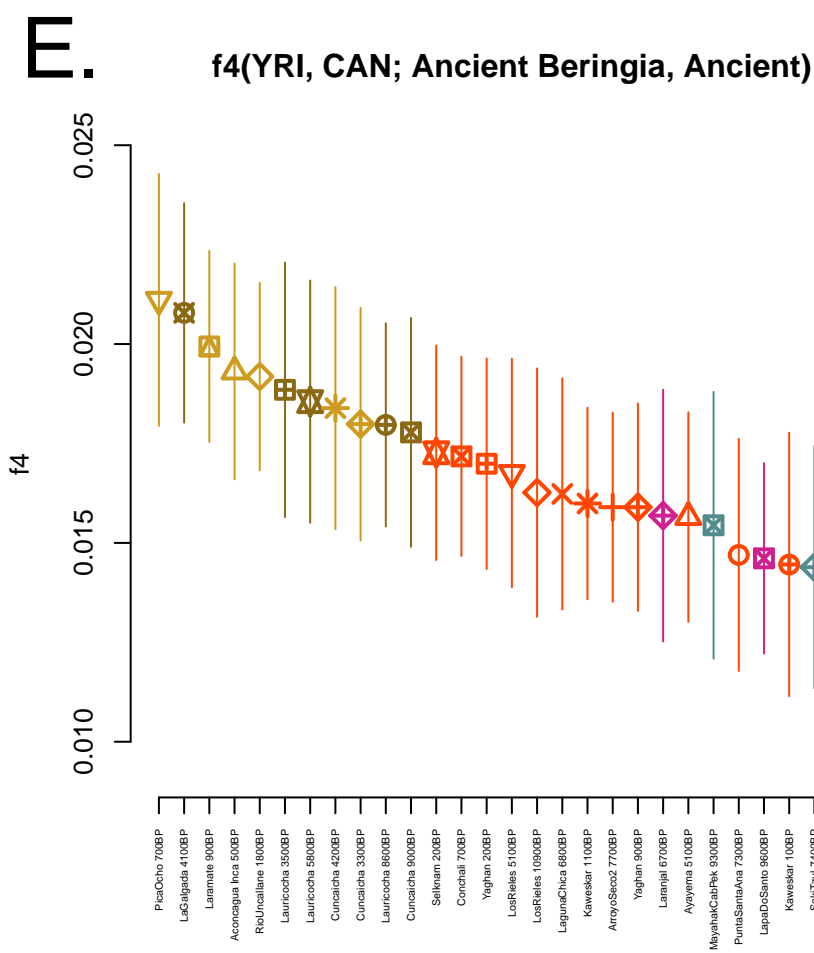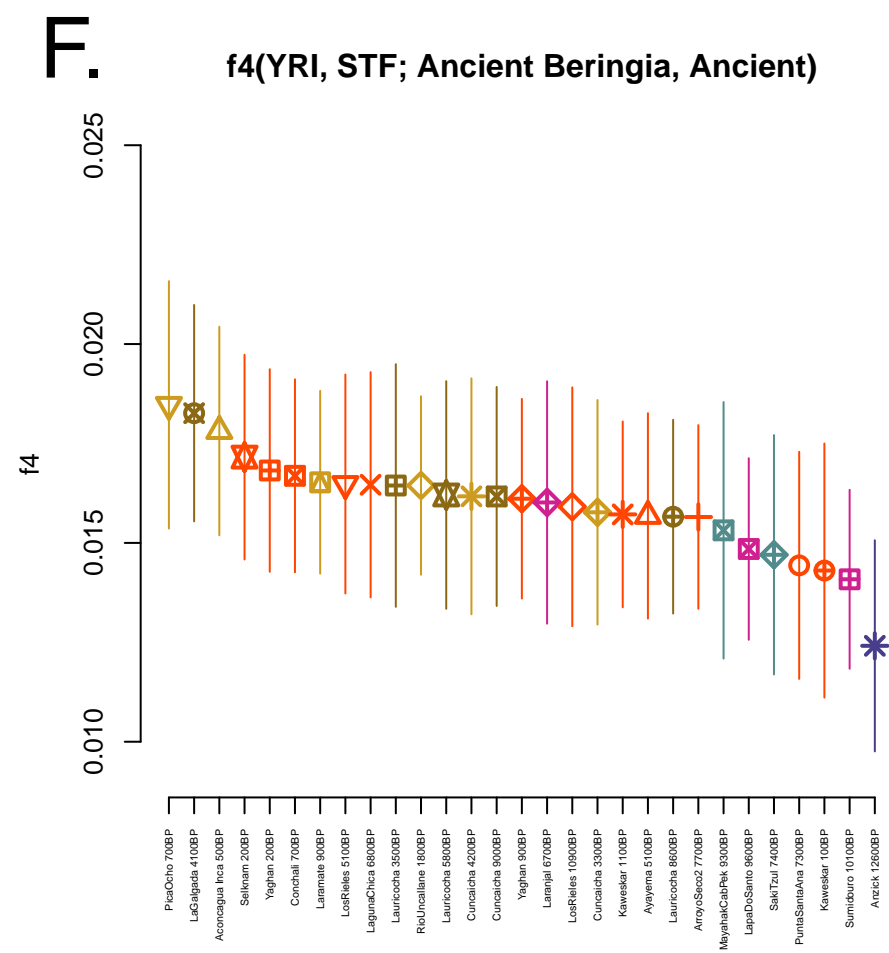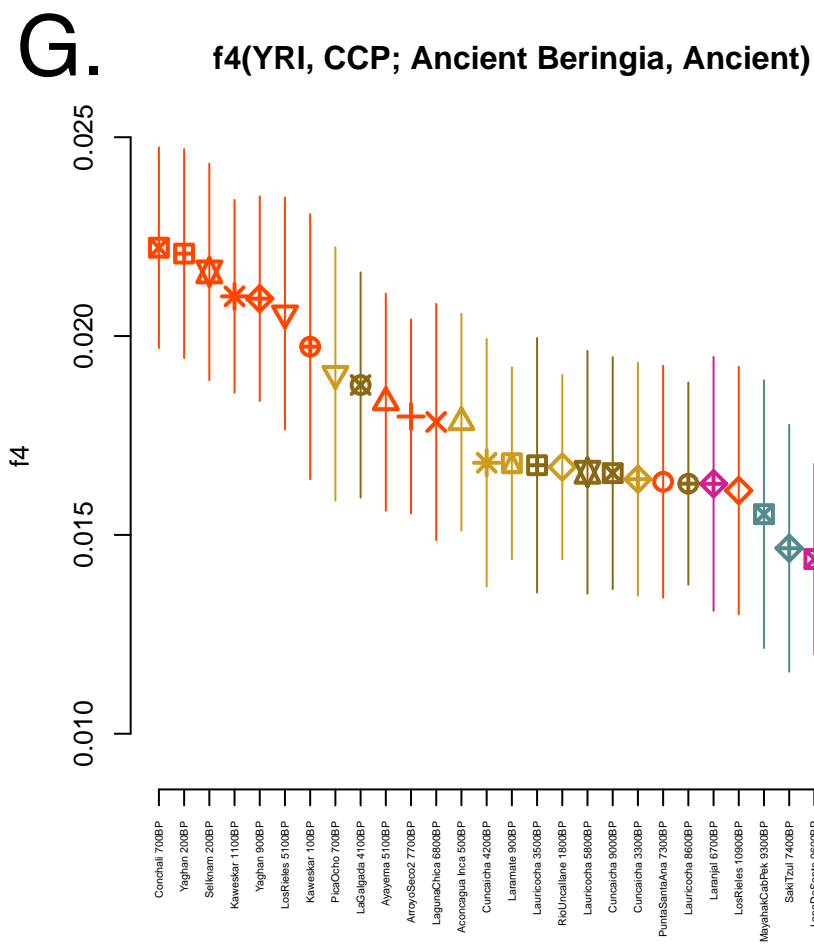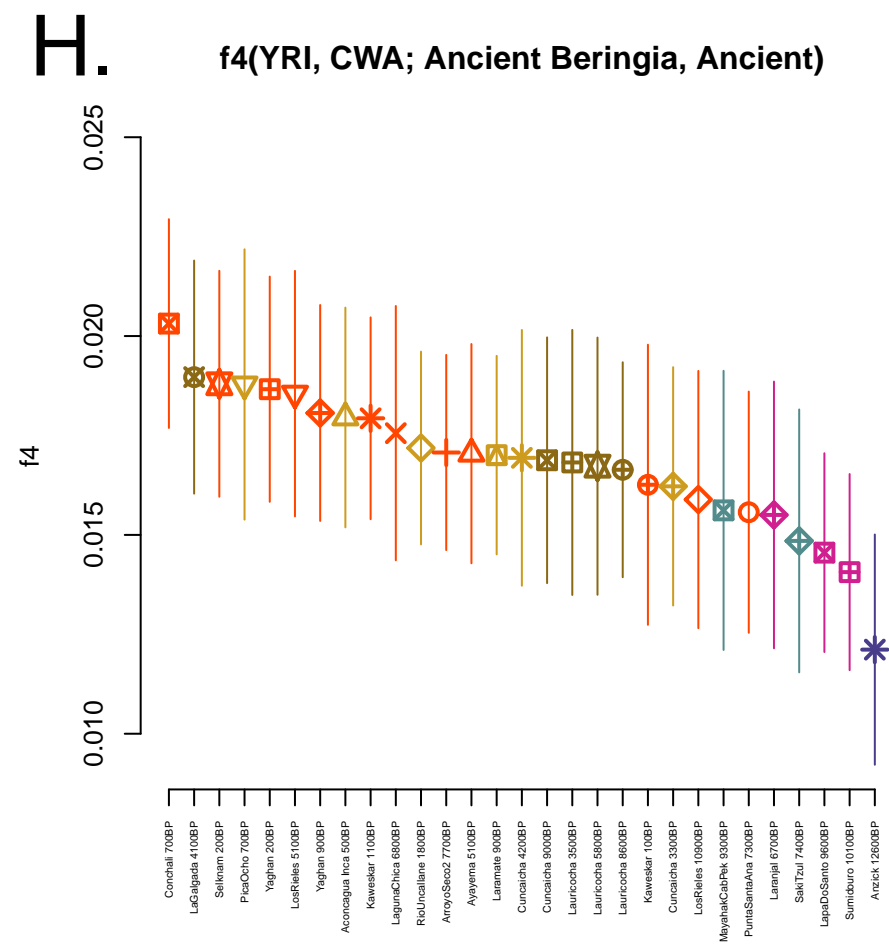

Supplement: S19 Fig — (A-D) f3(YRI; X; Ancient). (E-H) f4(YRI, X; Ancient Beringia, Ancient). (A) and (E): with Central Andes (CAN) as X. (B) and (F): With Subtropical and Tropical Forests (STF) as X. (C) and (G): With Central Chile / Patagonia (CCP) as X. (D) and (H): With Central Western Argentina (CWA) as X. YRI: Yoruba from 1KGP; Ancient Beringia: USR1 individual from [66]; X: Native American component in Argentina (one plot per X). Ancient: ancient group labeled on the x-axis and represented with a point/color scheme as in Main Fig 5. Vertical segments are the +/- 3 standard errors intervals. (PDF) [file pone.0233808.s019.pdf]

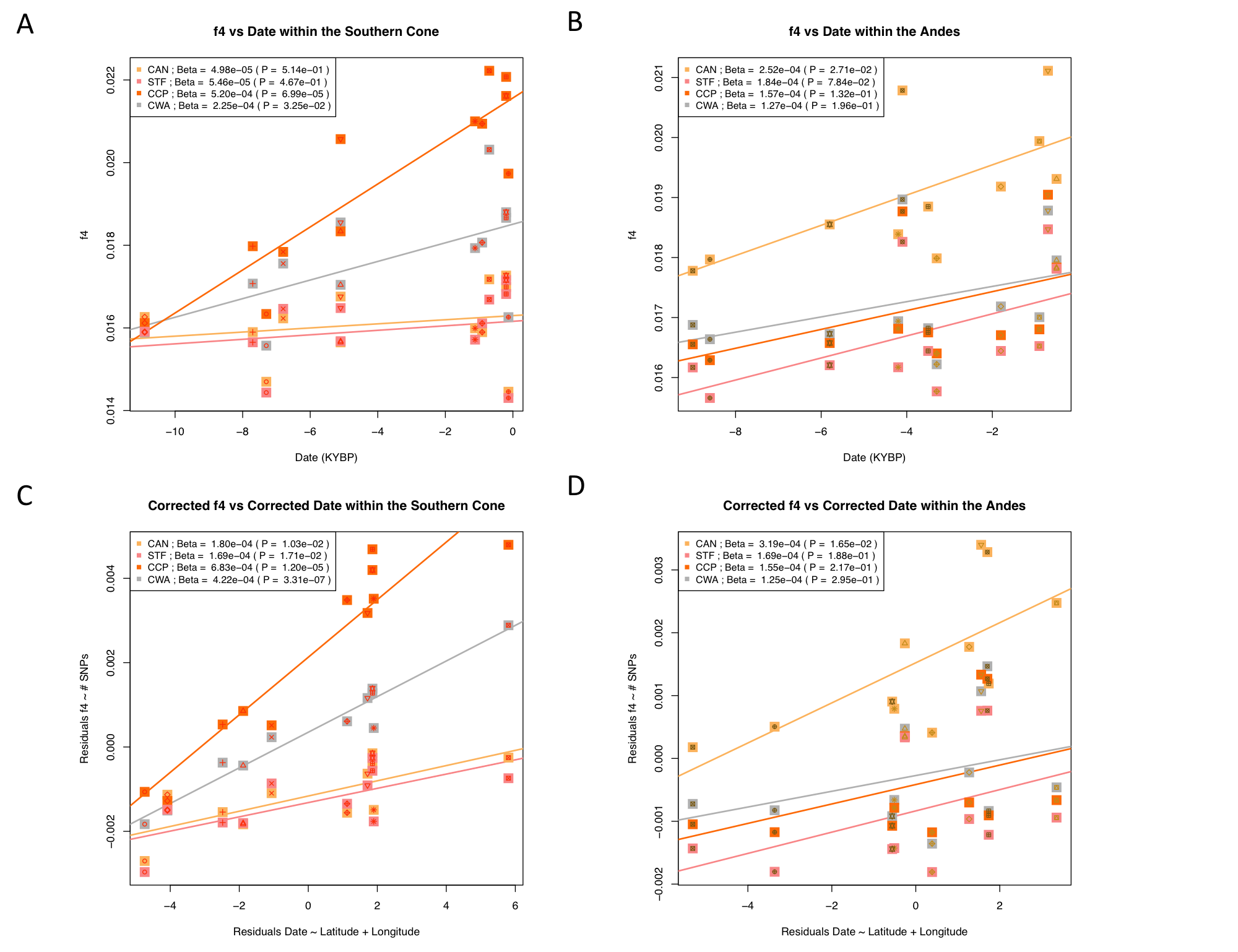

Supplement: S20 Fig — Each point represents a f4 score of the form f4(YRI, X; Ancient Beringia, Ancient) vs the age of ancient sample, where X is one of the four identified Native American components, and Ancient is an ancient group. X is represented by the color of the square while Ancient is represented by the point within the square. The point code of the ancient samples is shown in Main Fig 5. Ancient Beringia: USR1 individual from [66]. (A) f4 vs age of ancient samples from Southern Cone. (B) f4 vs age of ancient samples from Andes. (C) f4 vs age of ancient samples from Southern Cone considering correction for both f4 and age. (D) f4 vs age of ancient samples from Andes considering correction for both f4 and age. Linear regression slopes and the associated P-values are shown. CAN: Central Andes; STF: Subtropical and Tropical Forests; CCP: Central Chile / Patagonia; CWA: Central Western Argentina; YRI: Yoruba from 1KGP. (TIF) [file pone.0233808.s020.tif]

A.

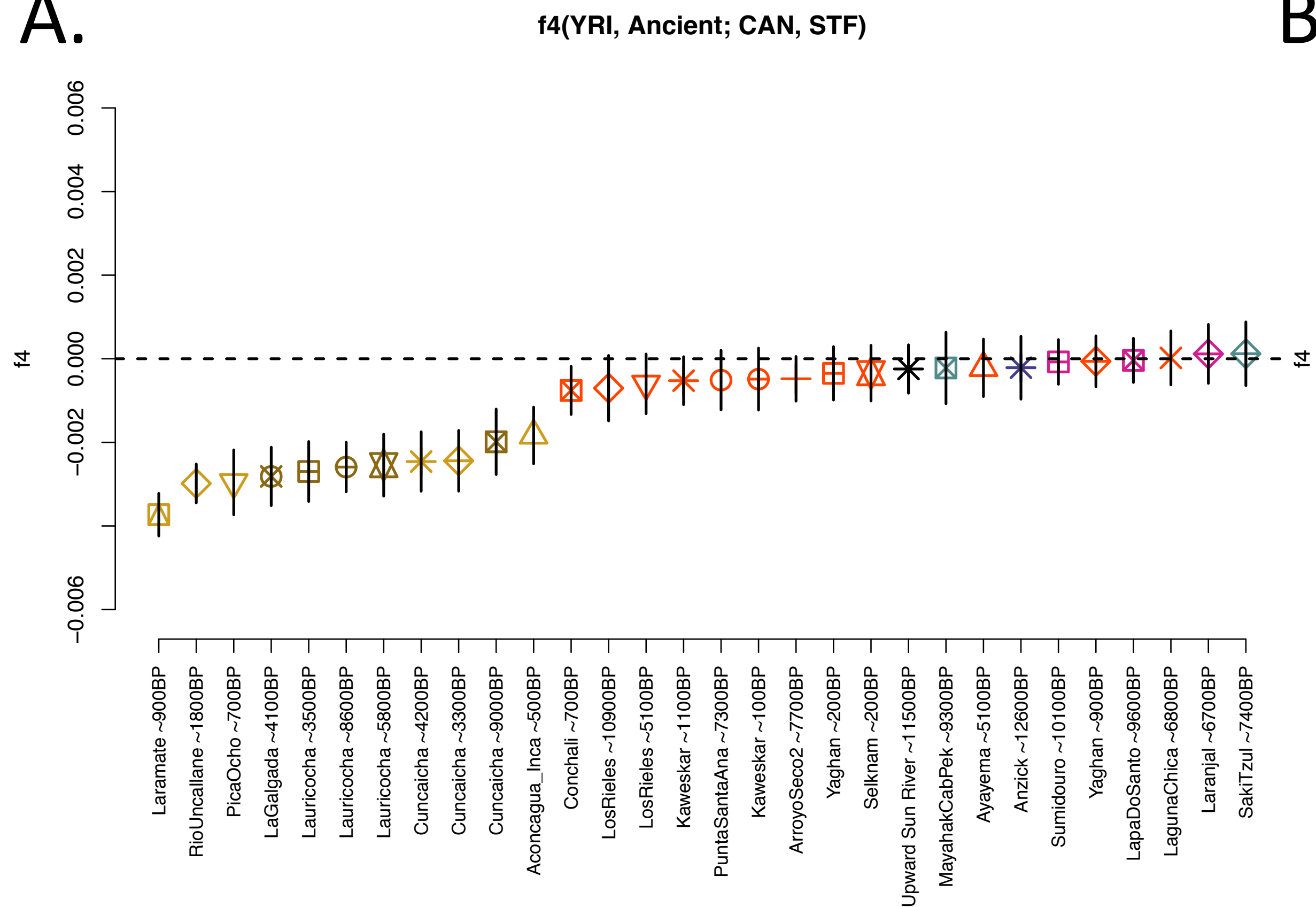

B.

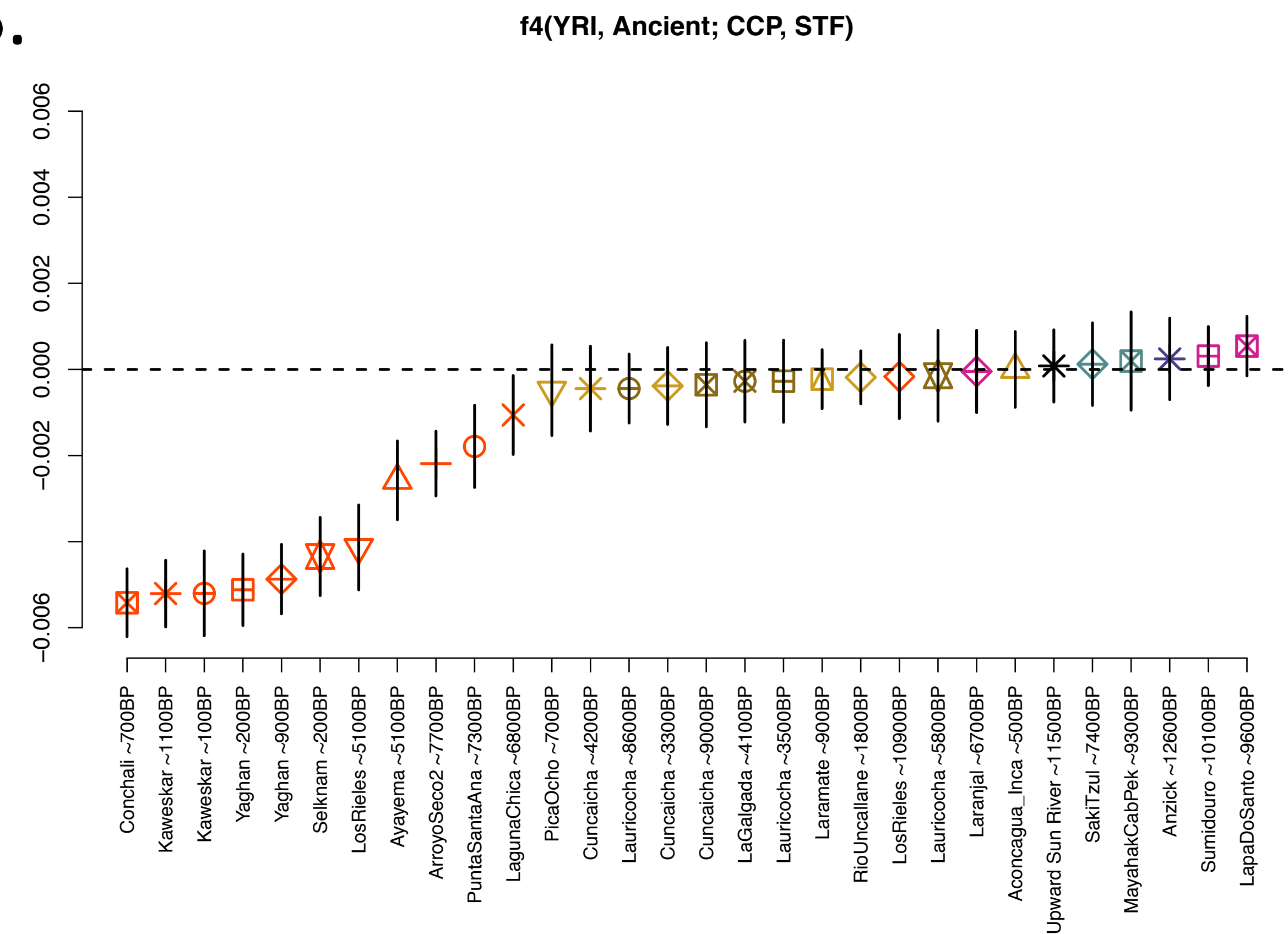

C.

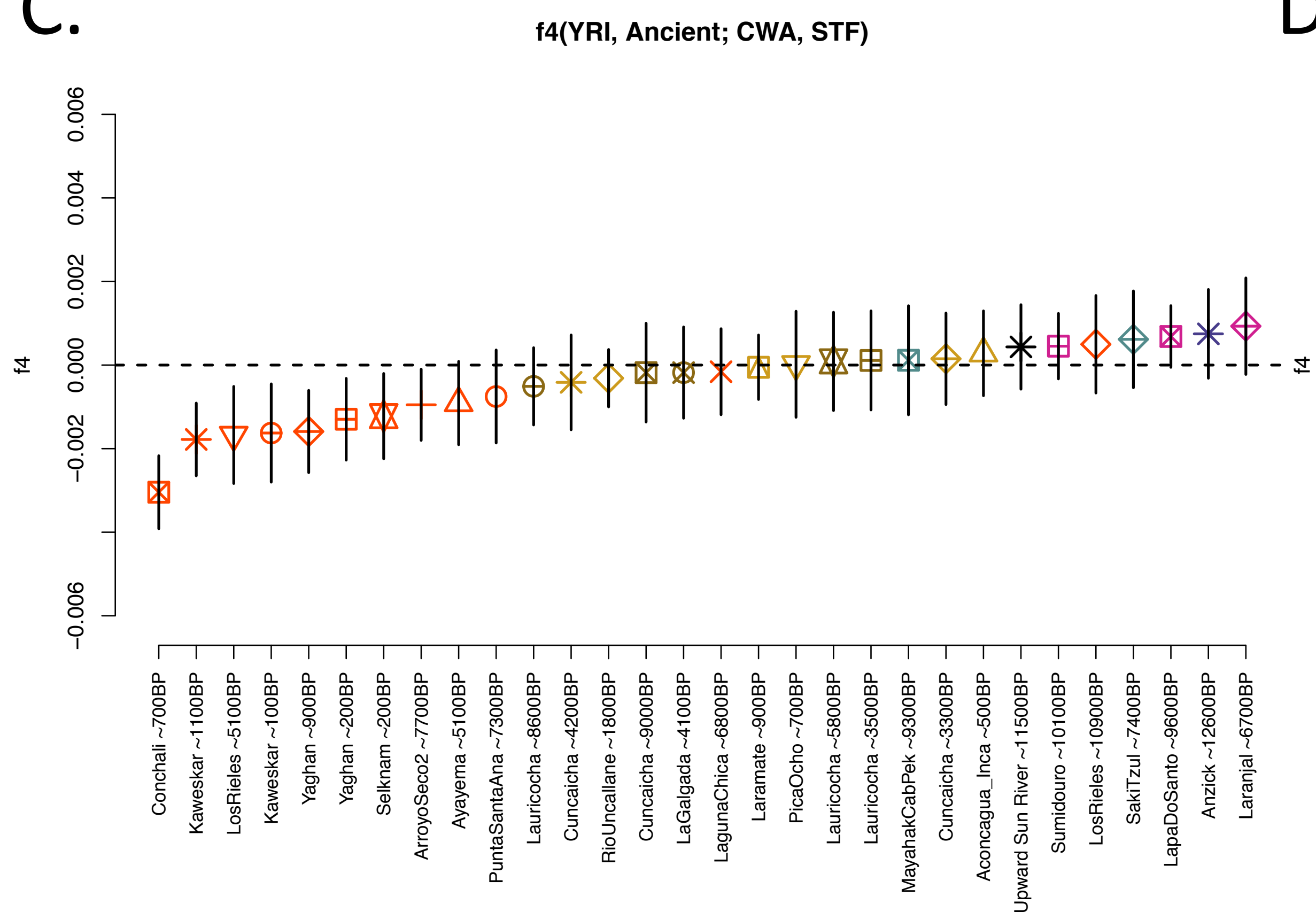

D.

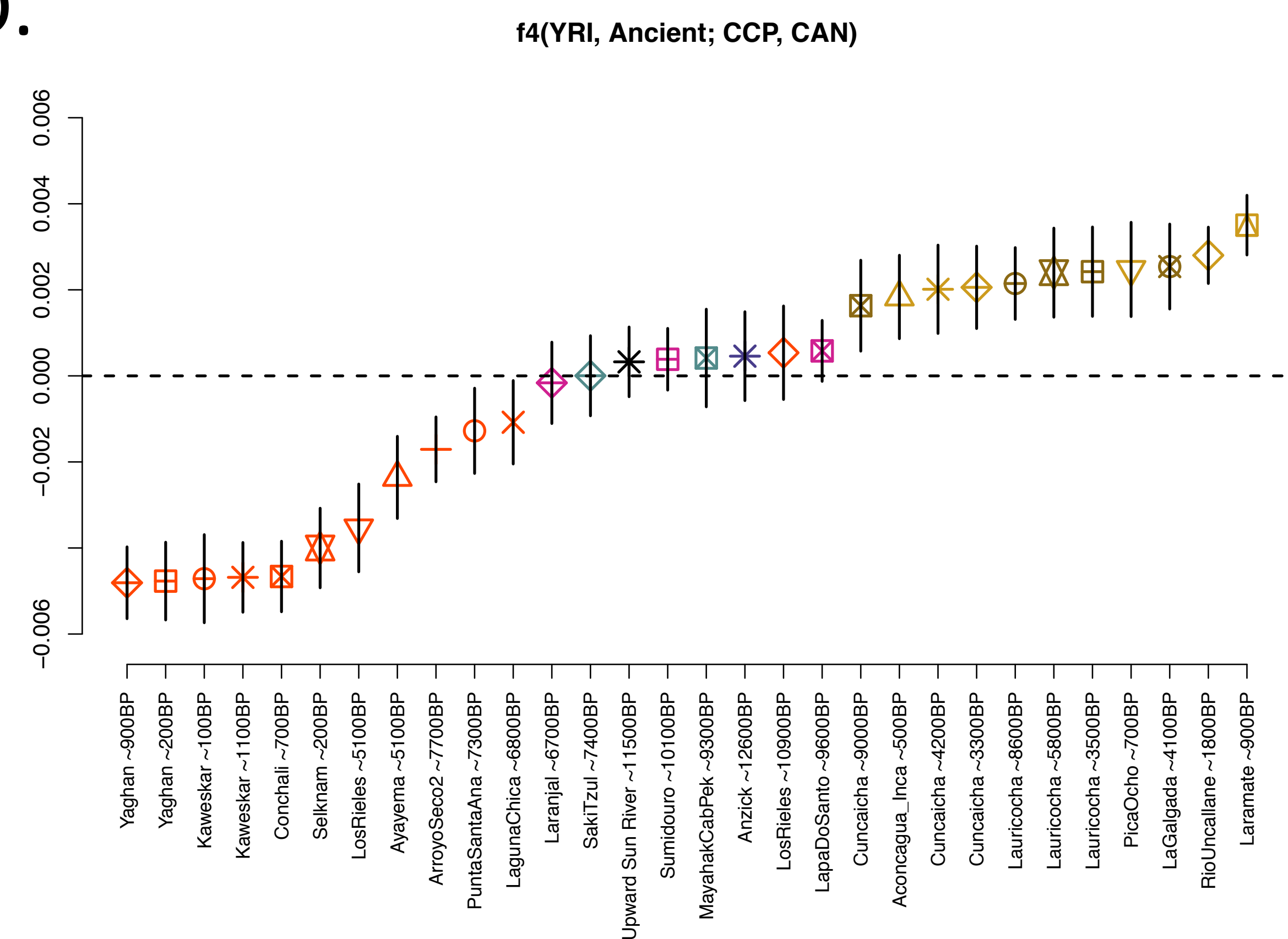

E.

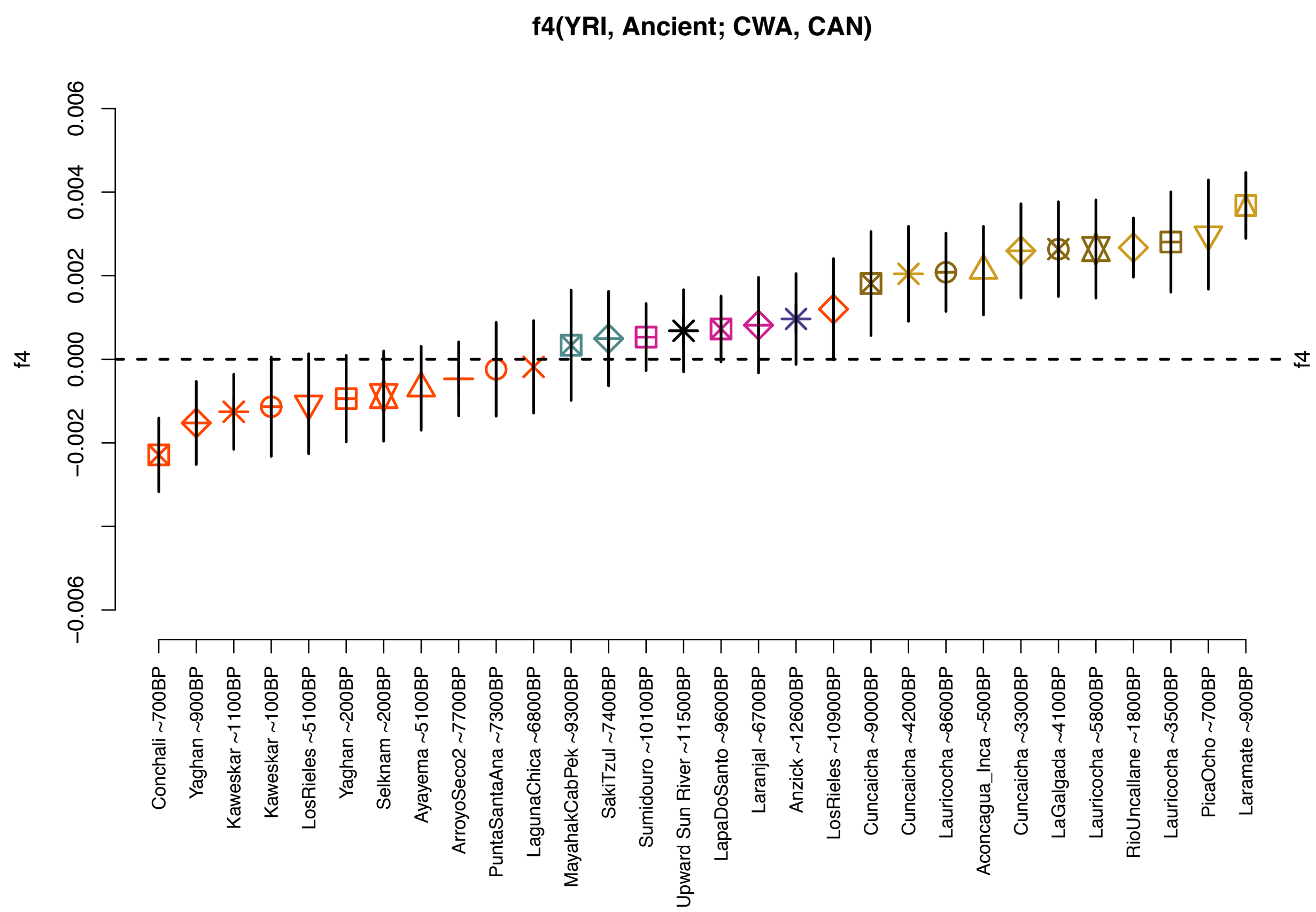

F.

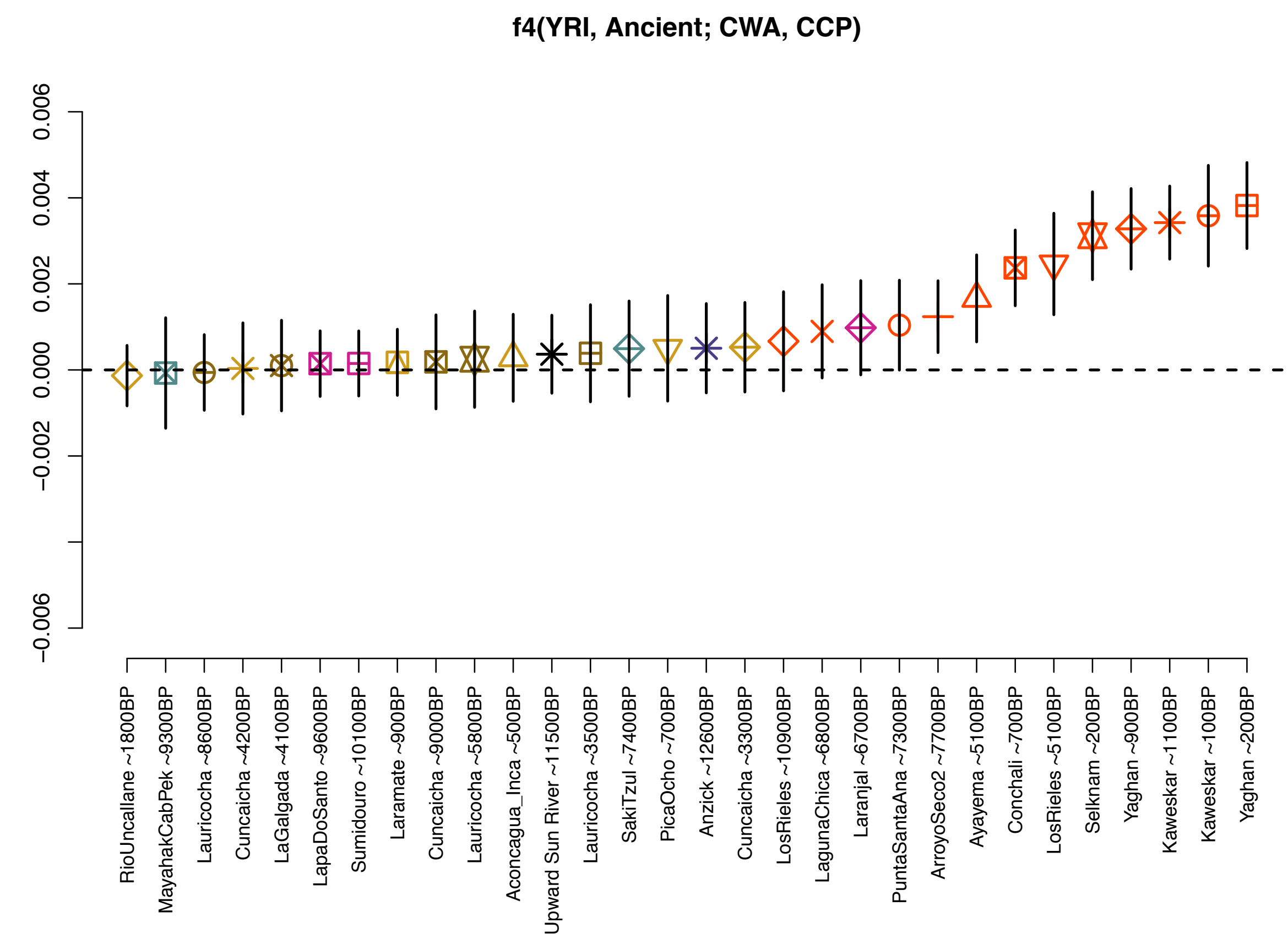

Supplement: S21 Fig — f4 (YRI, Ancient, X, Y) where X and Y are two of the four identified Native American components (one plot per X-Y combination), and Ancient is ancient group labeled on the x-axis and represented with a point/color scheme as in Main Fig 5. Vertical segments are the +/- 3 standard errors intervals. Note this setting for f4 statistics is symmetrical when switching X and Y. (PDF) [file pone.0233808.s021.pdf]

A.

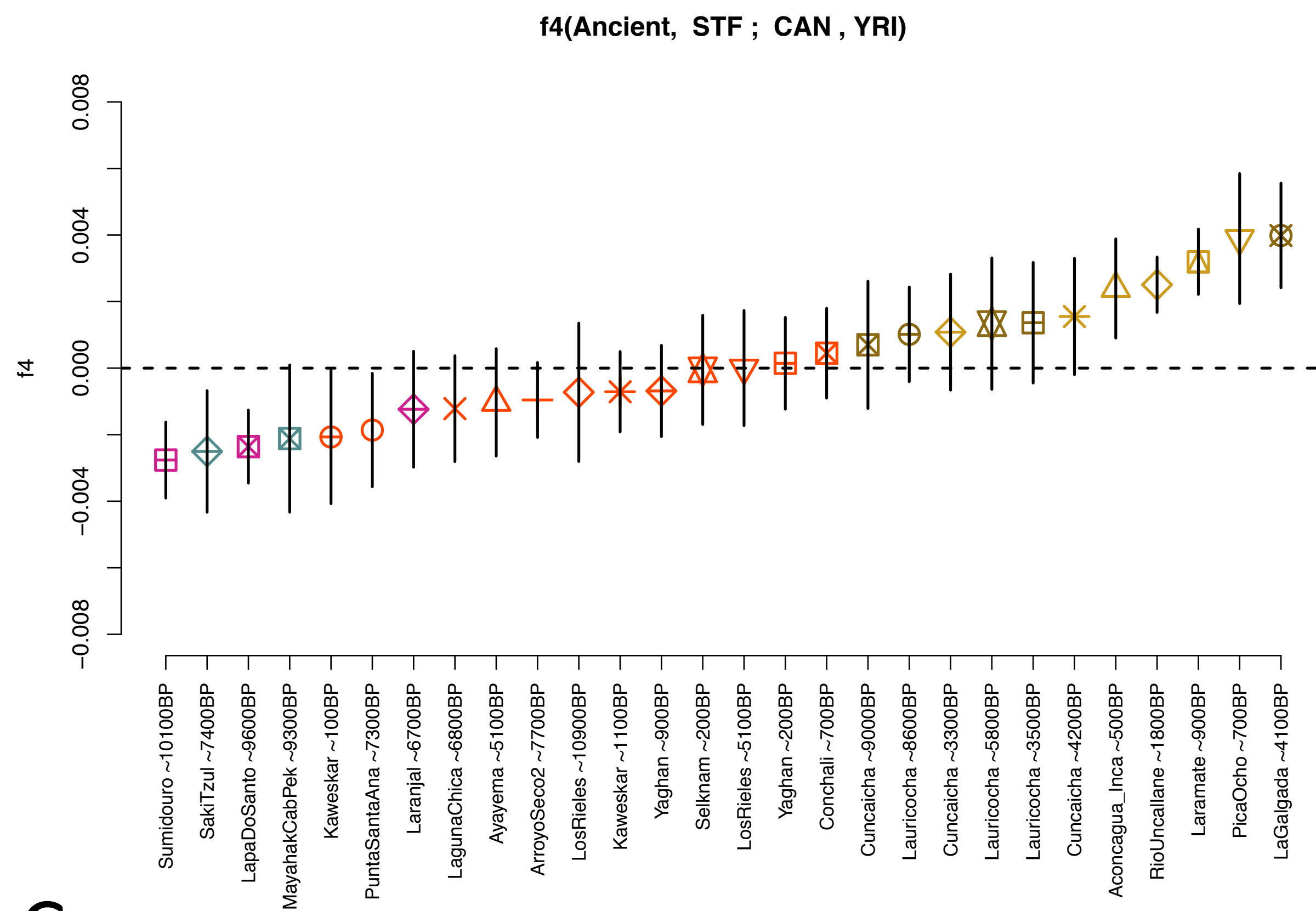

B.

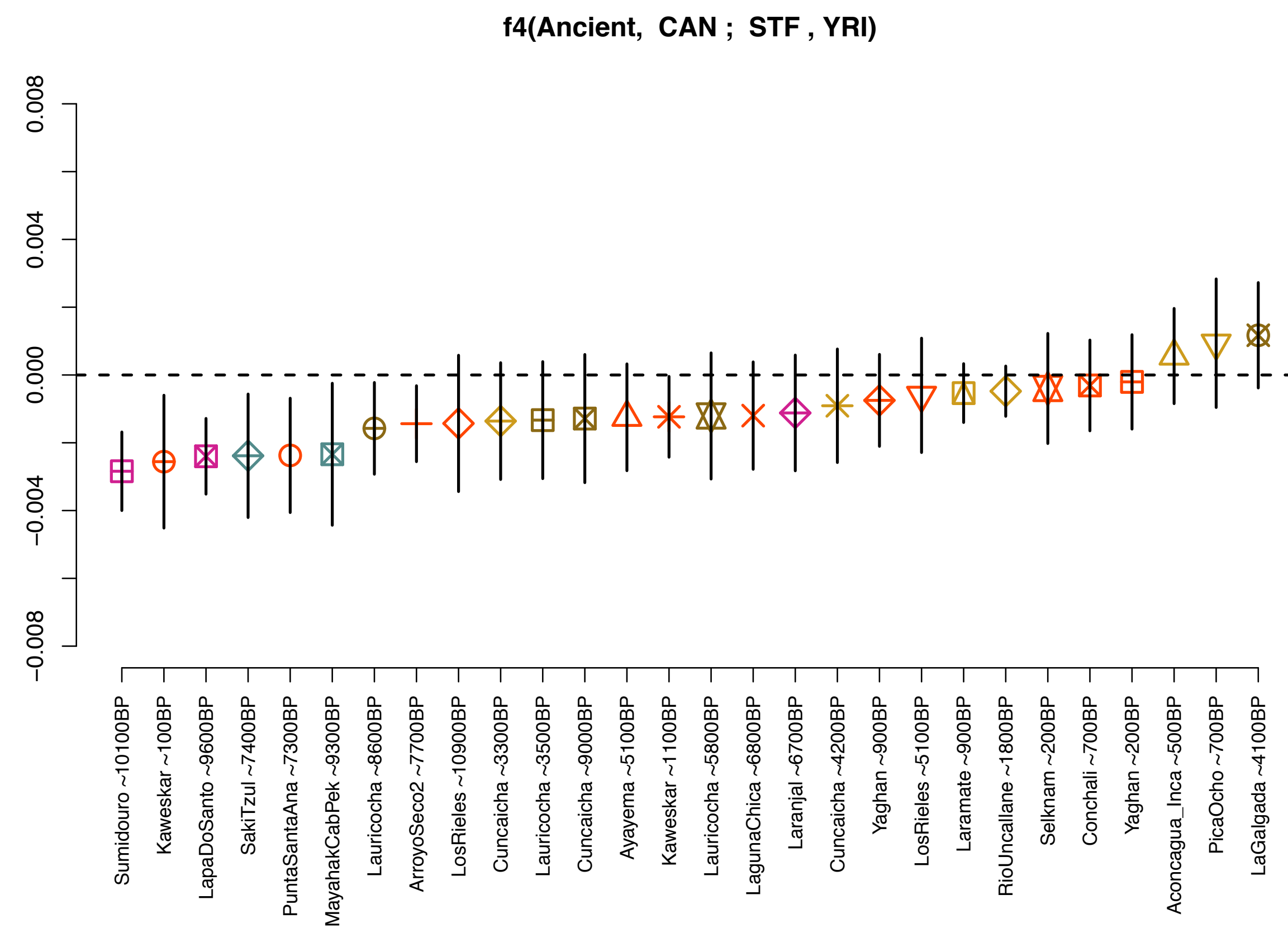

C.

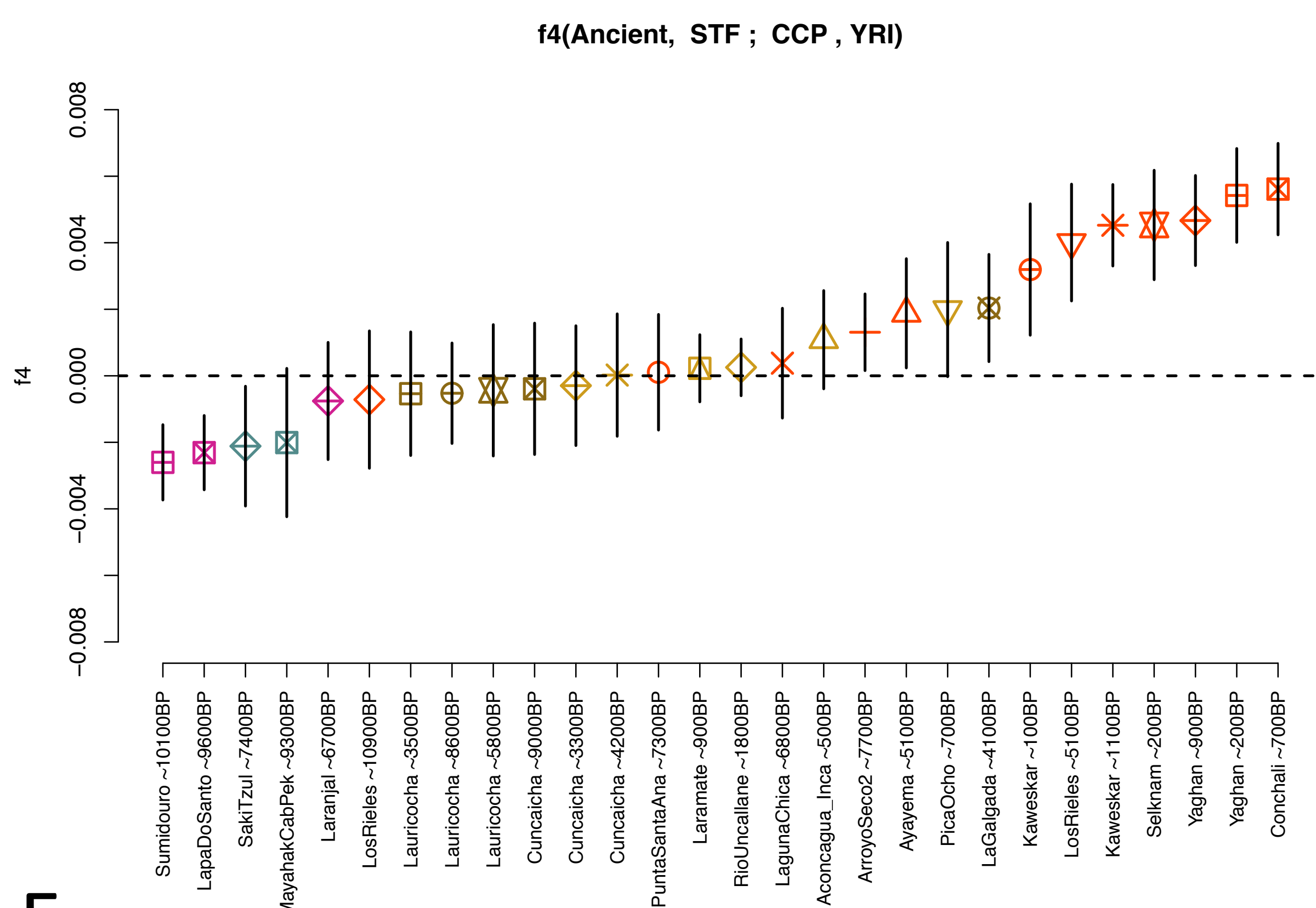

D.

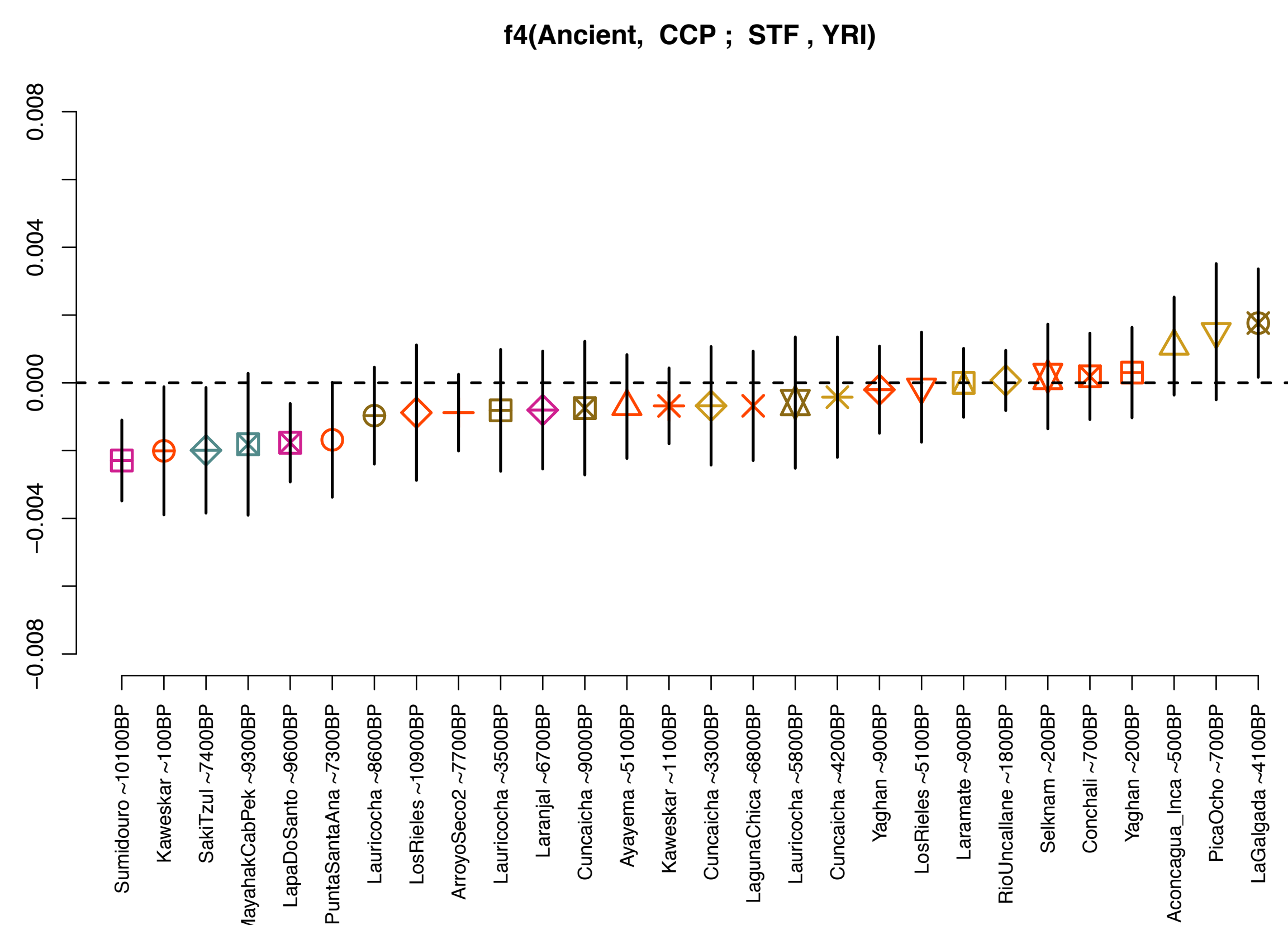

E.

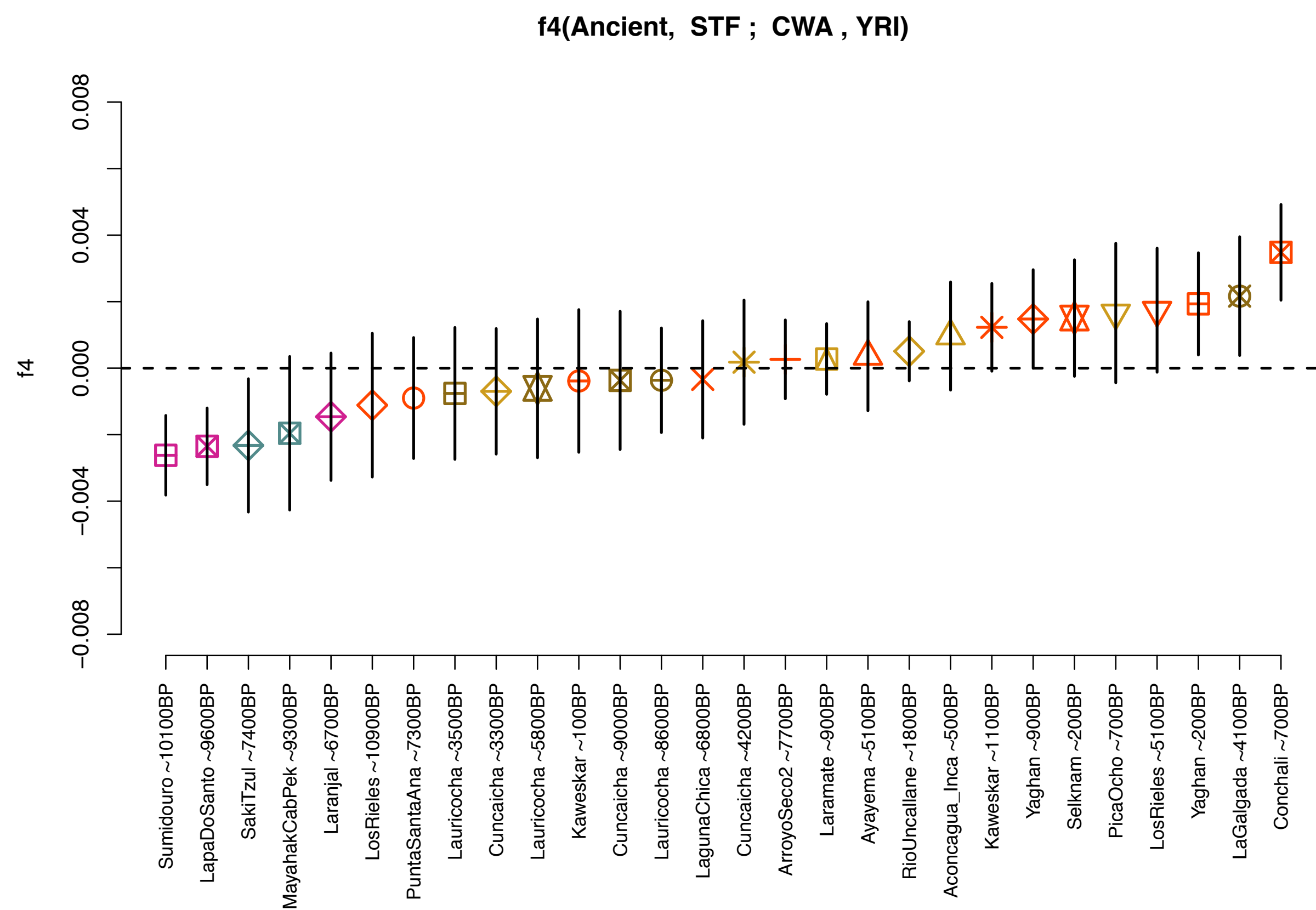

F.

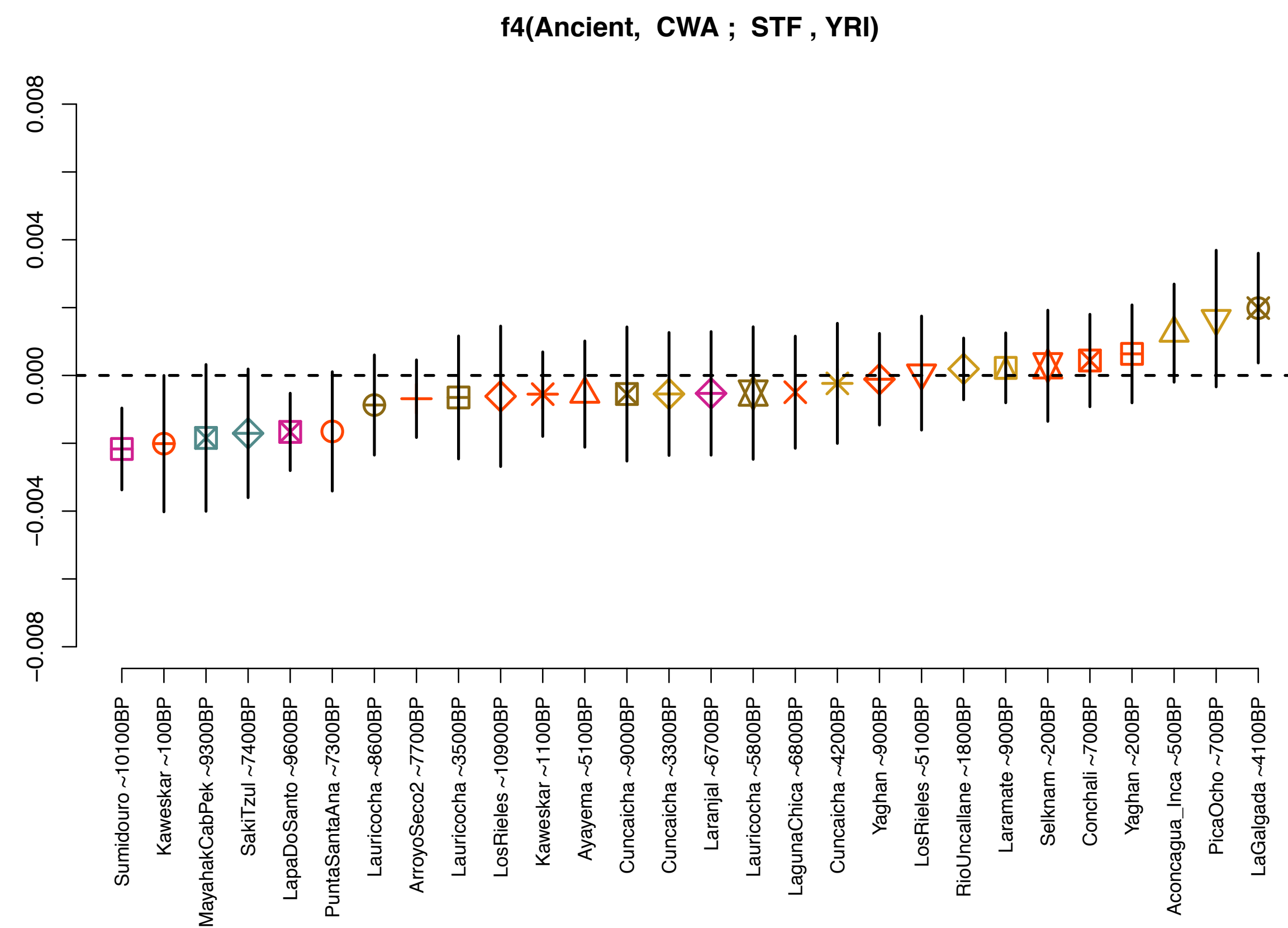

G.

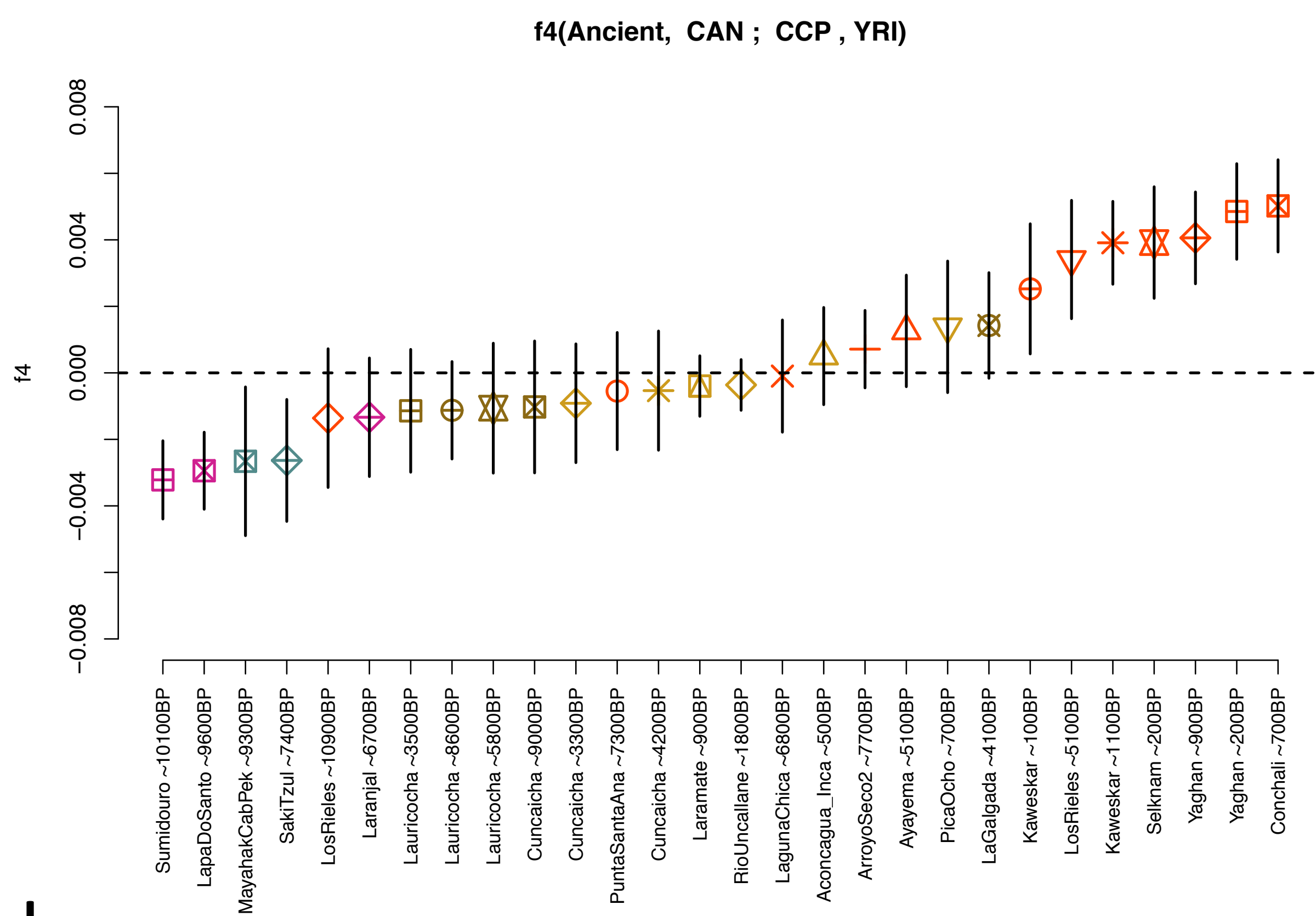

H.

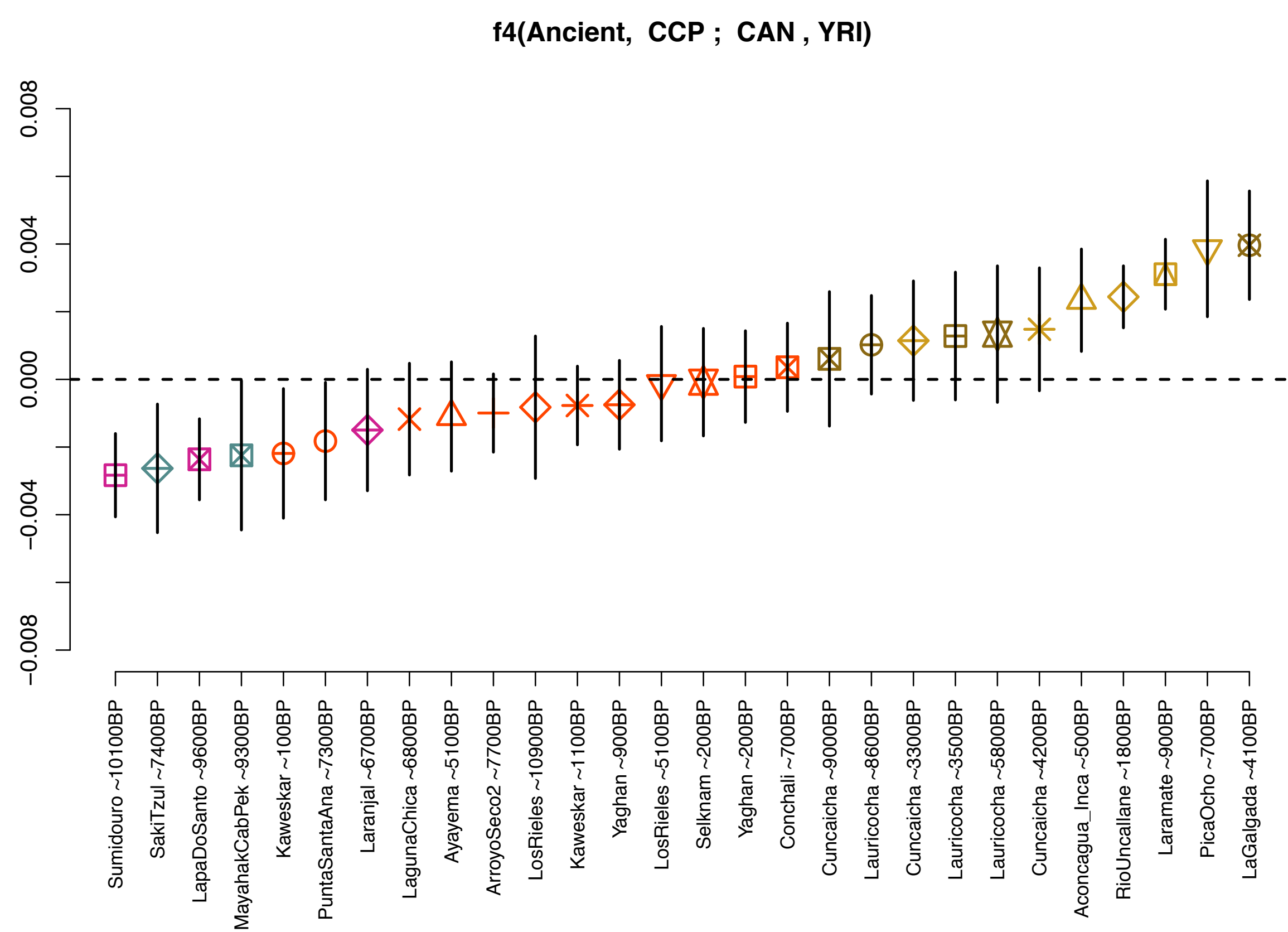

I.

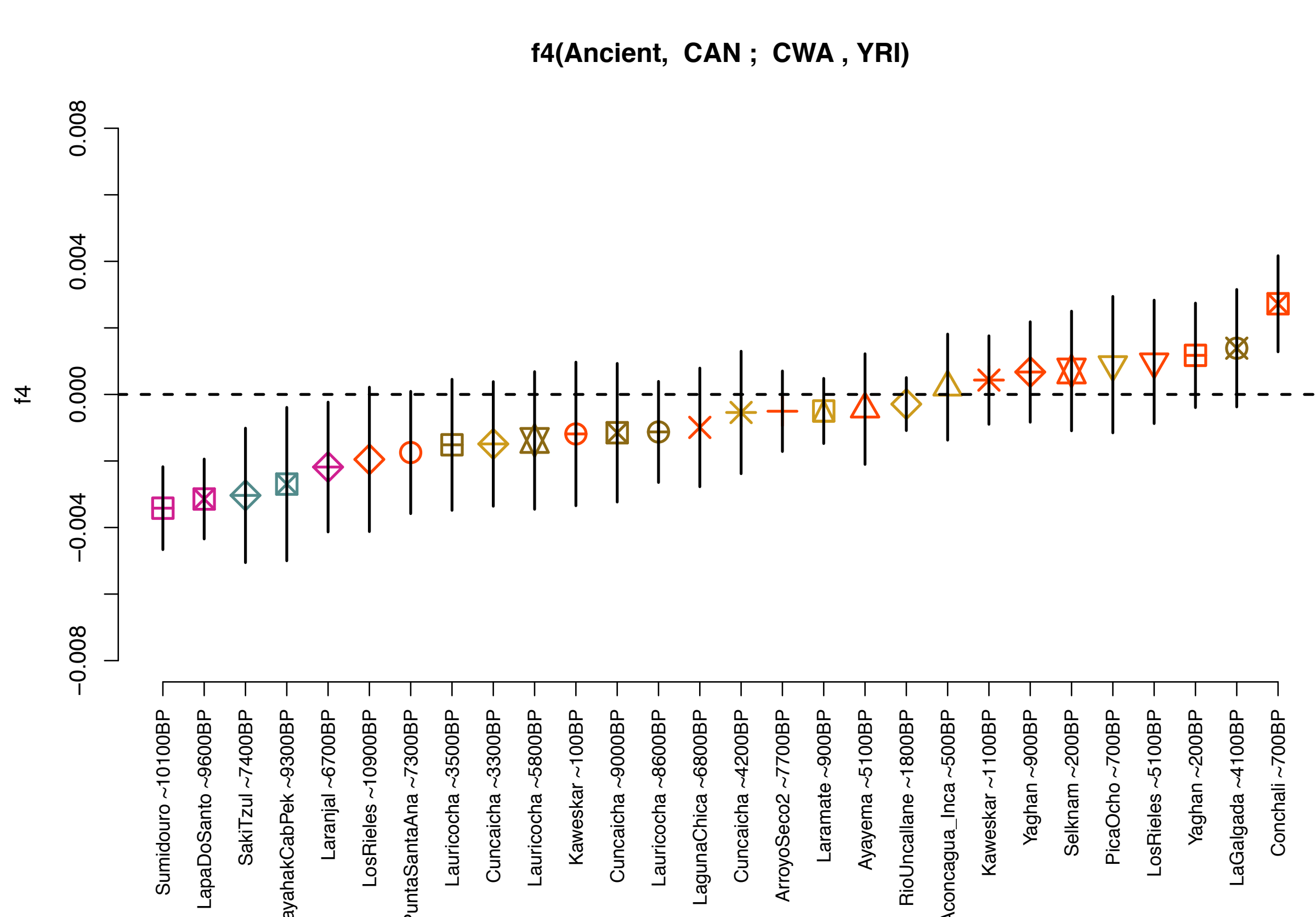

J.

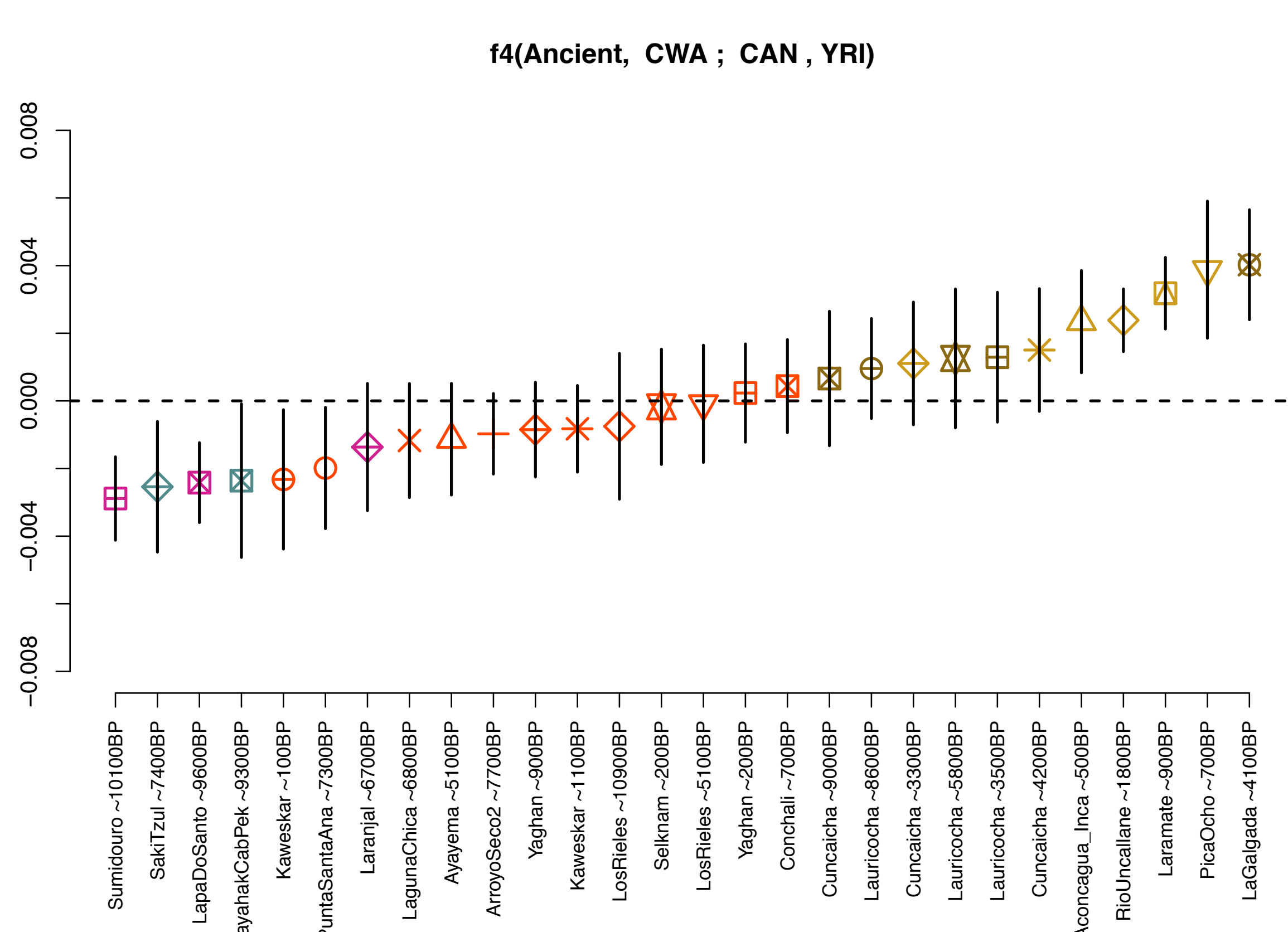

K.

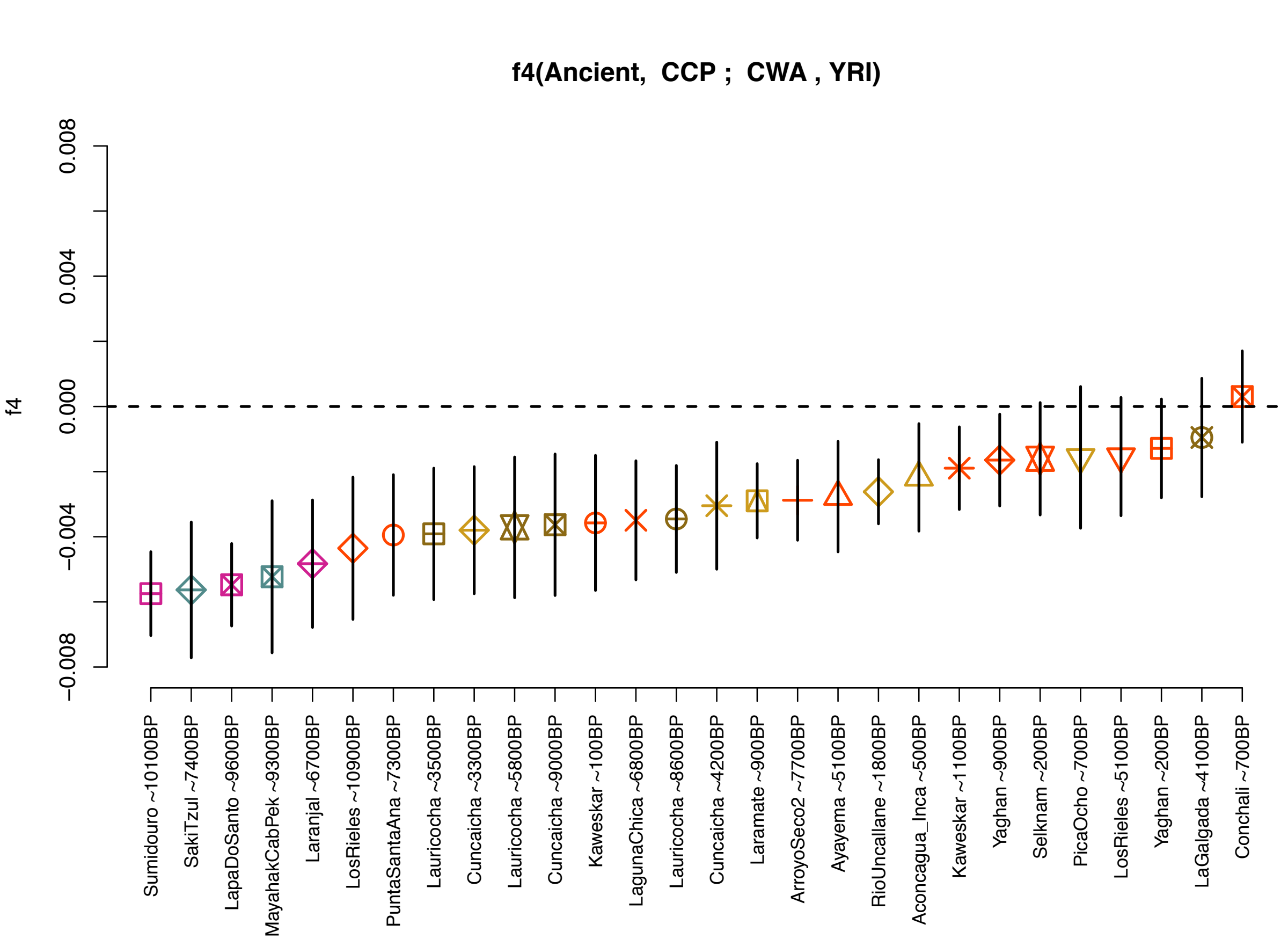

L.

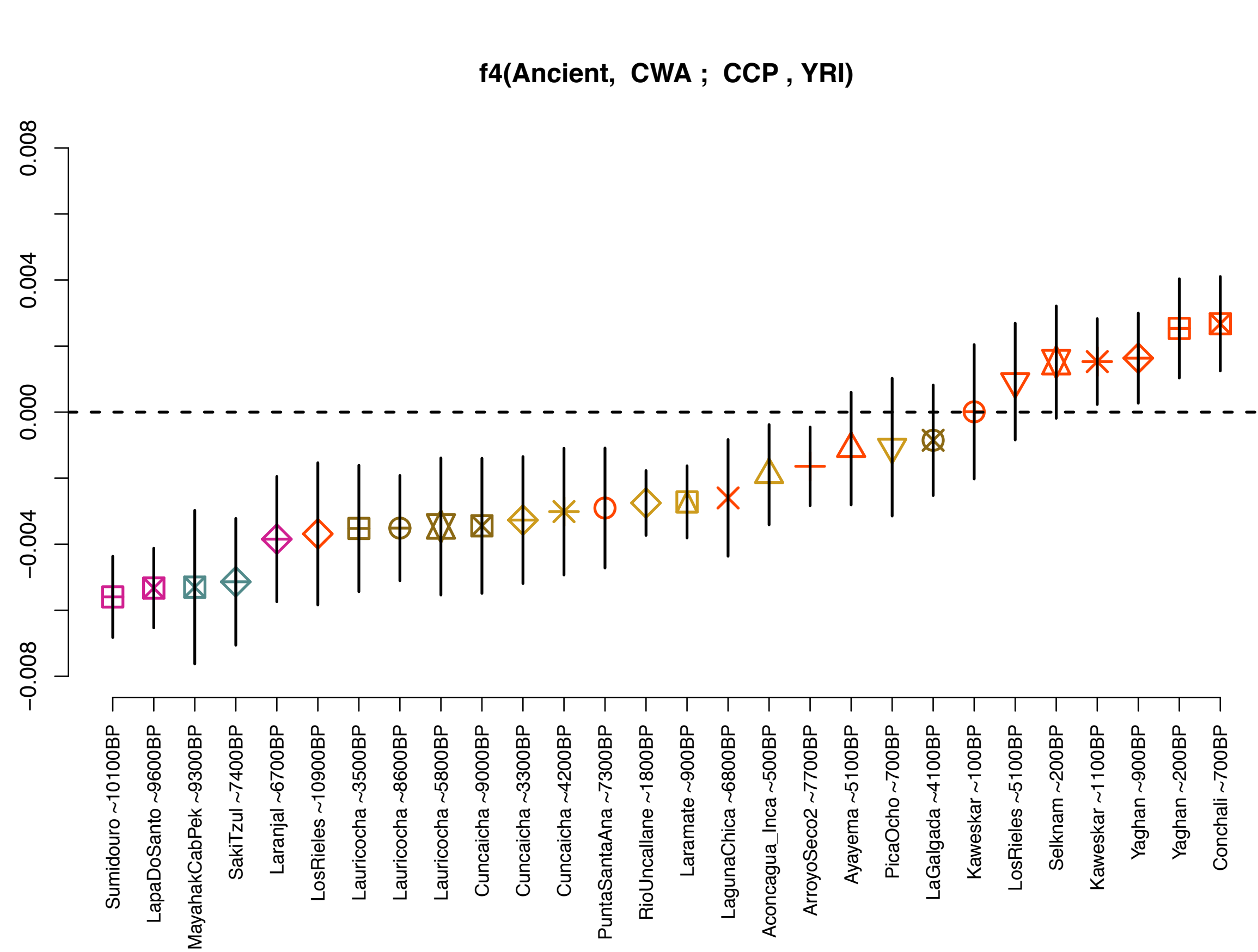

Supplement: S22 Fig — f4(Ancient, X; Y, YRI) where X and Y are two of the four identified Native American components (one plot per X-Y combination), and Ancient is ancient group labeled on the x-axis and represented with a point/color scheme as in Main Fig 5. Vertical segments are the +/- 3 standard errors intervals. CAN: Central Andes; STF: Subtropical and Tropical Forests; CCP: Central Chile / Patagonia; CWA: Central Western Argentina; YRI: Yoruba from 1KGP. (PDF) [file pone.0233808.s022.pdf]

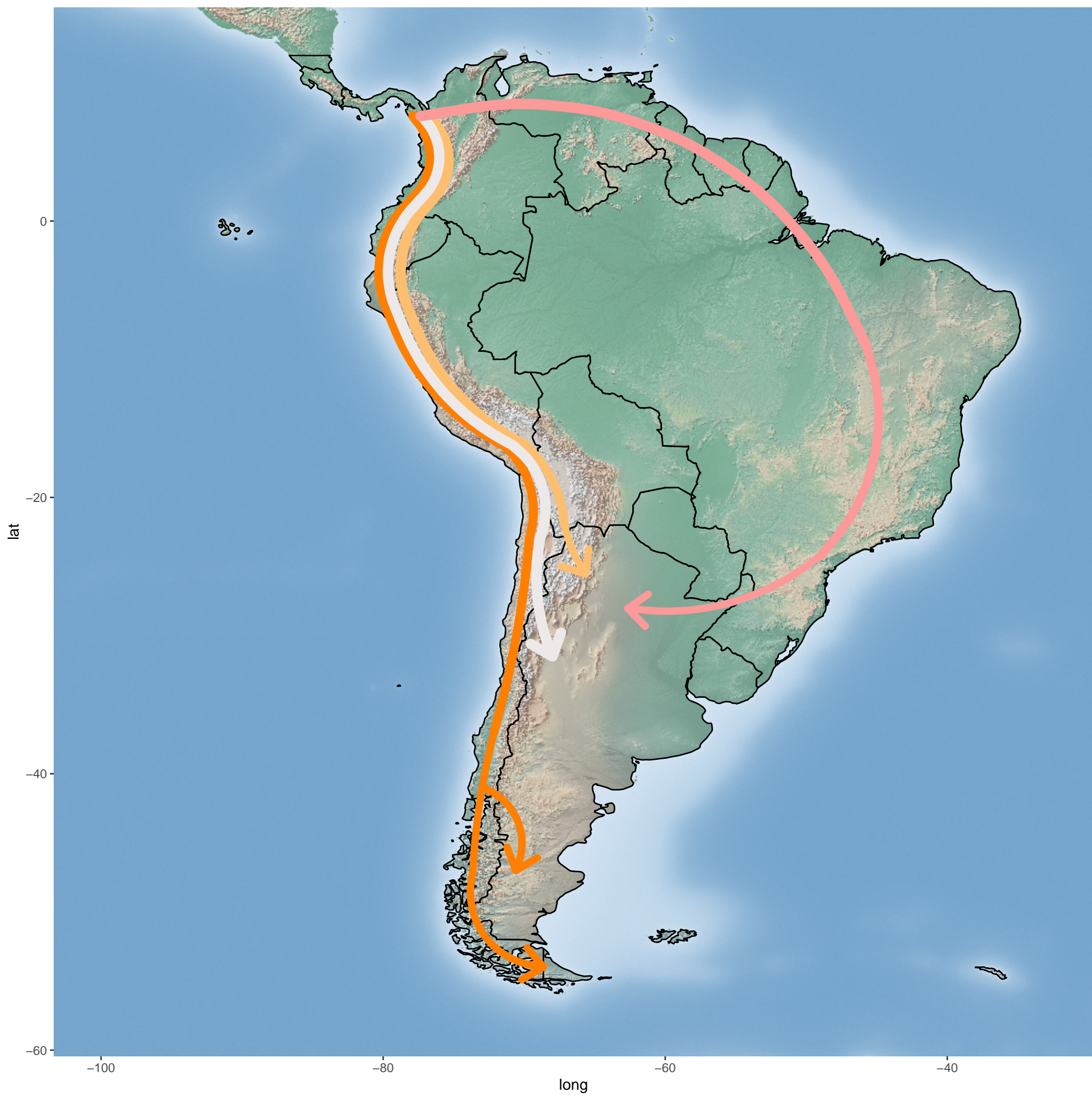

Supplement: S24 Fig — Each arrow represents one of the four components discussed throughout the article. Neither the time and place of the splits among these components nor gene flow among them have been addressed in this study. (PDF) [file pone.0233808.s024.pdf]

A.

**Cross-Validation Score**  
**Own study + de la Fuente et al. + 1KG**

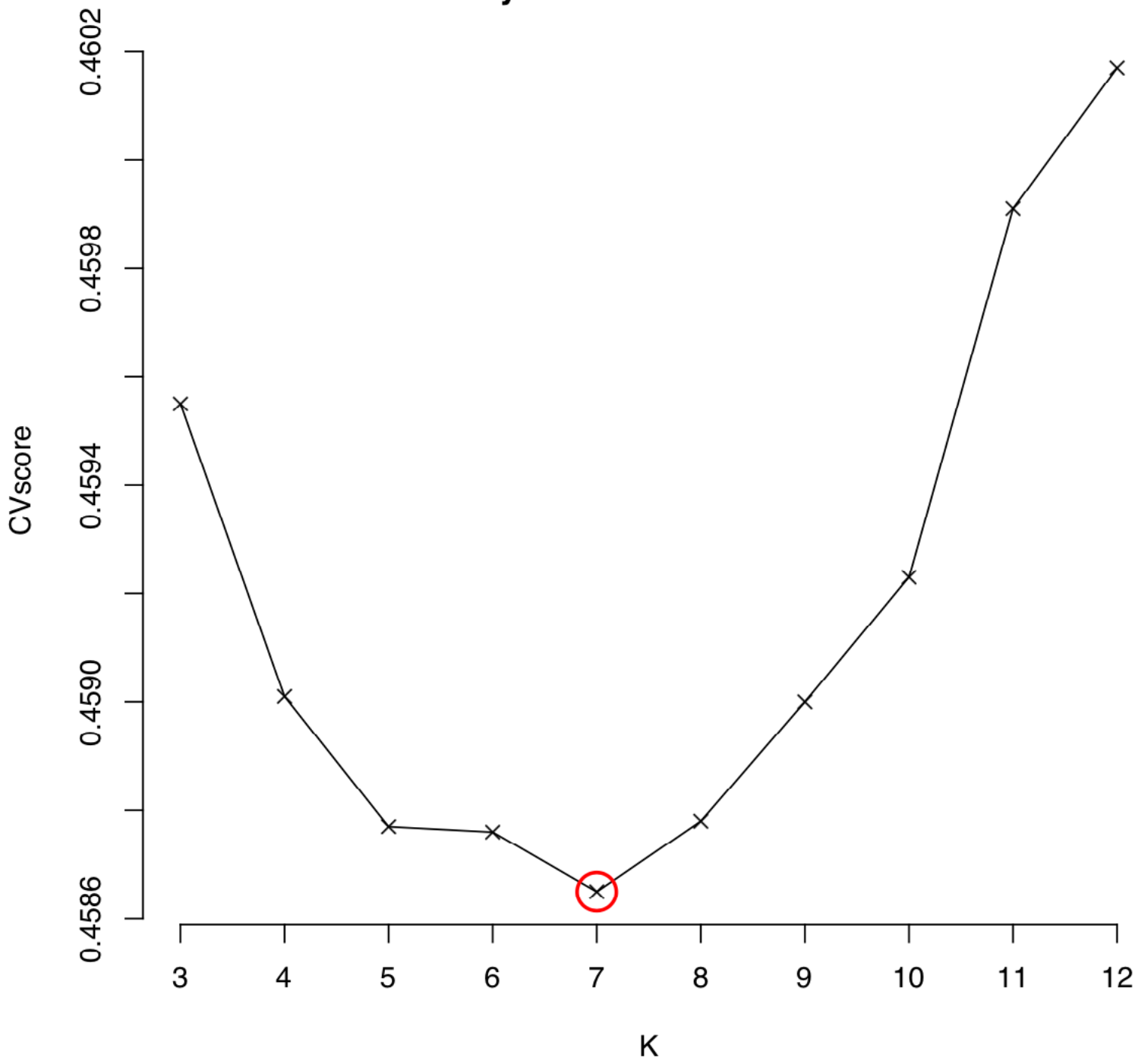

B.

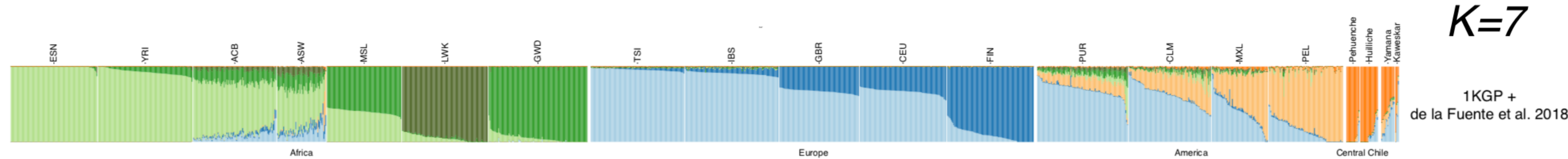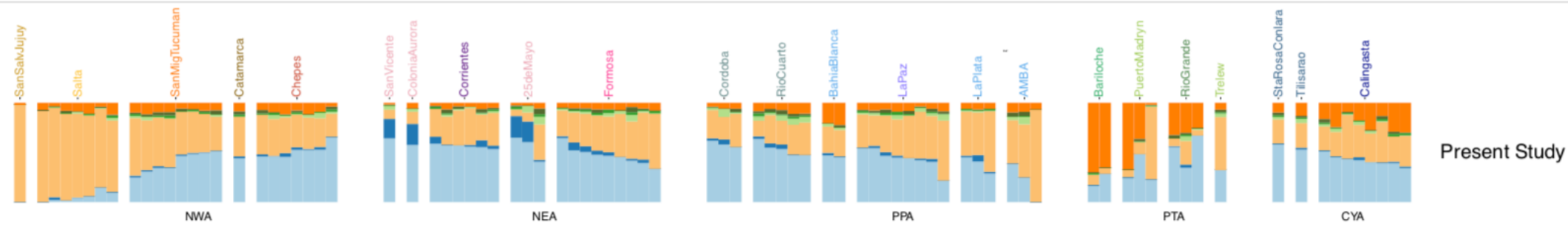

Supplement: S25 Fig — (A) Cross-validation scores from K = 3 to K = 10. (B) Admixture for K = 7. (PDF) [file pone.0233808.s025.pdf]

**A.****Cross-Validation Score  
Homburger et al. + 1KG**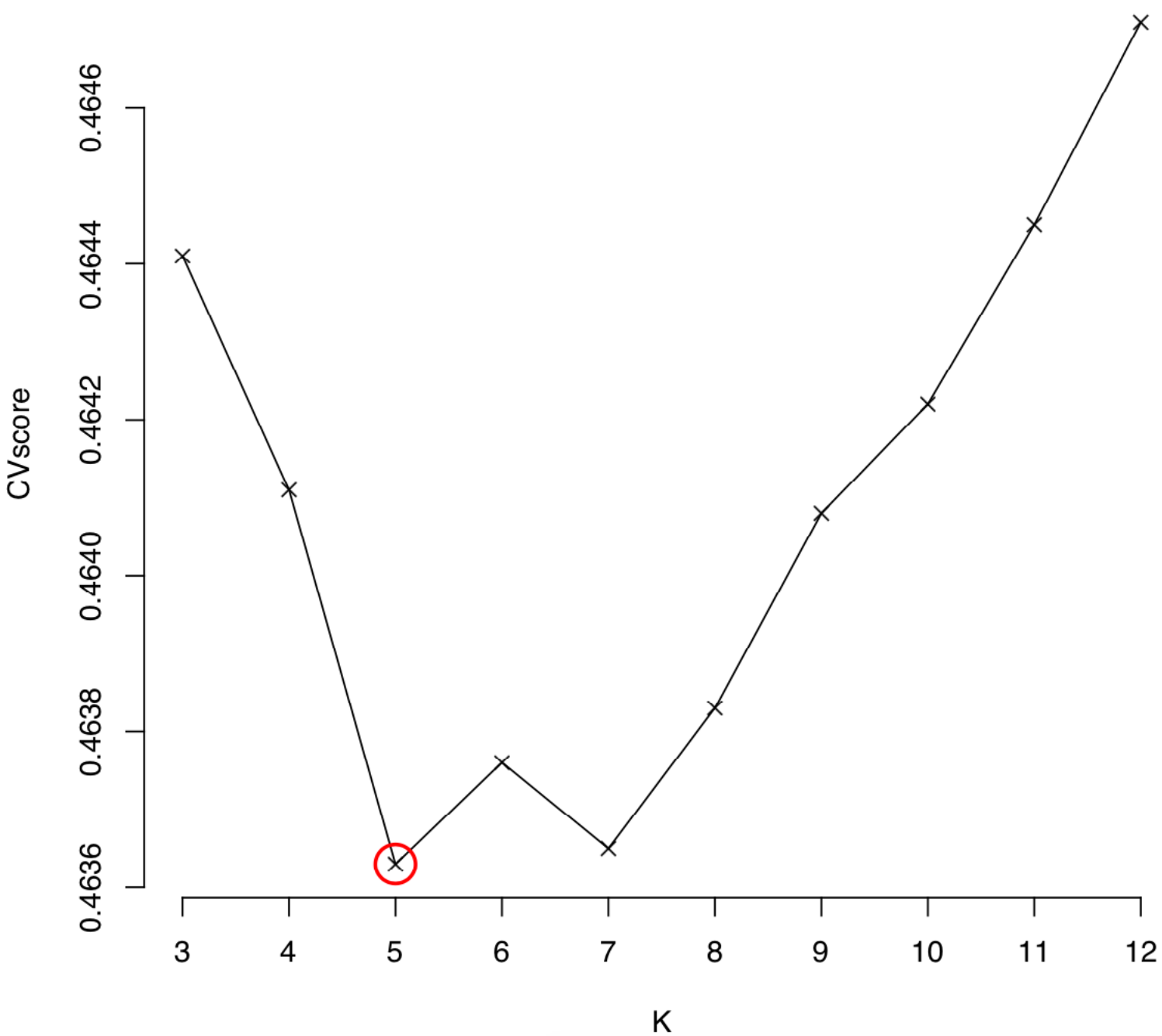

B.

$K=5$

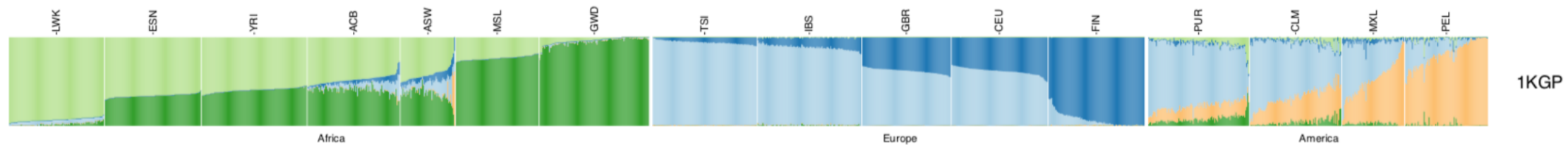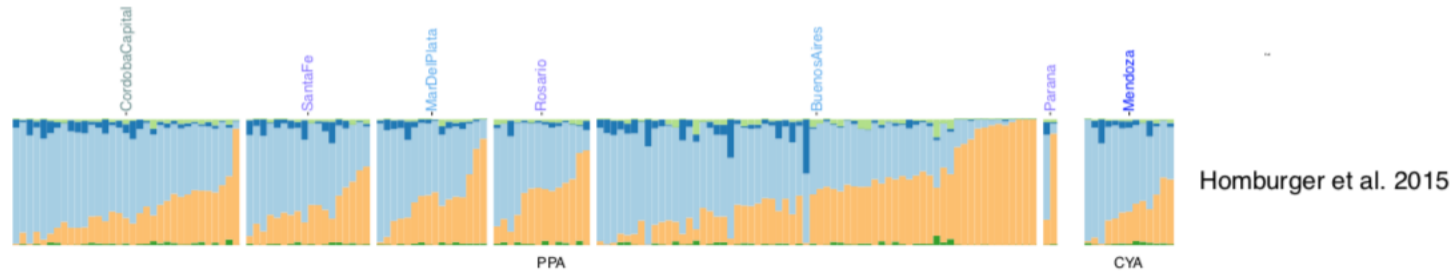

Supplement: S26 Fig — (A) Cross-validation scores from K = 3 to K = 10. (B) Admixture for K = 5. (PDF) [file pone.0233808.s026.pdf]

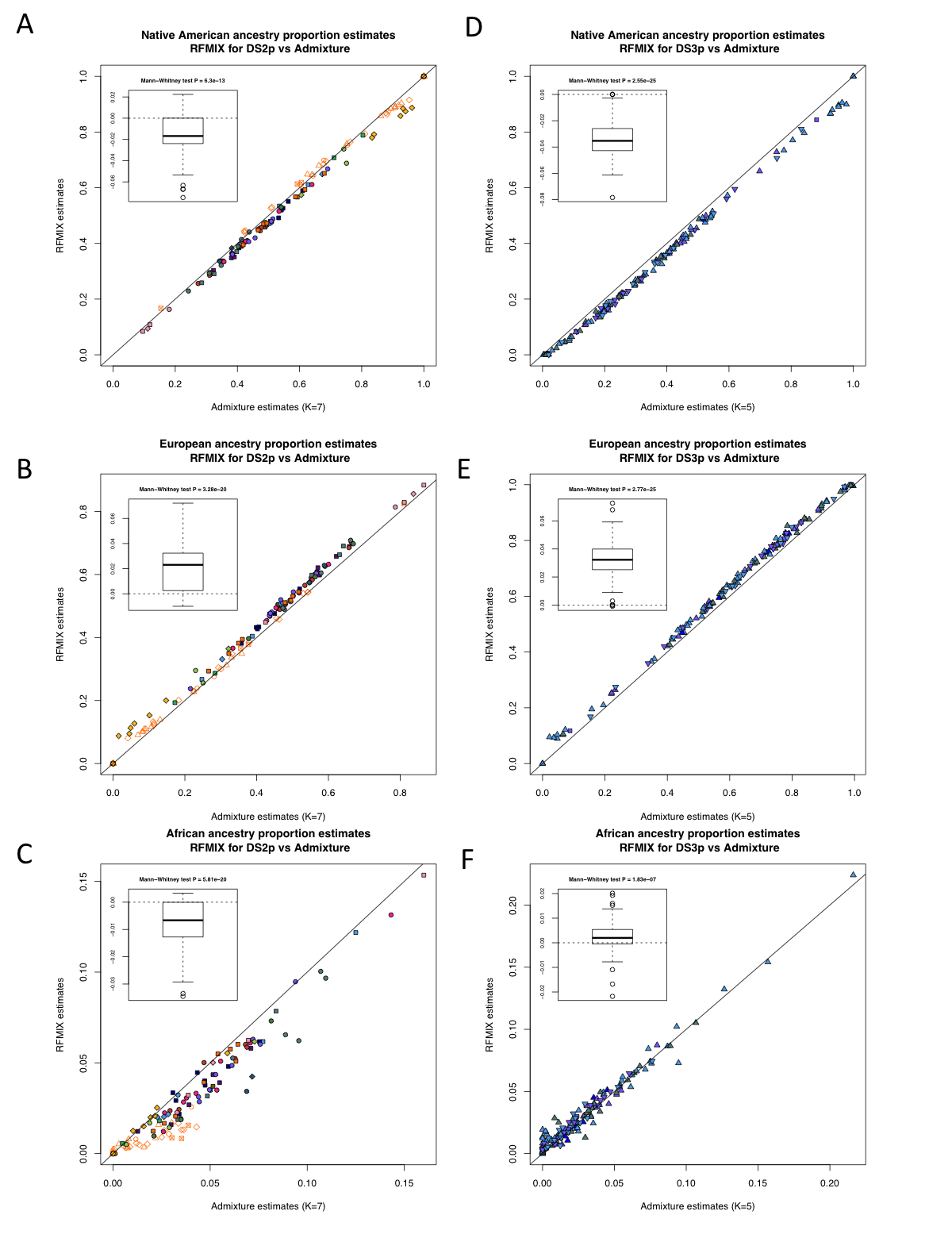

Supplement: S27 Fig — (A-C) For DS2p: Argentinean samples from the present study with reference panel that consists in 1KGP individuals from Africa, Europe and America [42] and Chilean individuals from [37]. Native American, European and African ancestry proportions estimates with RFMix vs with Admixture with K = 7. (D-F) For DS3p: Argentinean samples from [31] with reference panel that consists in 1KGP individuals from Africa, Europe and America [42]. Native American, European and African ancestry proportions estimates with RFMIx vs with Admixture with K = 5. (TIF) [file pone.0233808.s027.tif]

# % consistency Masking output in DS2p vs DS3p for 1KGP individuals

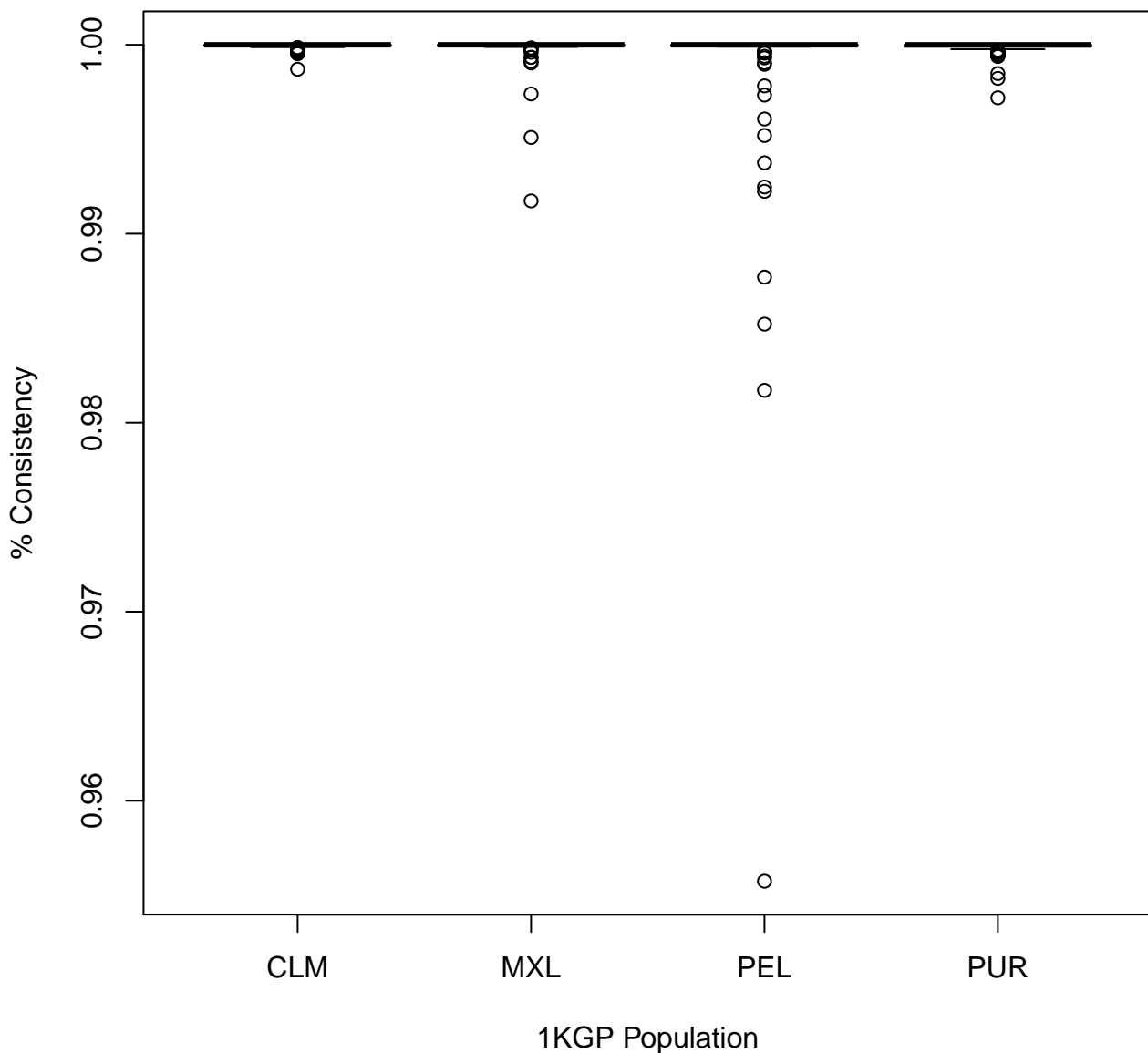

Supplement: S28 Fig — We compared the percentage of variants with same ancestry ditypes in DS2p and DS3p for American admixed individuals from the 1000 Genomes. Project. (PDF) [file pone.0233808.s028.pdf]
