# Supplementary material for: Fine-scale genomic analyses of admixed individuals reveal unrecognized genetic ancestry components in Argentina
Source: PLoS One. 2020 Jul 16;15(7):e0233808. doi: 10.1371/journal.pone.0233808 (PMC7365470; doi:10.1371/journal.pone.0233808)
Supplement: S2 Fig — (A) Cross-Validation score for Admixture runs on the worldwide meta dataset (DS1) with K from 3 to 12. (B-G) Admixture results with K = 3 to K = 8. 1KGP: 1000 Genomes Project; CYA: Cuyo Region; NEA: Northeastern Region, NWA: Northwestern Region; PPA: Pampean Region; PTA: Patagonia Region. (PDF) [file pone.0233808.s002.pdf]

A.

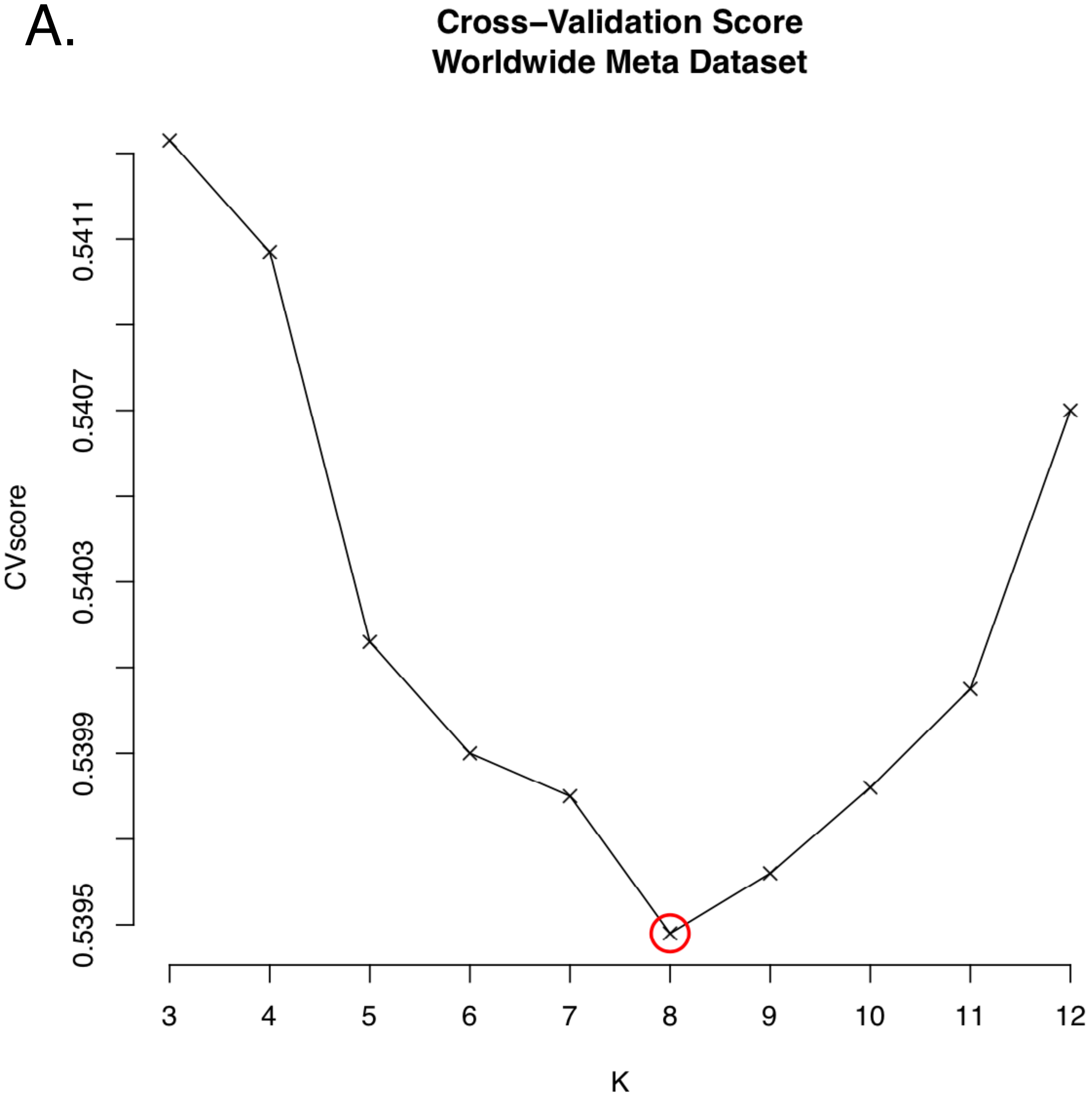
Cross−Validation Score
Worldwide Meta Dataset
CVscore
0.5395
0.5399
0.5403
0.5407
0.5411
K
3
4
5
6
7
8
9
10
11
12

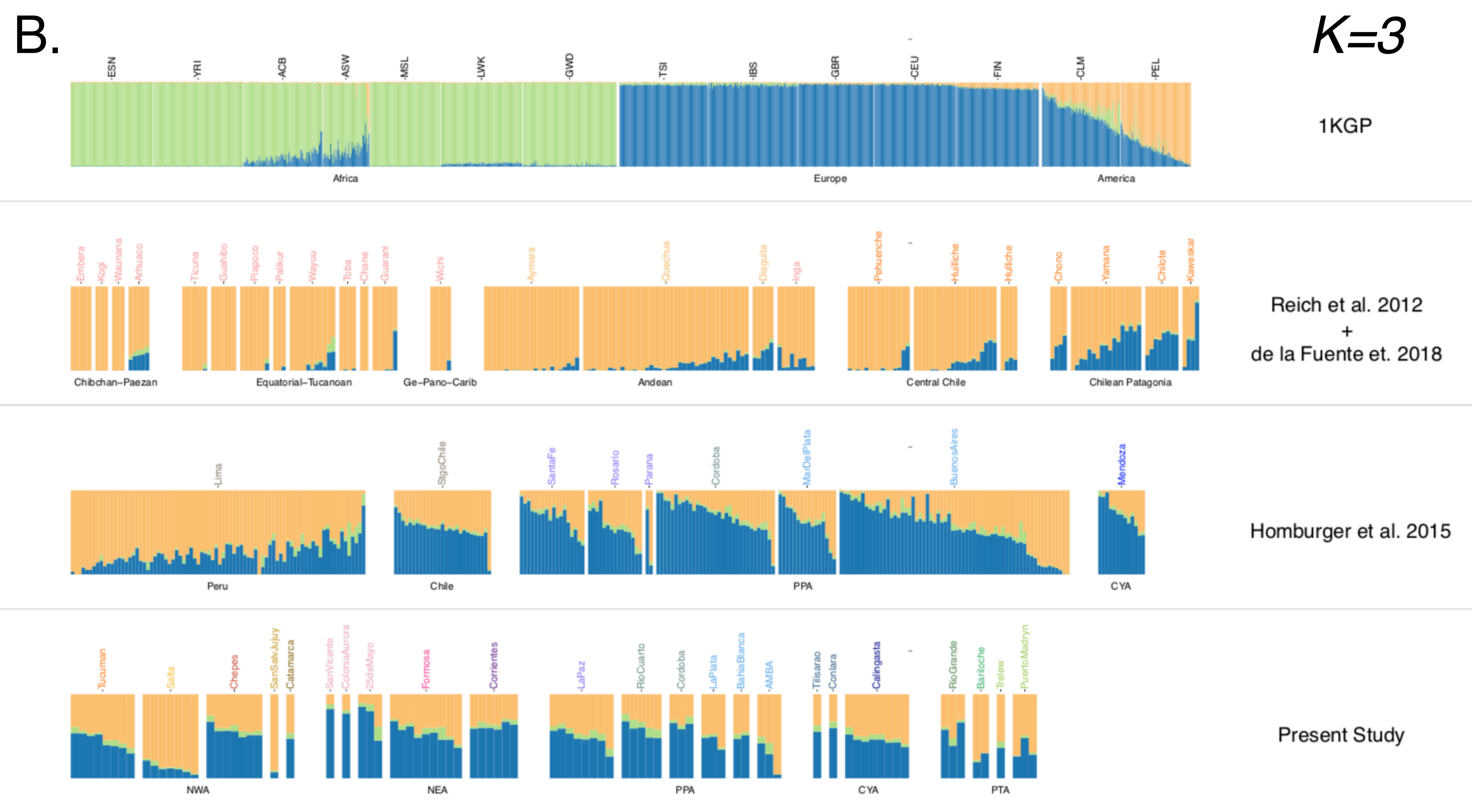

B.
K=3
ESN
YRI
ACB
ASW
MSL
LWK
GWD
TSI
IBS
GBR
CEU
FIN
CLM
PEL
Africa
Europe
America
1KGP
Embera
Kogi
Waunana
Arhuaco
Ticuna
Guahibo
Piapoco
Palikur
Wayuu
Toba
Chane
Guarani
Wichi
Aymara
Quechua
Diaguita
Inga
Pehuenche
Huilliche
Hulliche
Chono
Yamana
Chilote
Kaweskar
Chibchan–Paezan
Equatorial–Tucanoan
Ge–Pano–Carib
Andean
Central Chile
Chilean Patagonia
Reich et al. 2012
+
de la Fuente et. 2018
Lima
StgoChile
SantaFe
Rosario
Parana
Cordoba
MarDelPlata
BuenosAires
Mendoza
Peru
Chile
PPA
CYA
Homburger et al. 2015
Tucuman
Salta
Chepes
SanSalvJujuy
Catamarca
SanVicente
ColoniaAurora
25deMayo
Formosa
Corrientes
LaPaz
RioCuarto
Cordoba
LaPlata
BahiaBlanca
AMBA
Tilisarao
Conlara
Calingasta
RioGrande
Bariloche
Trelew
PuertoMadryn
NWA
NEA
PPA
CYA
PTA
Present Study

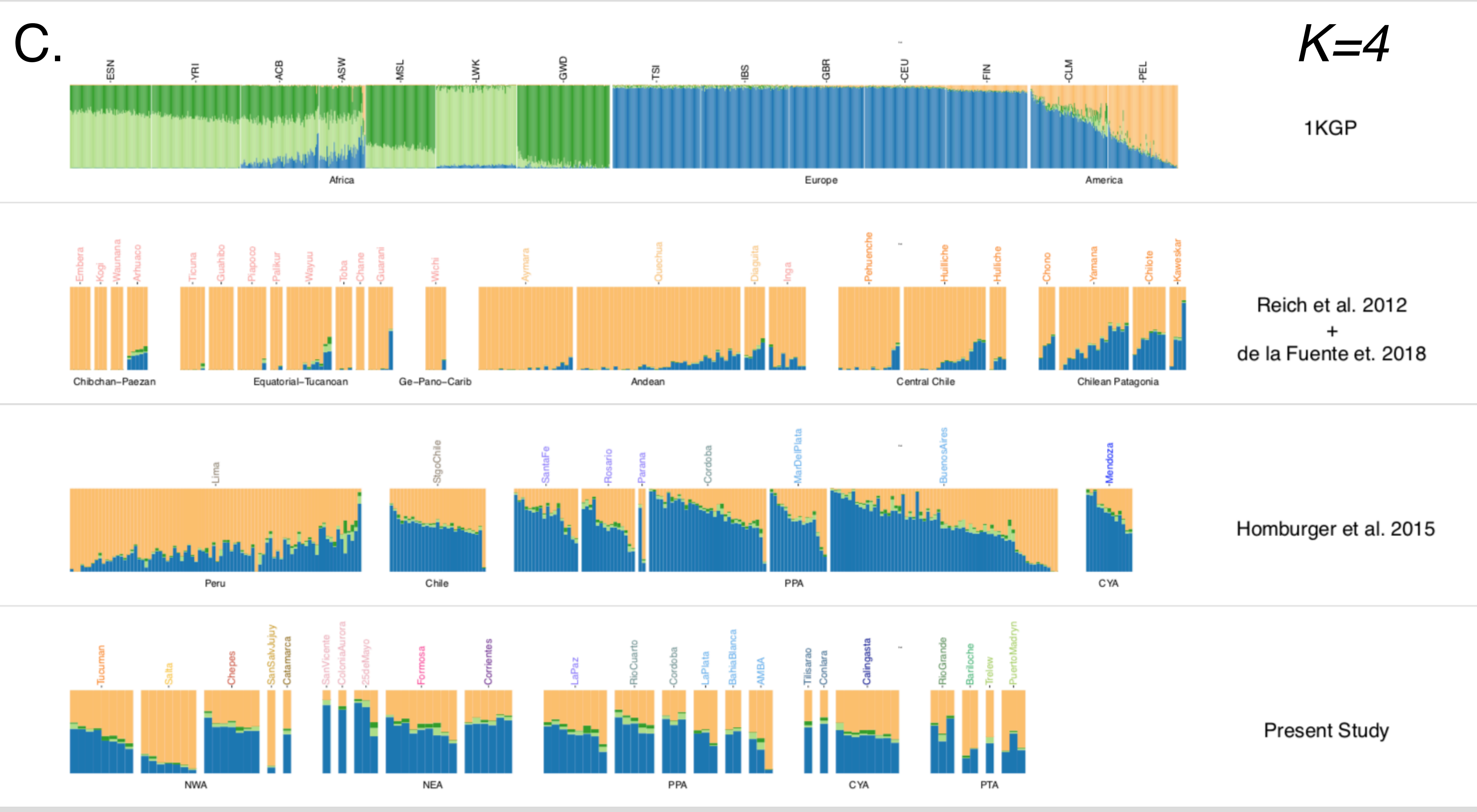

C.
K=4
ESN
YRI
ACB
ASW
MSL
LWK
GWD
TSI
IBS
GBR
CEU
FIN
CLM
PEL
Africa
Europe
America
1KGP
Embera
Kogi
Waunana
Arhuaco
Ticuna
Guahibo
Piapoco
Palikur
Wayuu
Toba
Chane
Guarani
Wichi
Aymara
Quechua
Diaguita
Inga
Pehuenche
Huilliche
Hulliche
Chono
Yamana
Chilote
Kaweskar
Chibchan–Paezan
Equatorial–Tucanoan
Ge–Pano–Carib
Andean
Central Chile
Chilean Patagonia
Reich et al. 2012
+
de la Fuente et. 2018
Lima
StgoChile
SantaFe
Rosario
Parana
Cordoba
MarDelPlata
BuenosAires
Mendoza
Peru
Chile
PPA
CYA
Homburger et al. 2015
Tucuman
Salta
Chepes
SanSalvJujuy
Catamarca
SanVicente
ColoniaAurora
25deMayo
Formosa
Corrientes
LaPaz
RioCuarto
Cordoba
LaPlata
BahiaBlanca
AMBA
Tilisarao
Conlara
Calingasta
RioGrande
Bariloche
Trelew
PuertoMadryn
NWA
NEA
PPA
CYA
PTA
Present Study

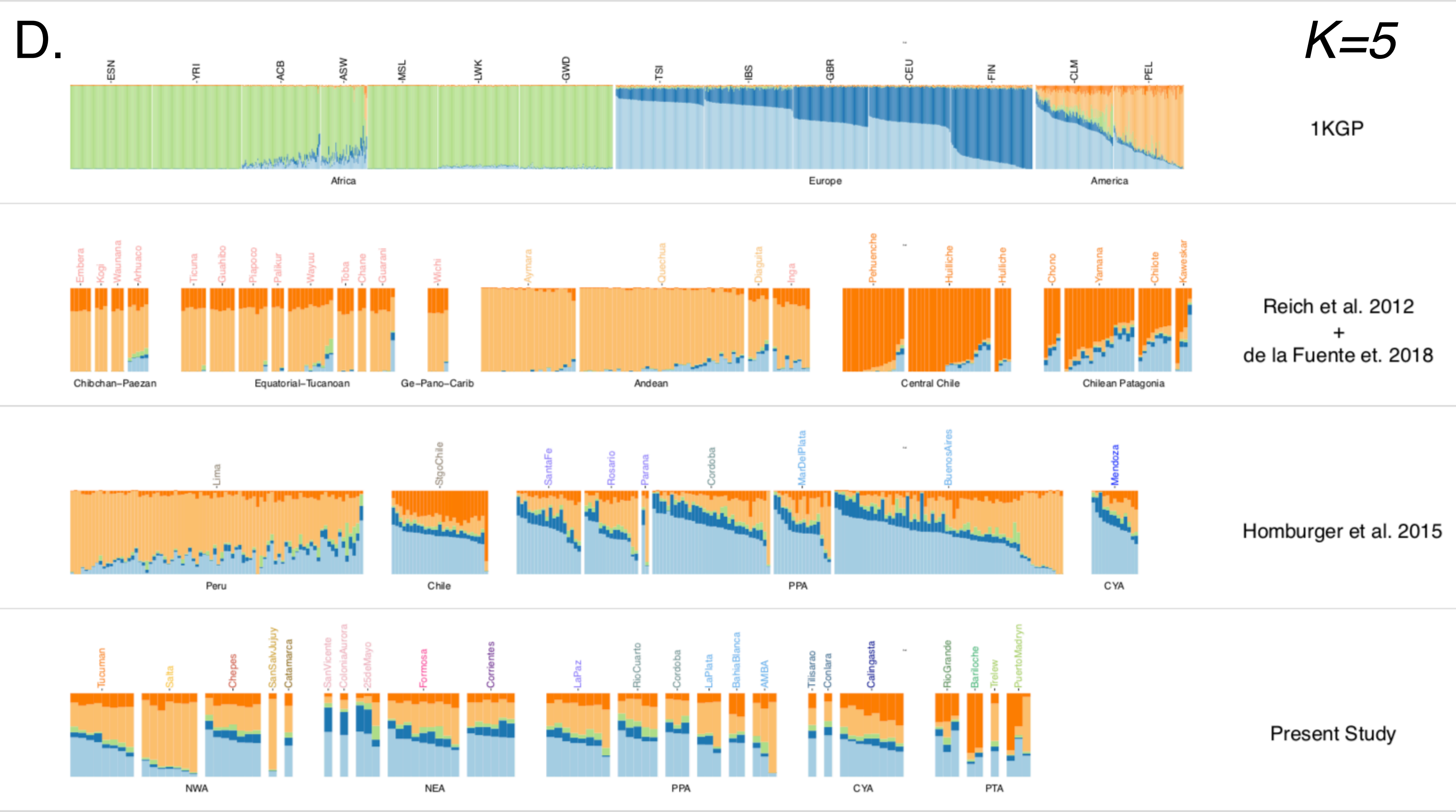

D.
K=5
ESN YRI ACB ASW MSL LWK GWD TSI IBS GBR CEU FIN CLM PEL
Africa
Europe
America
1KGP
Embera Kogi Waunana Arhuaco Ticuna Guahibo Piapoco Palikur Wayuu Toba Chane Guarani Wichi Aymara Quechua Diaguita Inga Pehuenche Huilliche Hulliche Chono Yamana Chilote Kaweskar
Chibchan–Paezan
Equatorial–Tucanoan
Ge–Pano–Carib
Andean
Central Chile
Chilean Patagonia
Reich et al. 2012
+
de la Fuente et. 2018
Lima StgoChile SantaFe Rosario Parana Cordoba MarDelPlata BuenosAires Mendoza
Peru
Chile
PPA
CYA
Homburger et al. 2015
Tucuman Salta Chepes SanSalvJujuy Catamarca SanVicente ColoniaAurora 25deMayo Formosa Corrientes LaPaz RioCuarto Cordoba LaPlata BahiaBlanca AMBA Tilisarao Conlara Calingasta RioGrande Bariloche Trelew PuertoMadryn
NWA
NEA
PPA
CYA
PTA
Present Study

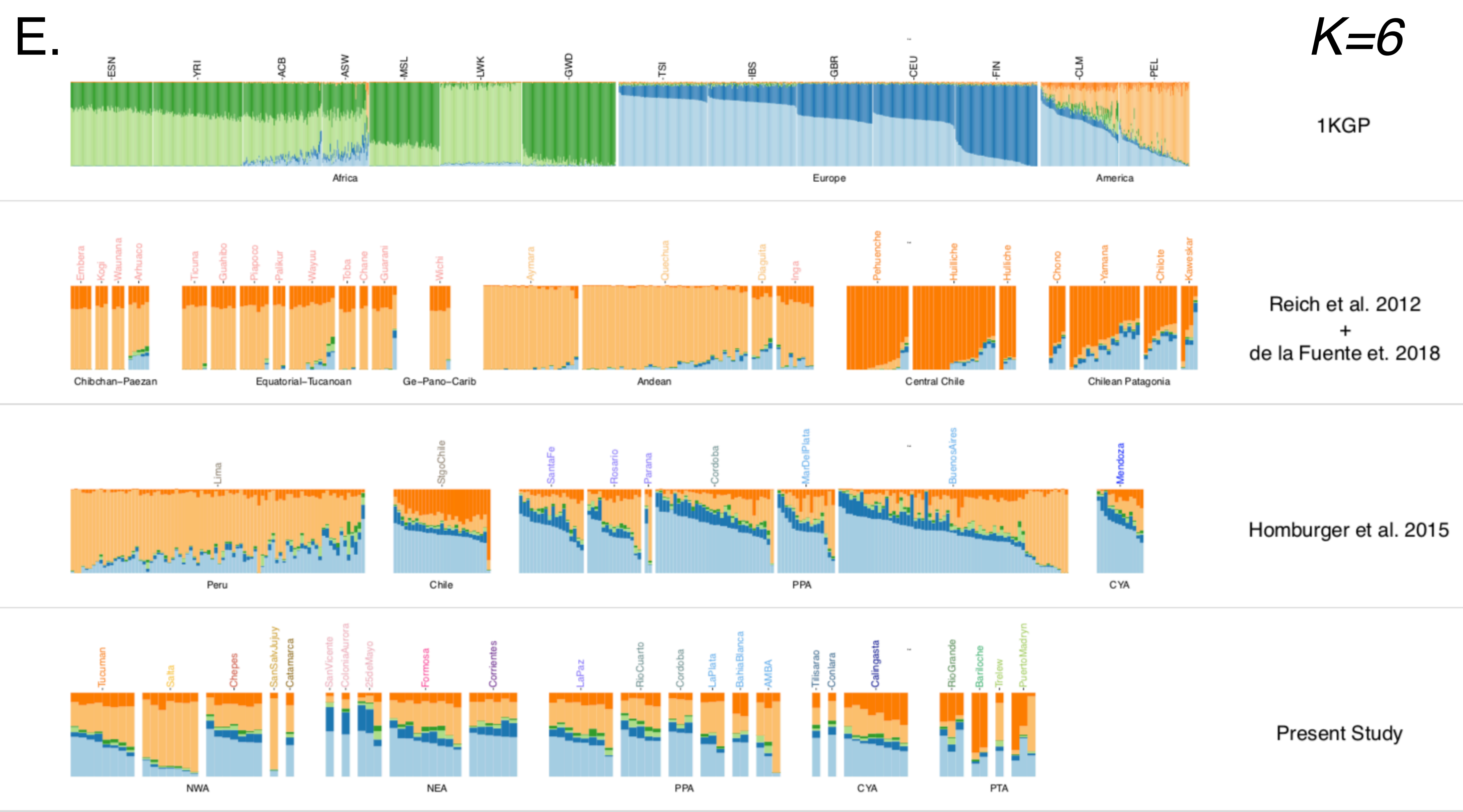
E.
K=6
ESN
YRI
ACB
ASW
MSL
LWK
GWD
TSI
IBS
GBR
CEU
FIN
CLM
PEL
Africa
Europe
America
1KGP
Embera
Kogi
Waunana
Arhuaco
Ticuna
Guahibo
Piapoco
Palikur
Wayuu
Toba
Chane
Guarani
Wichi
Aymara
Quechua
Diaguita
Inga
Pehuenche
Huilliche
Hulliche
Chono
Yamana
Chilote
Kaweskar
Chibchan–Paezan
Equatorial–Tucanoan
Ge–Pano–Carib
Andean
Central Chile
Chilean Patagonia
Reich et al. 2012
+
de la Fuente et. 2018
Lima
StgoChile
SantaFe
Rosario
Parana
Cordoba
MarDelPlata
BuenosAires
Mendoza
Peru
Chile
PPA
CYA
Homburger et al. 2015
Tucuman
Salta
Chepes
SanSalvJujuy
Catamarca
SanVicente
ColoniaAurora
25deMayo
Formosa
Corrientes
LaPaz
RioCuarto
Cordoba
LaPlata
BahiaBlanca
AMBA
Tilisarao
Conlara
Calingasta
RioGrande
Bariloche
Trelew
PuertoMadryn
NWA
NEA
PPA
CYA
PTA
Present Study

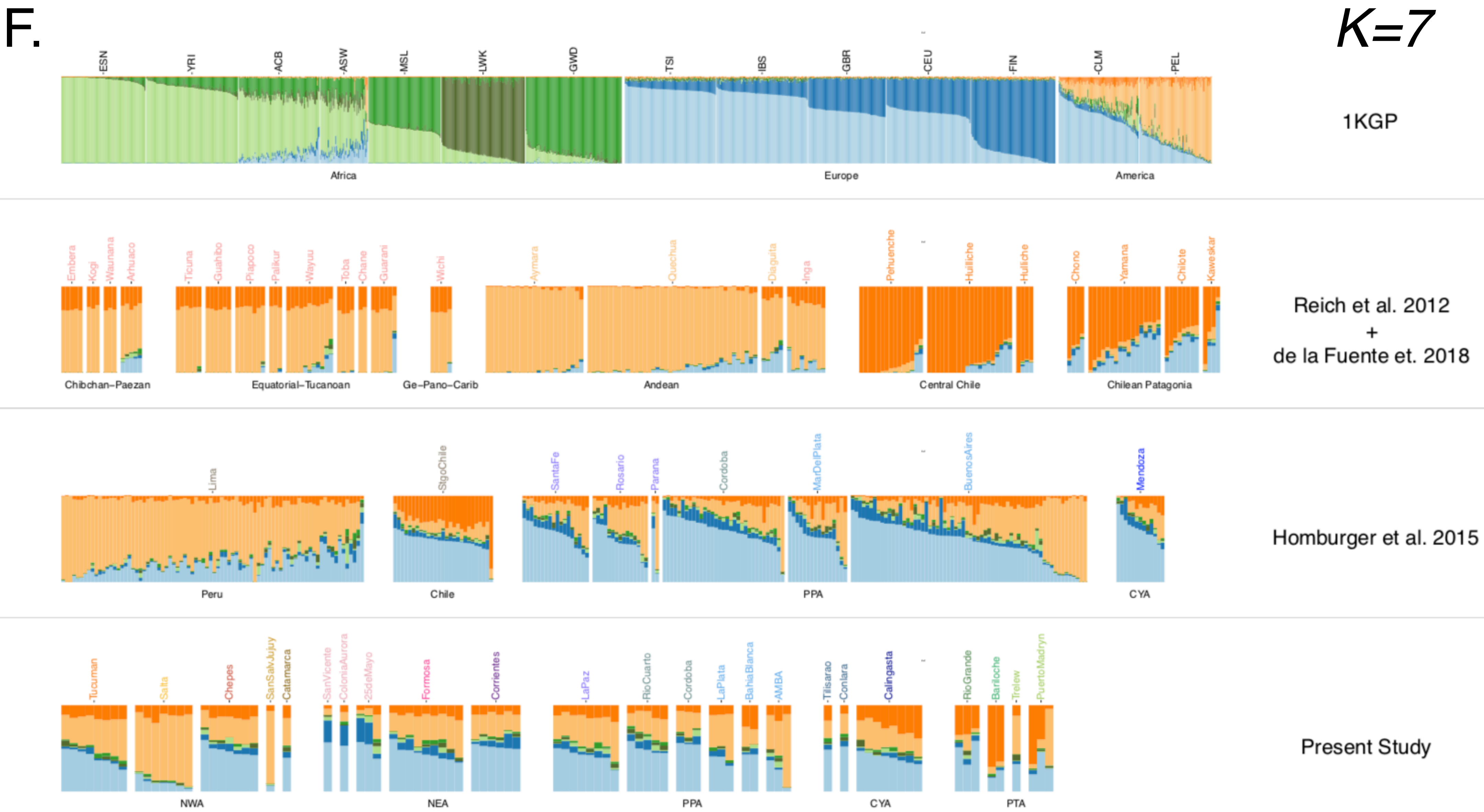

F.
K=7
ESN
YRI
ACB
ASW
MSL
LWK
GWD
TSI
IBS
GBR
CEU
FIN
CLM
PEL
Africa
Europe
America
1KGP
Embera
Kogi
Waunana
Arhuaco
Ticuna
Guahibo
Piapoco
Palikur
Wayuu
Toba
Chane
Guarani
Wichi
Aymara
Quechua
Diaguita
Inga
Pehuenche
Huilliche
Huilliche
Chono
Yamana
Chilote
Kaweskar
Chibchan–Paezan
Equatorial–Tucanoan
Ge–Pano–Carib
Andean
Central Chile
Chilean Patagonia
Reich et al. 2012
+
de la Fuente et. 2018
Lima
StgoChile
SantaFe
Rosario
Parana
Cordoba
MarDelPlata
BuenosAires
Mendoza
Peru
Chile
PPA
CYA
Homburger et al. 2015
Tucuman
Salta
Chepes
SanSalvJujuy
Catamarca
SanVicente
ColoniaAurora
25deMayo
Formosa
Corrientes
LaPaz
RioCuarto
Cordoba
LaPlata
BahiaBlanca
AMBA
Tilisarao
Conlara
Calingasta
RioGrande
Bariloche
Trelew
PuertoMadryn
NWA
NEA
PPA
CYA
PTA
Present Study

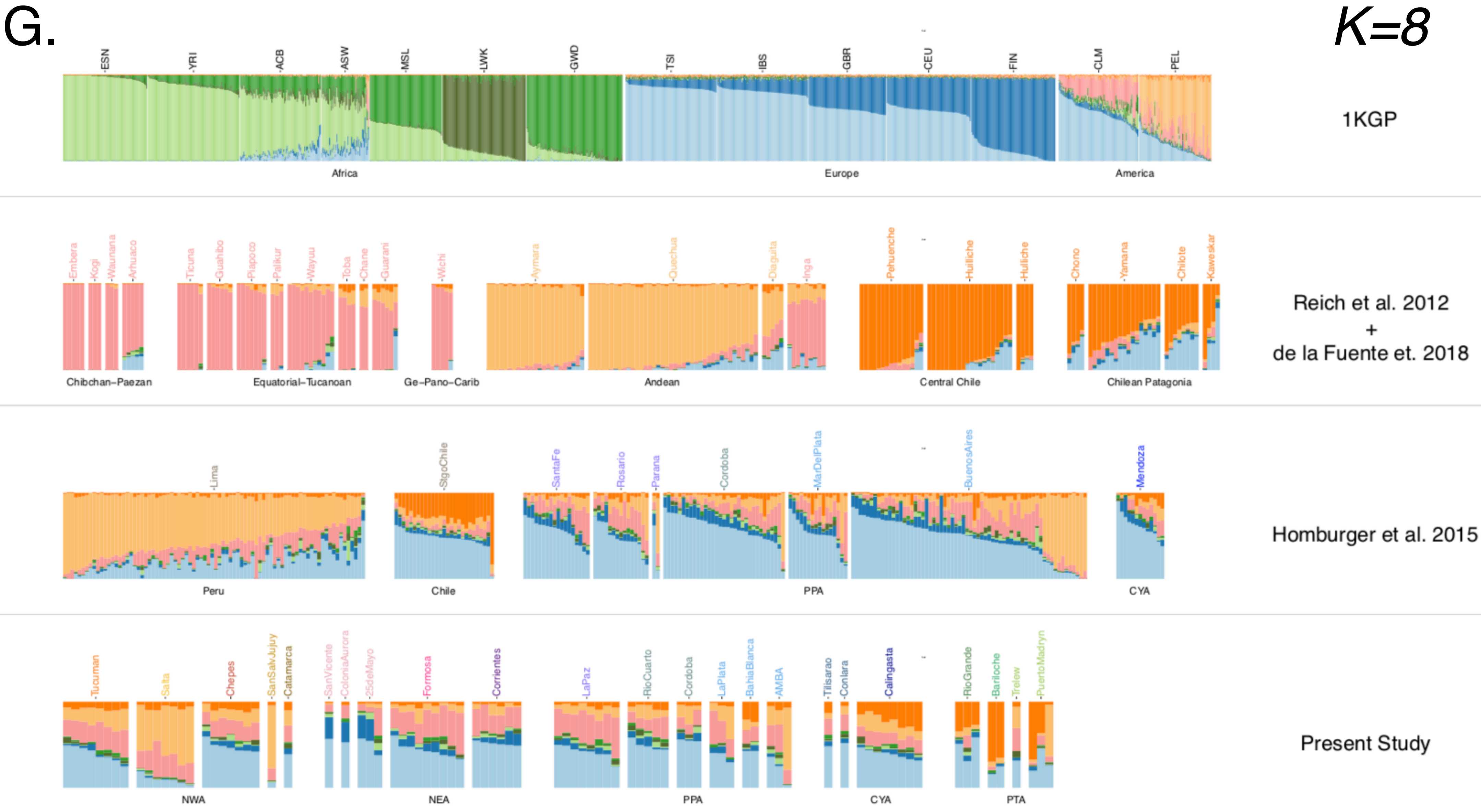
G.
K=8
ESN
YRI
ACB
ASW
MSL
LWK
GWD
TSI
IBS
GBR
CEU
FIN
CLM
PEL
Africa
Europe
America
1KGP
Embera
Kogi
Waunana
Arhuaco
Ticuna
Guahibo
Piapoco
Palikur
Wayuu
Toba
Chane
Guarani
Wichi
Aymara
Quechua
Diaguita
Inga
Pehuenche
Huilliche
Huilliche
Chono
Yamana
Chilote
Kaweskar
Chibchan-Paezan
Equatorial-Tucanoan
Ge-Pano-Carib
Andean
Central Chile
Chilean Patagonia
Reich et al. 2012
+
de la Fuente et. 2018
Lima
StgoChile
SantaFe
Rosario
Parana
Cordoba
MarDelPlata
BuenosAires
Mendoza
Peru
Chile
PPA
CYA
Homburger et al. 2015
Tucuman
Salta
Chepes
SanSalvJujuy
Catamarca
SanVicente
ColoniaAurora
25deMayo
Formosa
Corrientes
LaPaz
RioCuarto
Cordoba
LaPlata
BahiaBlanca
AMBA
Tilisarao
Conlara
Calingasta
RioGrande
Bariloche
Trelew
PuertoMadryn
NWA
NEA
PPA
CYA
PTA
Present Study
