# Supplementary material for: Fine-scale genomic analyses of admixed individuals reveal unrecognized genetic ancestry components in Argentina
Source: PLoS One. 2020 Jul 16;15(7):e0233808. doi: 10.1371/journal.pone.0233808 (PMC7365470; doi:10.1371/journal.pone.0233808)
Supplement: S7 Fig — (A) Cross-Validation scores for K from 2 to 10. (B) Admixture for K = 2. (C) Admixture for K = 3. CYA: Cuyo Region; NEA: Northeastern Region, NWA: Northwestern Region; PPA: Pampean Region; PTA: Patagonia Region. (PDF) [file pone.0233808.s007.pdf]

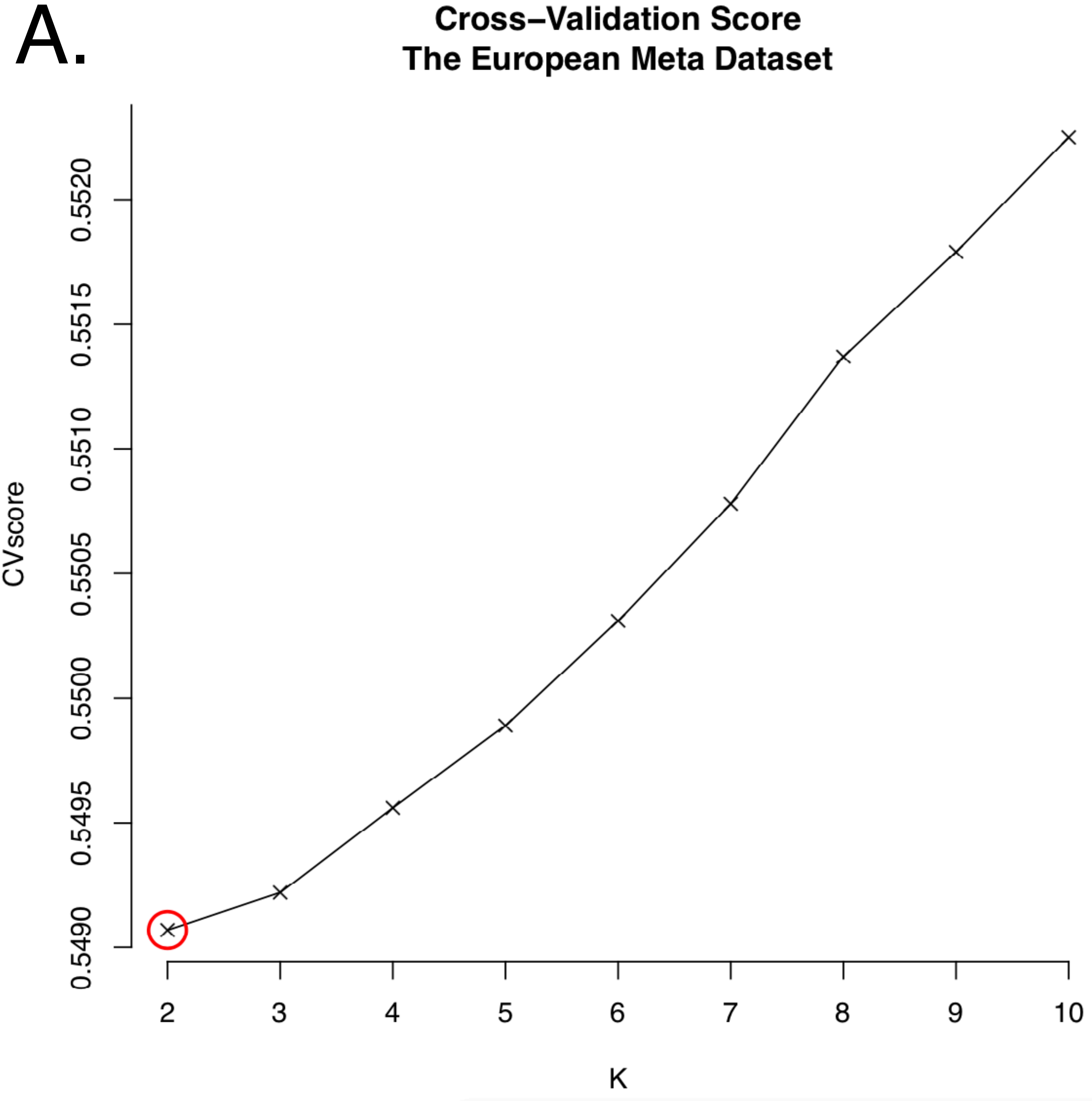
A.
Cross–Validation Score
The European Meta Dataset
CVscore
0.5490
0.5495
0.5500
0.5505
0.5510
0.5515
0.5520
K
2
3
4
5
6
7
8
9
10

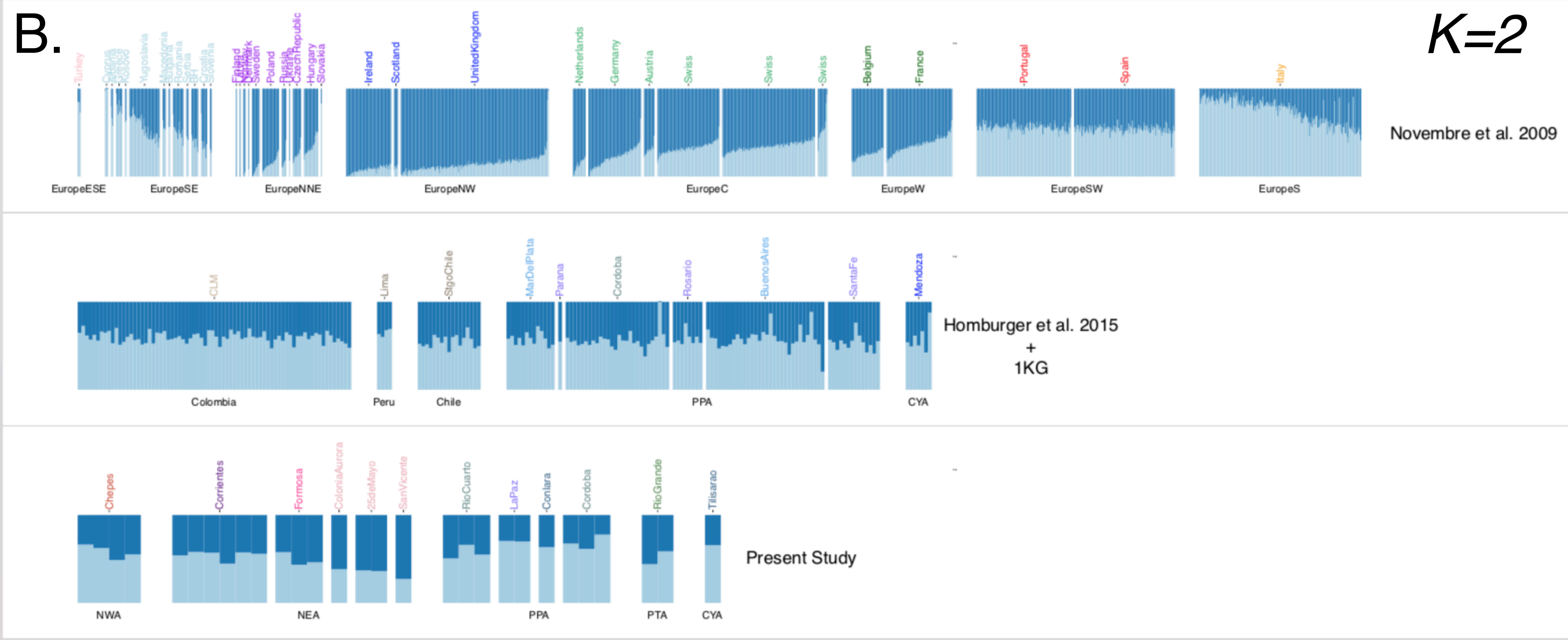
B.
K=2
Turkey
Yugoslavia
Finland
Sweden
Poland
Russia
Czech Republic
Hungary
Slovakia
Ireland
Scotland
UnitedKingdom
Netherlands
Germany
Austria
Swiss
Swiss
Swiss
Belgium
France
Portugal
Spain
Italy
EuropeESE
EuropeSE
EuropeNNE
EuropeNW
EuropeC
EuropeW
EuropeSW
EuropeS
Novembre et al. 2009
CLM
Lima
StgoChile
MarDelPlata
Parana
Cordoba
Rosario
BuenosAires
SantaFe
Mendoza
Colombia
Peru
Chile
PPA
CYA
Homburger et al. 2015
+
1KG
Chepes
Corrientes
Formosa
ColoniaAurora
25deMayo
SanVicente
RioCuarto
LaPaz
Conlara
Cordoba
RioGrande
Tilisarao
NWA
NEA
PPA
PTA
CYA
Present Study

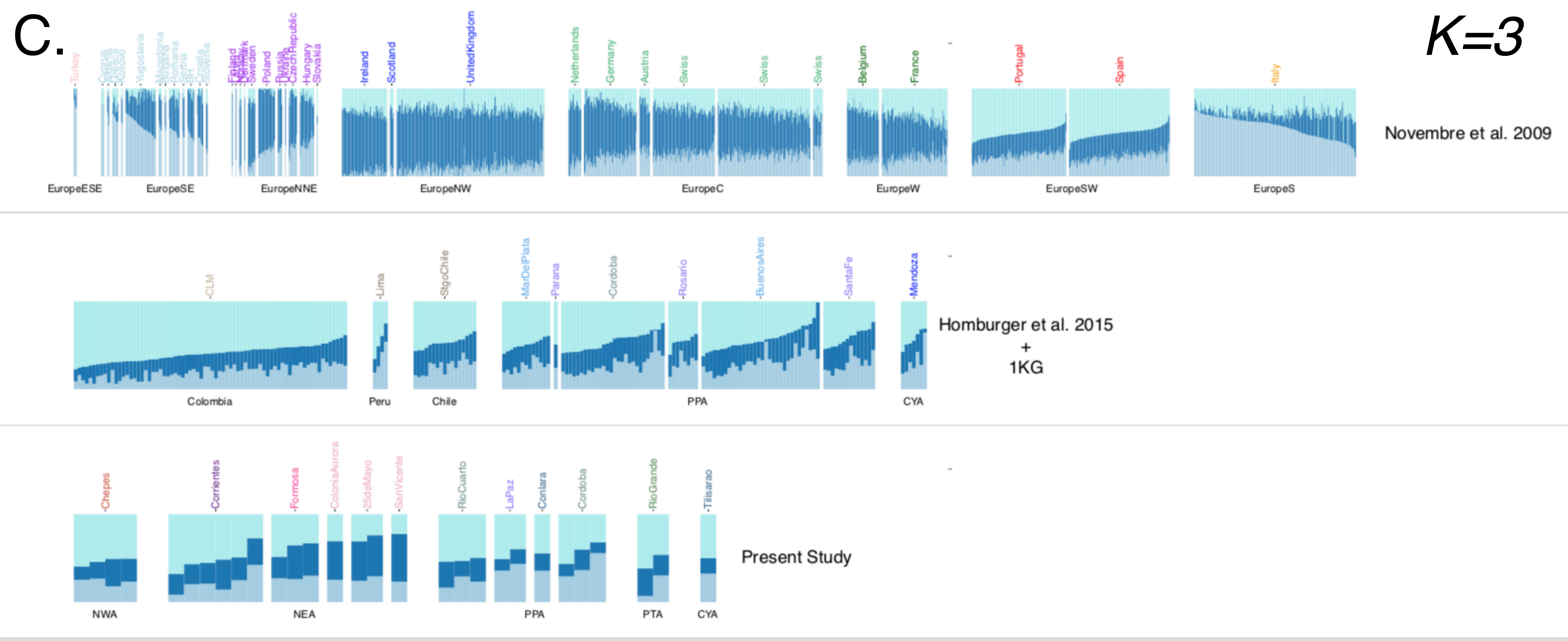
C.
K=3
Turkey
Cyprus
Greece
Kosovo
Yugoslavia
Macedonia
Bulgaria
Romania
Serbia
BH
Croatia
Slovenia
Finland
Norway
Denmark
Sweden
Poland
Russia
Ukraine
CzechRepublic
Hungary
Slovakia
Ireland
Scotland
UnitedKingdom
Netherlands
Germany
Austria
Swiss
Swiss
Swiss
Belgium
France
Portugal
Spain
Italy
Novembre et al. 2009
EuropeESE
EuropeSE
EuropeNNE
EuropeNW
EuropeC
EuropeW
EuropeSW
EuropeS
CLM
Lima
StgoChile
MarDelPlata
Parana
Cordoba
Rosario
BuenosAires
SantaFe
Mendoza
Homburger et al. 2015
+
1KG
Colombia
Peru
Chile
PPA
CYA
Chepes
Corrientes
Formosa
ColoniaAurora
25deMayo
SanVicente
RioCuarto
LaPaz
Conlara
Cordoba
RioGrande
Tilisarao
Present Study
NWA
NEA
PPA
PTA
CYA
