# Supplementary material for: Fine-scale genomic analyses of admixed individuals reveal unrecognized genetic ancestry components in Argentina
Source: PLoS One. 2020 Jul 16;15(7):e0233808. doi: 10.1371/journal.pone.0233808 (PMC7365470; doi:10.1371/journal.pone.0233808)
Supplement: S8 Fig — Comparison of ancestry proportion estimates from European Ancestry Specific Admixture Analyses (K = 3) among samples from the Argentina, Chile and Colombia (A) Southeaster/Italian ancestry (light blue in S7C Fig). (B) Iberian ancestry (turquoise in S7C Fig). (C) Northern Ancestry (dark blue in S7C Fig). P-value of the Wilcoxon test for each pairwise comparison is shown. (PDF) [file pone.0233808.s008.pdf]

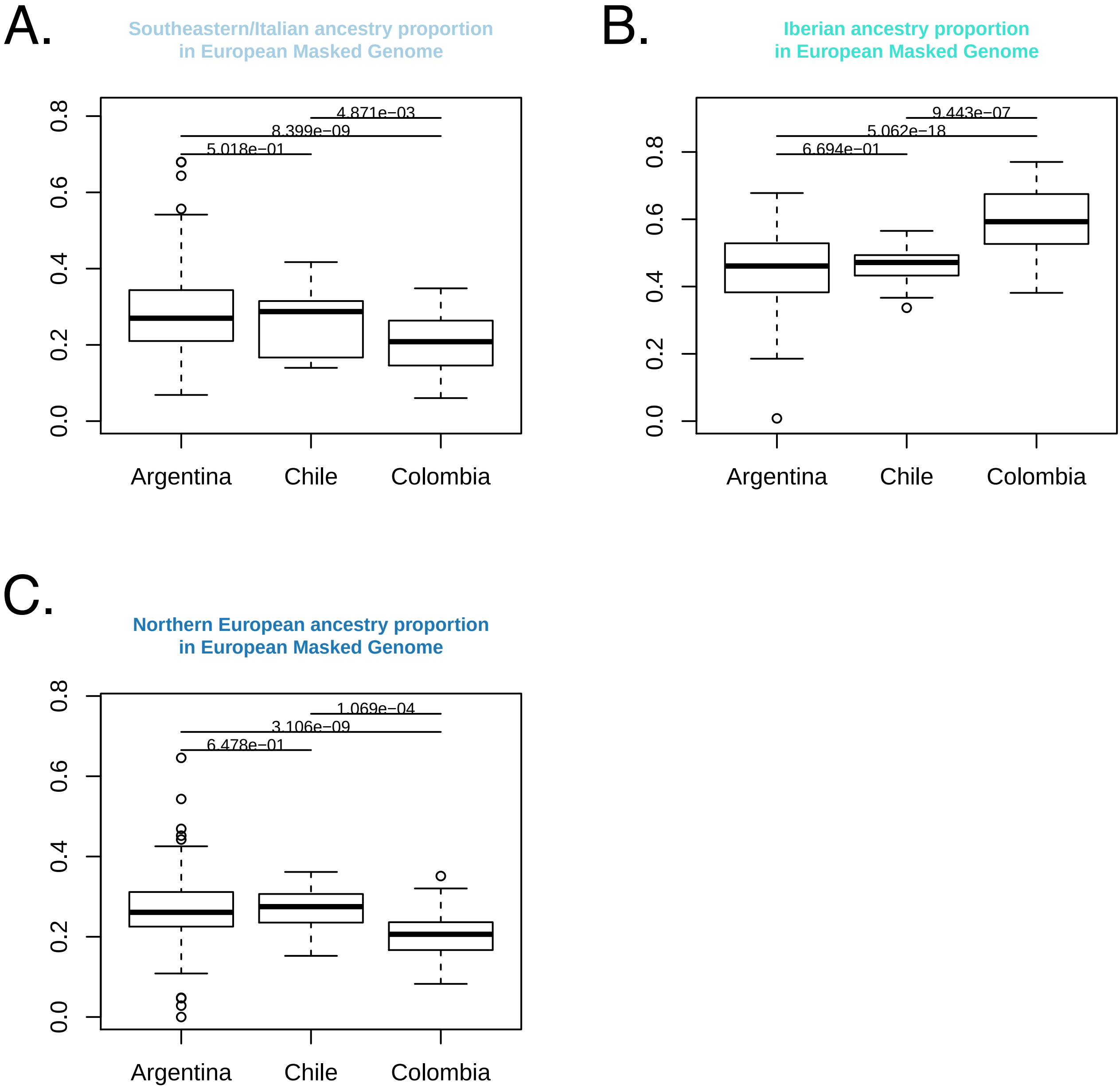

A.
Southeastern/Italian ancestry proportion
in European Masked Genome
4.871e−03
8.399e−09
5.018e−01
0.0
0.2
0.4
0.6
0.8
Argentina
Chile
Colombia
B.
Iberian ancestry proportion
in European Masked Genome
9.443e−07
5.062e−18
6.694e−01
0.0
0.2
0.4
0.6
0.8
Argentina
Chile
Colombia
C.
Northern European ancestry proportion
in European Masked Genome
1.069e−04
3.106e−09
6.478e−01
0.0
0.2
0.4
0.6
0.8
Argentina
Chile
Colombia
