# Supplementary material for: Fine-scale genomic analyses of admixed individuals reveal unrecognized genetic ancestry components in Argentina
Source: PLoS One. 2020 Jul 16;15(7):e0233808. doi: 10.1371/journal.pone.0233808 (PMC7365470; doi:10.1371/journal.pone.0233808)
Supplement: S10 Fig — (A) Cross-validation scores K from 2 to 10. (B) Admixture for K = 2. (C) Admixture for K = 3. (D) Admixture plots for K = 4. CYA: Cuyo Region; NEA: Northeastern Region, NWA: Northwestern Region; PPA: Pampean Region; PTA: Patagonia Region. (PDF) [file pone.0233808.s010.pdf]

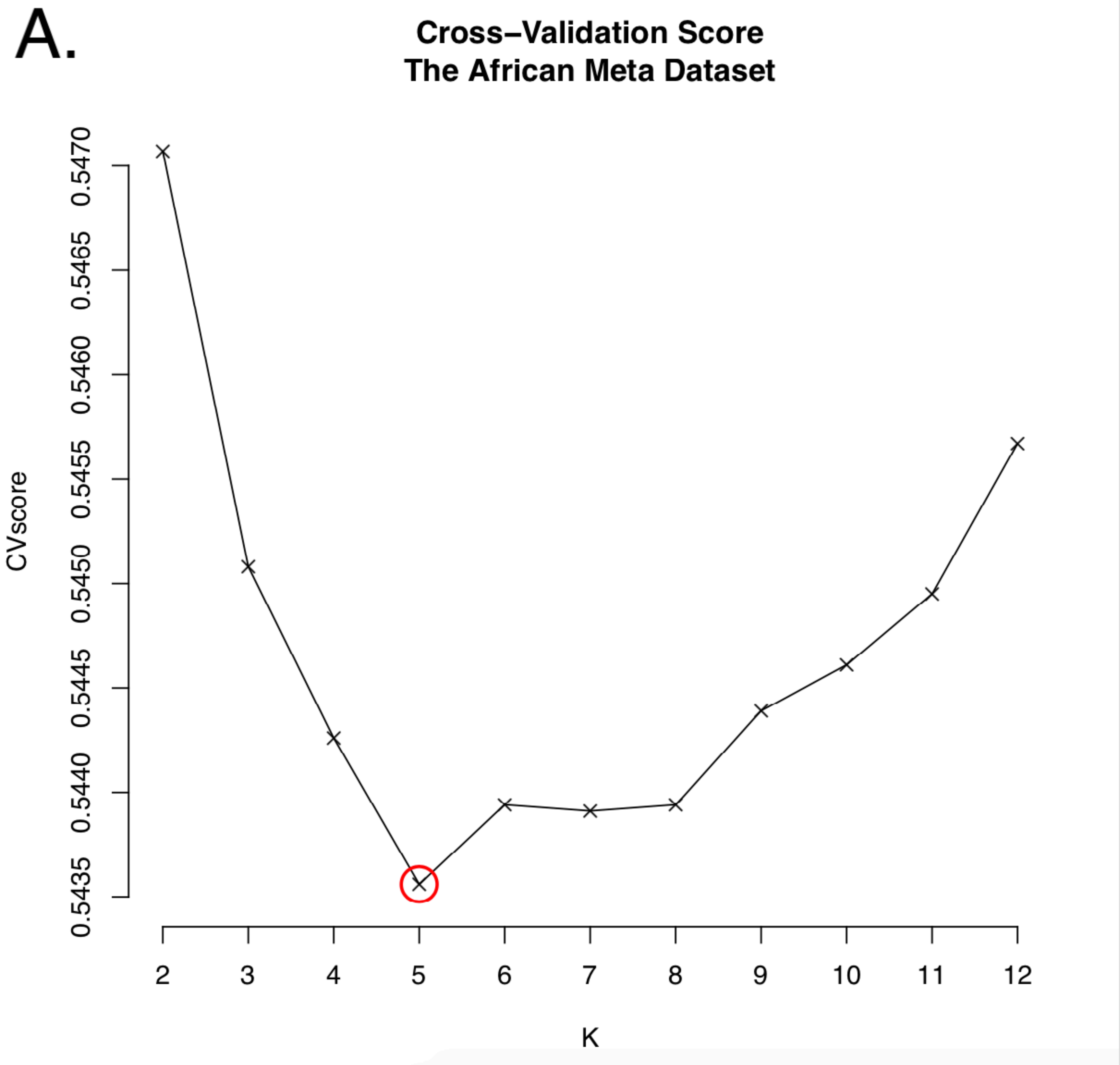
A.
Cross–Validation Score
The African Meta Dataset
CVscore
0.5435
0.5440
0.5445
0.5450
0.5455
0.5460
0.5465
0.5470
2
3
4
5
6
7
8
9
10
11
12
K

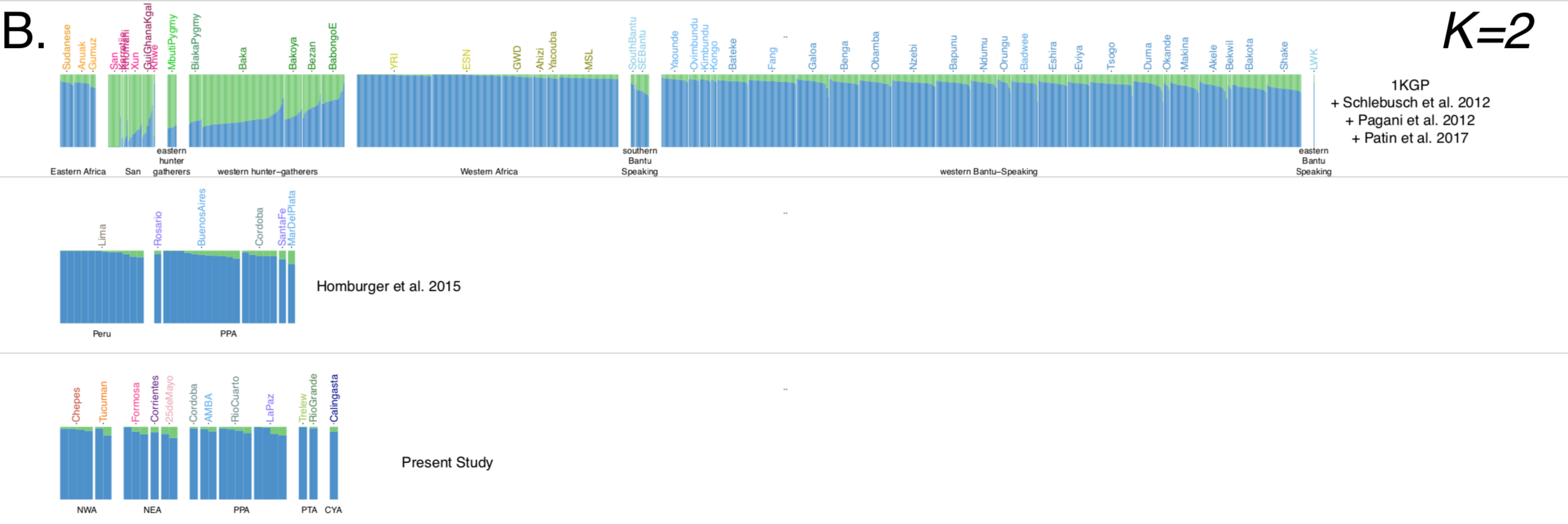

B.
K=2
Sudanese
Anuak
Gumuz
San
Xun
GuiGhanaKgal
Khwe
MbutiPygmy
BiakaPygmy
Baka
Bakoya
Bezan
BabongoE
YRI
ESN
GWD
Ahizi
Yacouba
MSL
SouthBantu
SEBantu
Yaounde
Ovimbundu
Kimbundu
Kongo
Bateke
Fang
Galoa
Benga
Obamba
Nzebi
Bapunu
Ndumu
Orungu
Badwee
Eshira
Eviya
Tsogo
Duma
Okande
Makina
Akele
Bekwil
Bakota
Shake
LWK
Eastern Africa
San
eastern hunter gatherers
western hunter–gatherers
Western Africa
southern Bantu Speaking
western Bantu–Speaking
eastern Bantu Speaking
1KGP
+ Schlebusch et al. 2012
+ Pagani et al. 2012
+ Patin et al. 2017
Lima
Rosario
BuenosAires
Cordoba
SantaFe
MarDelPlata
Peru
PPA
Homburger et al. 2015
Chepes
Tucuman
Formosa
Corrientes
25deMayo
Cordoba
AMBA
RioCuarto
LaPaz
Trelew
RioGrande
Calingasta
NWA
NEA
PPA
PTA
CYA
Present Study

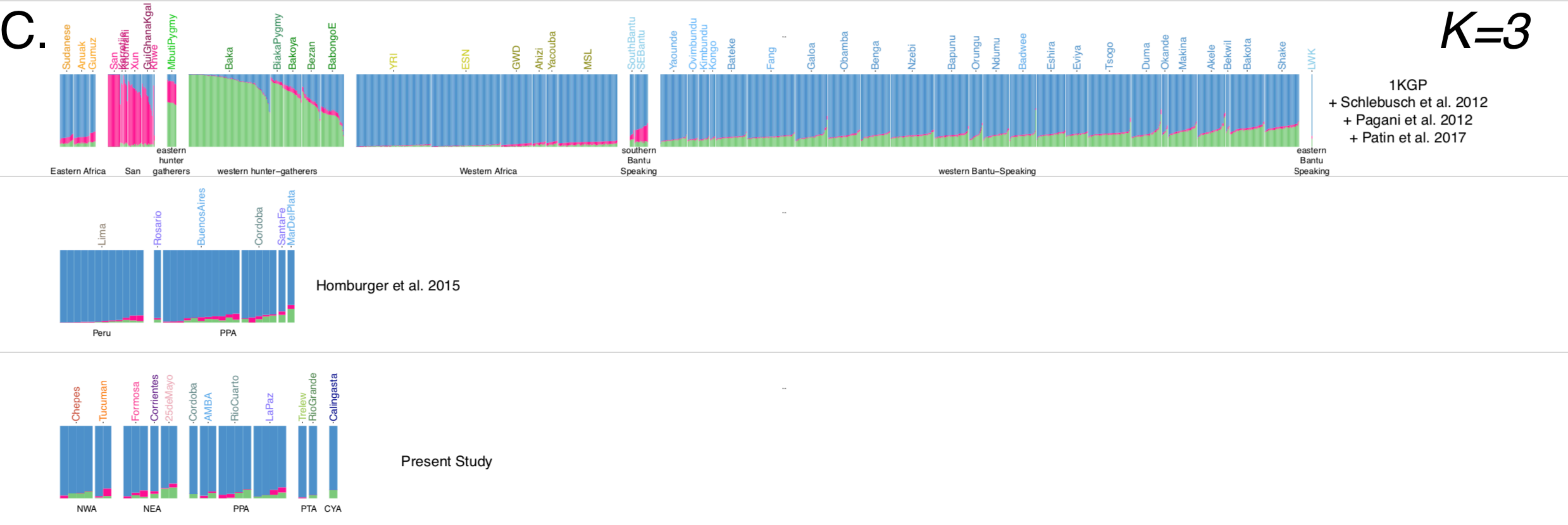
C.
K=3
1KGP
+ Schlebusch et al. 2012
+ Pagani et al. 2012
+ Patin et al. 2017
Eastern Africa
San
eastern hunter gatherers
western hunter–gatherers
Western Africa
southern Bantu Speaking
western Bantu–Speaking
eastern Bantu Speaking
Homburger et al. 2015
Peru
PPA
Present Study
NWA
NEA
PPA
PTA
CYA

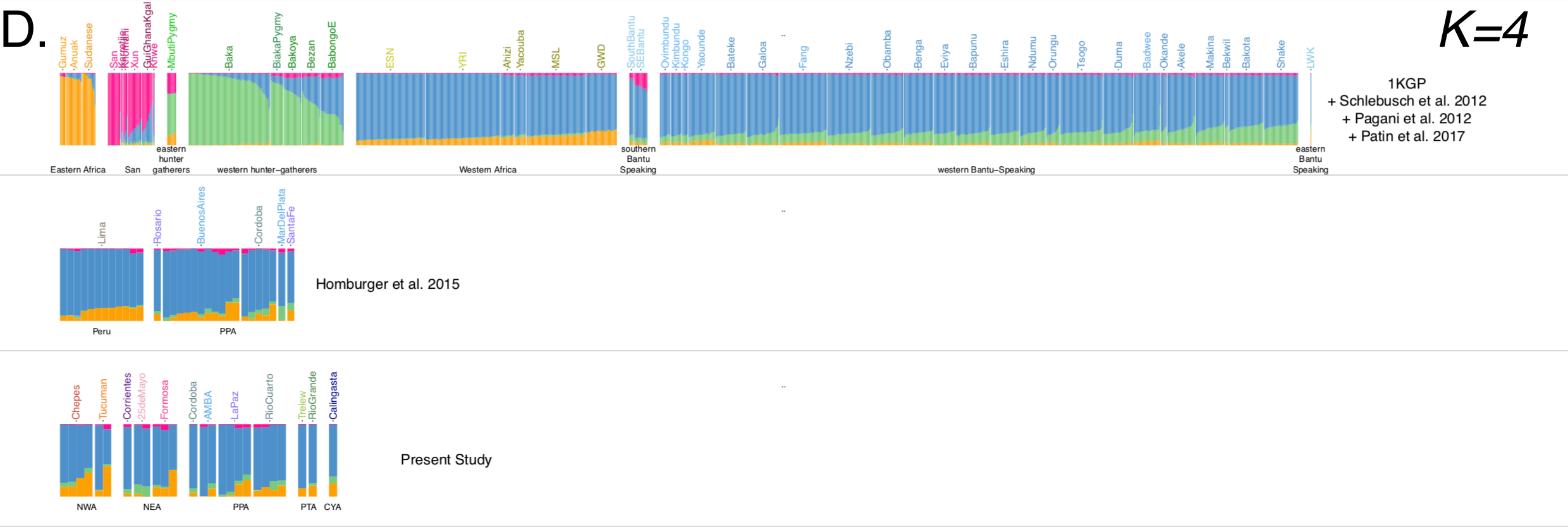

D.
K=4
Gumuz
Anuak
Sudanese
San
Xun
GuiGhanaKgal
Khwe
MbutiPygmy
Baka
BiakaPygmy
Bakoya
Bezan
BabongoE
ESN
YRI
Ahizi
Yacouba
MSL
GWD
SouthBantu
SEBantu
Ovimbundu
Kimbundu
Kongo
Yaounde
Bateke
Galoa
Fang
Nzebi
Obamba
Benga
Eviya
Bapunu
Eshira
Ndumu
Orungu
Tsogo
Duma
Badwee
Okande
Akele
Makina
Bekwil
Bakota
Shake
LWK
1KGP
+ Schlebusch et al. 2012
+ Pagani et al. 2012
+ Patin et al. 2017
Eastern Africa
San
eastern hunter gatherers
western hunter–gatherers
Western Africa
southern Bantu Speaking
western Bantu–Speaking
eastern Bantu Speaking
Lima
Rosario
BuenosAires
Cordoba
MarDelPlata
SantaFe
Homburger et al. 2015
Peru
PPA
Chepes
Tucuman
Corrientes
25deMayo
Formosa
Cordoba
AMBA
LaPaz
RioCuarto
Trelew
RioGrande
Calingasta
Present Study
NWA
NEA
PPA
PTA
CYA
