# Supplementary material for: Fine-scale genomic analyses of admixed individuals reveal unrecognized genetic ancestry components in Argentina
Source: PLoS One. 2020 Jul 16;15(7):e0233808. doi: 10.1371/journal.pone.0233808 (PMC7365470; doi:10.1371/journal.pone.0233808)
Supplement: S13 Fig — (A) Cross-validation scores K from 2 to 10. (B) Admixture for K = 2. (C) Admixture for K = 4. (D) Admixture plots for K = 5. CYA: Cuyo Region; NEA: Northeastern Region, NWA: Northwestern Region; PPA: Pampean Region; PTA: Patagonia Region. (PDF) [file pone.0233808.s013.pdf]

A.

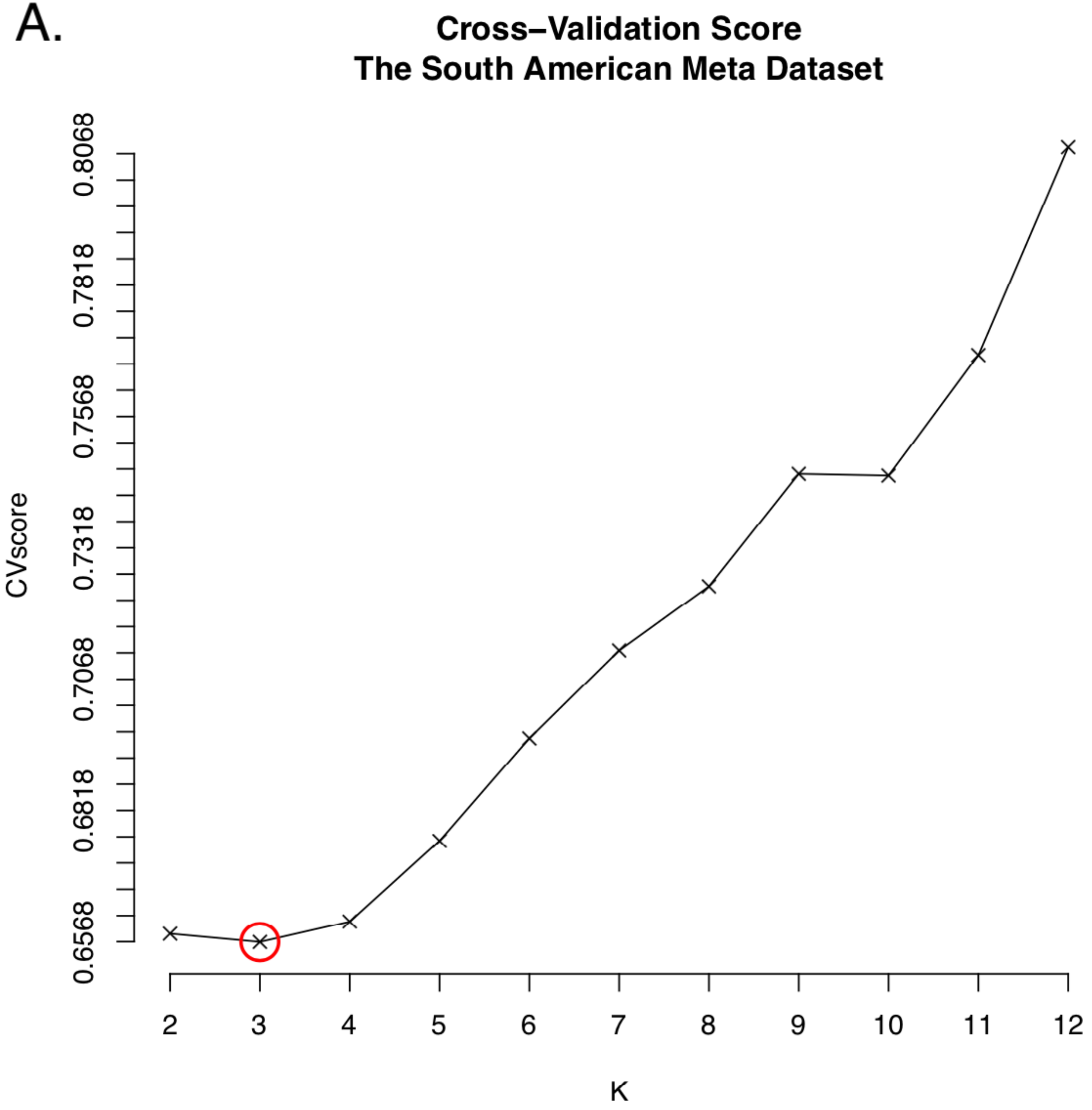

Cross–Validation Score
The South American Meta Dataset
CVscore
0.6568
0.6818
0.7068
0.7318
0.7568
0.7818
0.8068
K
2
3
4
5
6
7
8
9
10
11
12

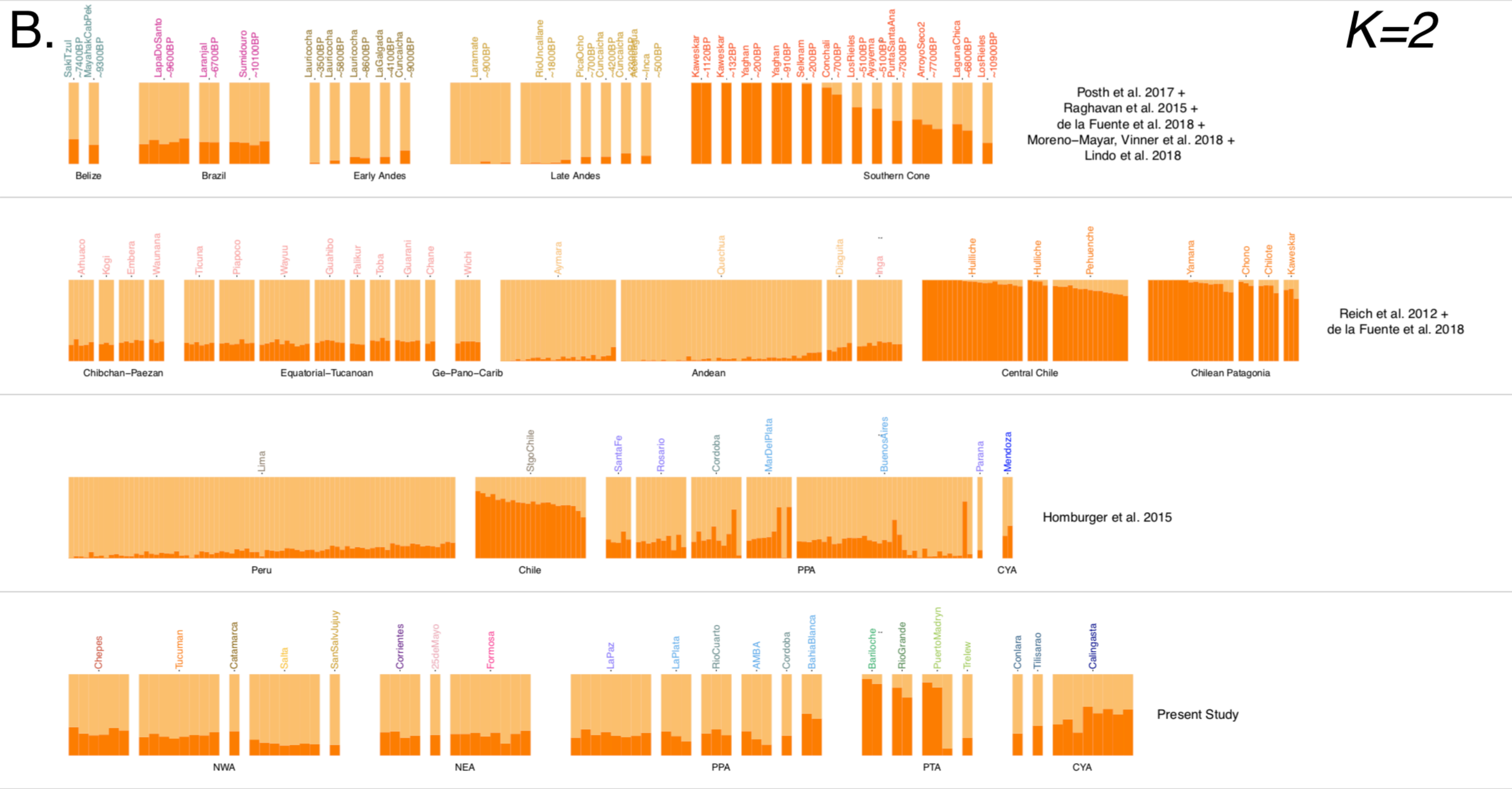
B.
K=2
SakiTzul ~7400BP
MayahakCabPek ~9300BP
LapaDoSanto ~9600BP
Laranjal ~6700BP
Sumidouro ~10100BP
Lauricocha ~3500BP
Lauricocha ~5800BP
Lauricocha ~8600BP
LaGalgada ~4100BP
Cuncaicha ~9000BP
Laramate ~900BP
RioUncallane ~1800BP
PicaOcho ~700BP
Cuncaicha ~4200BP
Cuncaicha ~3300BP
Inca ~500BP
Kaweskar ~1120BP
Kaweskar ~132BP
Yaghan ~200BP
Yaghan ~910BP
Selknam ~200BP
Conchali ~700BP
LosRieles ~5100BP
Ayayema ~5100BP
PuntaSantaAna ~7300BP
ArroyoSeco2 ~7700BP
LagunaChica ~6800BP
LosRieles ~10900BP
Belize
Brazil
Early Andes
Late Andes
Southern Cone
Posth et al. 2017 +
Raghavan et al. 2015 +
de la Fuente et al. 2018 +
Moreno–Mayar, Vinner et al. 2018 +
Lindo et al. 2018
Arhuaco
Kogi
Embera
Waunana
Ticuna
Piapoco
Wayuu
Guahibo
Palikur
Toba
Guarani
Chane
Wichi
Aymara
Quechua
Diaguita
Inga
Huilliche
Hulliche
Pehuenche
Yamana
Chono
Chilote
Kaweskar
Chibchan–Paezan
Equatorial–Tucanoan
Ge–Pano–Carib
Andean
Central Chile
Chilean Patagonia
Reich et al. 2012 +
de la Fuente et al. 2018
Lima
StgoChile
SantaFe
Rosario
Cordoba
MarDelPlata
BuenosAires
Parana
Mendoza
Peru
Chile
PPA
CYA
Homburger et al. 2015
Chepes
Tucuman
Catamarca
Salta
SanSalvJujuy
Corrientes
25deMayo
Formosa
LaPaz
LaPlata
RioCuarto
AMBA
Cordoba
BahiaBlanca
Bariloche
RioGrande
PuertoMadryn
Trelew
Conlara
Tilisarao
Calingasta
NWA
NEA
PPA
PTA
CYA
Present Study

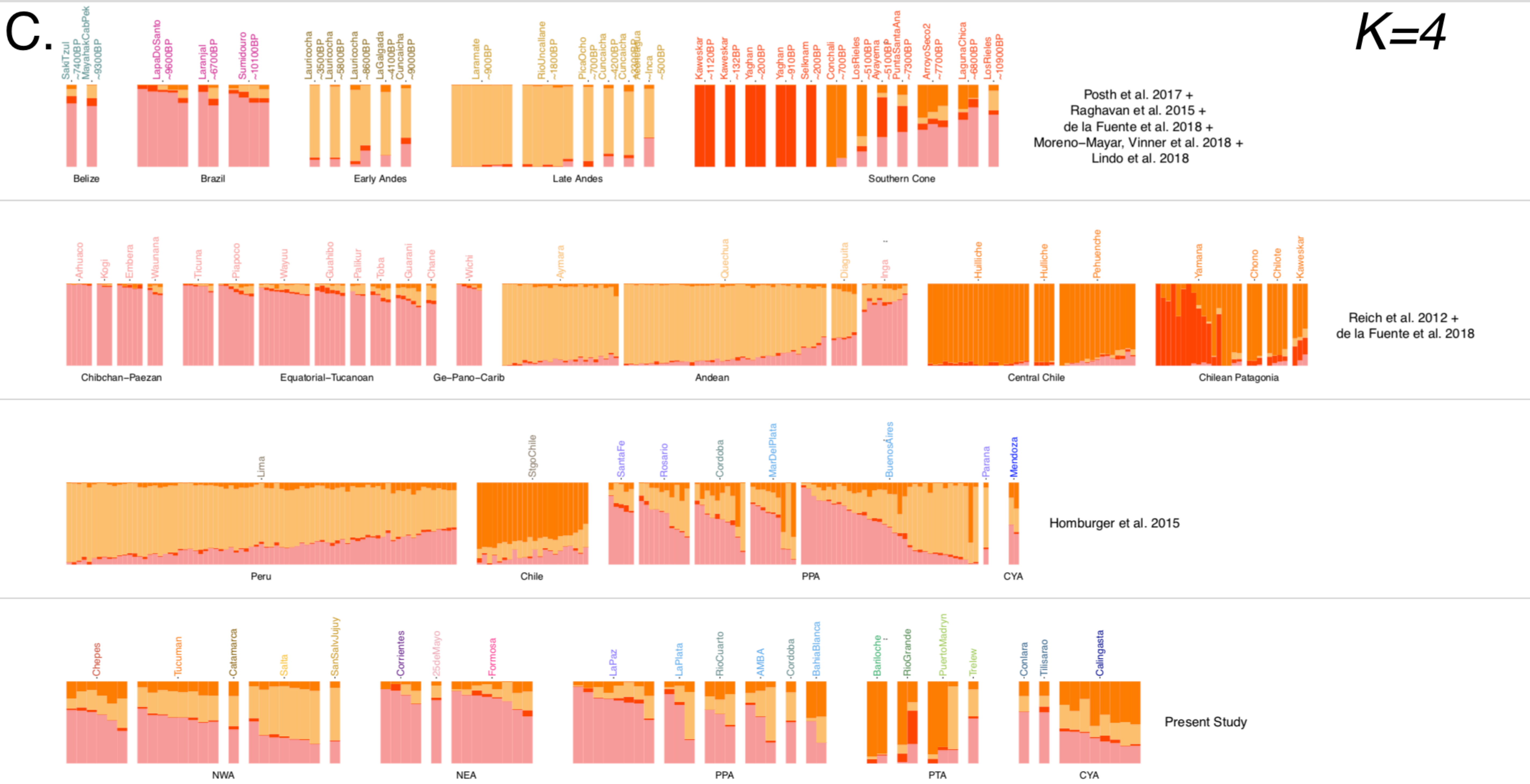

C.
K=4
SakiTzul ~7400BP
MayahakCabPek ~9300BP
LapaDoSanto ~9600BP
Laranjal ~6700BP
Sumidouro ~10100BP
Lauricocha ~3500BP
Lauricocha ~5800BP
Lauricocha ~8600BP
LaGalgada ~4100BP
Cuncaicha ~9000BP
Laramate ~900BP
RioUncallane ~1800BP
PicaOcho ~700BP
Cuncaicha ~4200BP
Inca ~500BP
Kaweskar ~1120BP
Kaweskar ~132BP
Yaghan ~200BP
Yaghan ~910BP
Selknam ~200BP
Conchali ~700BP
LosRieles ~5100BP
Ayayema ~4200BP
PuntaSantaAna ~7300BP
ArroyoSeco2 ~7700BP
LagunaChica ~6800BP
LosRieles ~10900BP
Belize
Brazil
Early Andes
Late Andes
Southern Cone
Posth et al. 2017 +
Raghavan et al. 2015 +
de la Fuente et al. 2018 +
Moreno–Mayar, Vinner et al. 2018 +
Lindo et al. 2018
Arhuaco
Kogi
Embera
Waunana
Ticuna
Piapoco
Wayuu
Guahibo
Palikur
Toba
Guarani
Chane
Wichi
Aymara
Quechua
Diaguita
Inga
Huilliche
Huilliche
Pehuenche
Yamana
Chono
Chilote
Kaweskar
Chibchan–Paezan
Equatorial–Tucanoan
Ge–Pano–Carib
Andean
Central Chile
Chilean Patagonia
Reich et al. 2012 +
de la Fuente et al. 2018
Lima
StgoChile
SantaFe
Rosario
Cordoba
MarDelPlata
BuenosAires
Parana
Mendoza
Peru
Chile
PPA
CYA
Homburger et al. 2015
Chepes
Tucuman
Catamarca
Salta
SanSalvJujuy
Corrientes
25deMayo
Formosa
LaPaz
LaPlata
RioCuarto
AMBA
Cordoba
BahiaBlanca
Bariloche
RioGrande
PuertoMadryn
Trelew
Conlara
Tilisarao
Calingasta
NWA
NEA
PPA
PTA
CYA
Present Study

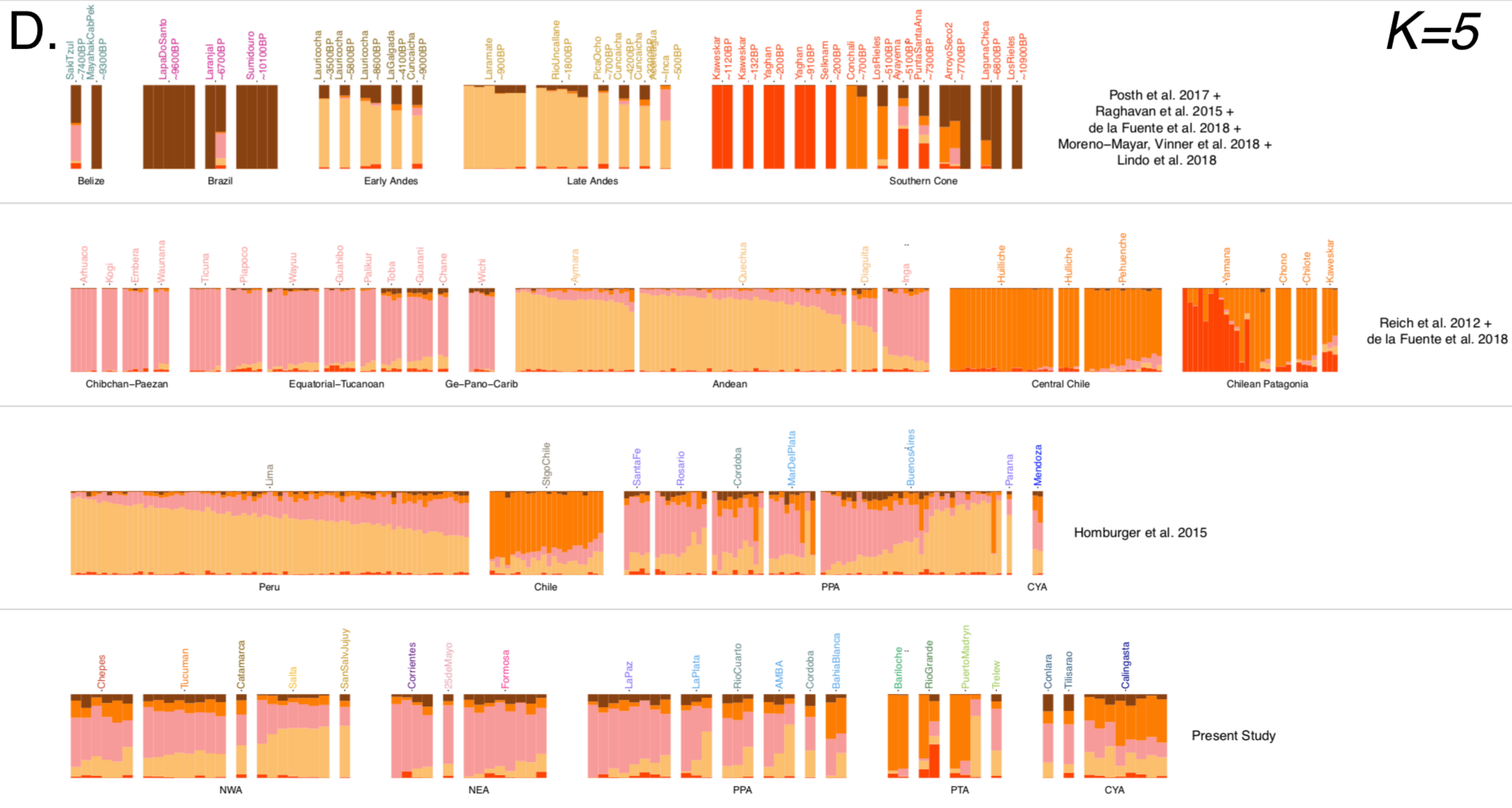
D.
K=5
SakiTzul ~7400BP
MayahakCabPek ~9300BP
LapaDoSanto ~9600BP
Laranjal ~6700BP
Sumidouro ~10100BP
Lauricocha ~3500BP
Lauricocha ~5800BP
Lauricocha ~8600BP
LaGalgada ~4100BP
Cuncaicha ~9000BP
Laramate ~900BP
RioUncallane ~1800BP
PicaOcho ~700BP
Cuncaicha ~4200BP
Inca ~500BP
Kaweskar ~1120BP
Kaweskar ~132BP
Yaghan ~200BP
Yaghan ~910BP
Selknam ~200BP
Conchali ~700BP
LosRieles ~5100BP
PuntaSantaAna ~7300BP
ArroyoSeco2 ~7700BP
LagunaChica ~6800BP
LosRieles ~10900BP
Belize
Brazil
Early Andes
Late Andes
Southern Cone
Posth et al. 2017 +
Raghavan et al. 2015 +
de la Fuente et al. 2018 +
Moreno–Mayar, Vinner et al. 2018 +
Lindo et al. 2018
Arhuaco
Kogi
Embera
Waunana
Ticuna
Piapoco
Wayuu
Guahibo
Palikur
Toba
Guarani
Chane
Wichi
Aymara
Quechua
Diaguita
Inga
Huilliche
Hulliche
Pehuenche
Yamana
Chono
Chilote
Kaweskar
Chibchan–Paezan
Equatorial–Tucanoan
Ge–Pano–Carib
Andean
Central Chile
Chilean Patagonia
Reich et al. 2012 +
de la Fuente et al. 2018
Lima
StgoChile
SantaFe
Rosario
Cordoba
MarDelPlata
BuenosAires
Parana
Mendoza
Peru
Chile
PPA
CYA
Homburger et al. 2015
Chepes
Tucuman
Catamarca
Salta
SanSalvJujuy
Corrientes
25deMayo
Formosa
LaPaz
LaPlata
RioCuarto
AMBA
Cordoba
BahiaBlanca
Bariloche
RioGrande
PuertoMadryn
Trelew
Conlara
Tilisarao
Calingasta
NWA
NEA
PPA
PTA
CYA
Present Study
