# Supplementary material for: Fine-scale genomic analyses of admixed individuals reveal unrecognized genetic ancestry components in Argentina
Source: PLoS One. 2020 Jul 16;15(7):e0233808. doi: 10.1371/journal.pone.0233808 (PMC7365470; doi:10.1371/journal.pone.0233808)
Supplement: S14 Fig — Unmasked and masked data refers to DS1 and DS6, respectively. Spearman correlation coefficients and associated P-values are shown. CAN: Central Andes; STF: Subtropical and Tropical Forests; CCP: Central Chile / Patagonia. (PDF) [file pone.0233808.s014.pdf]

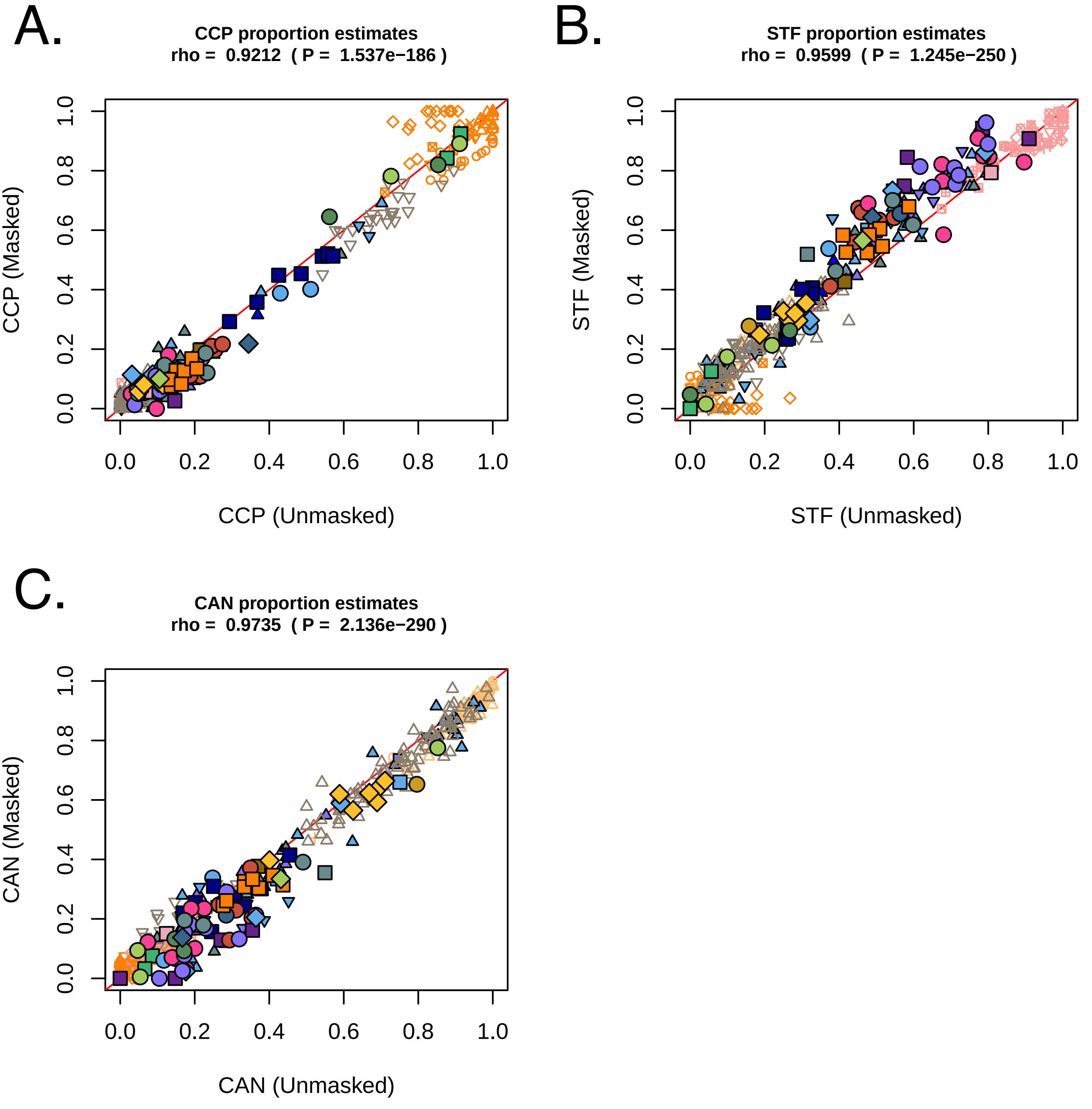
A.
CCP proportion estimates
rho = 0.9212 (P = 1.537e−186)
CCP (Masked)
CCP (Unmasked)
B.
STF proportion estimates
rho = 0.9599 (P = 1.245e−250)
STF (Masked)
STF (Unmasked)
C.
CAN proportion estimates
rho = 0.9735 (P = 2.136e−290)
CAN (Masked)
CAN (Unmasked)
0.0
0.2
0.4
0.6
0.8
1.0
