# Supplementary material for: Fine-scale genomic analyses of admixed individuals reveal unrecognized genetic ancestry components in Argentina
Source: PLoS One. 2020 Jul 16;15(7):e0233808. doi: 10.1371/journal.pone.0233808 (PMC7365470; doi:10.1371/journal.pone.0233808)
Supplement: S23 Fig — Admixed individuals from Santiago de Chile were removed to perform the analyses presented in this figure. (A) f3(Target; S1, S2) to test for treeness; (B) f4(YRI, Target; S1, S2) to test whether Target shares more ancestry with S1 or S2; (C) f3(YRI; CWA; Ancient); (D) f4(YRI, CWA; Ancient Beringia, Ancient); (E) f4(Ancient, CCP; CWA, YRI); (F) f4(Ancient, CWA; CCP YRI); (G) f4(YRI, Ancient; CWA, CCP). CCP: Central Chile / Patagonia. (PDF) [file pone.0233808.s023.pdf]

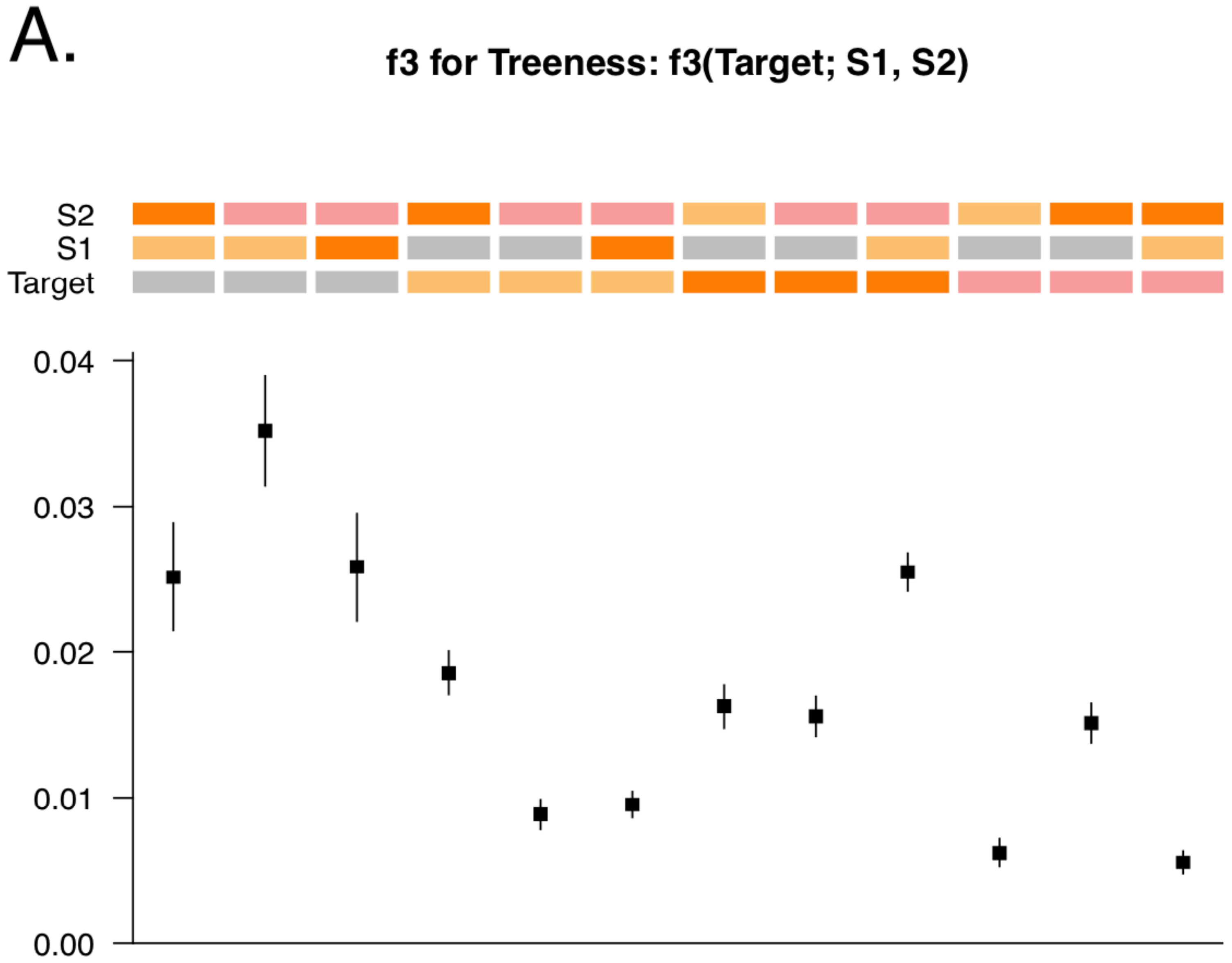
A.
f3 for Treeness: f3(Target; S1, S2)
S2
S1
Target
0.04
0.03
0.02
0.01
0.00

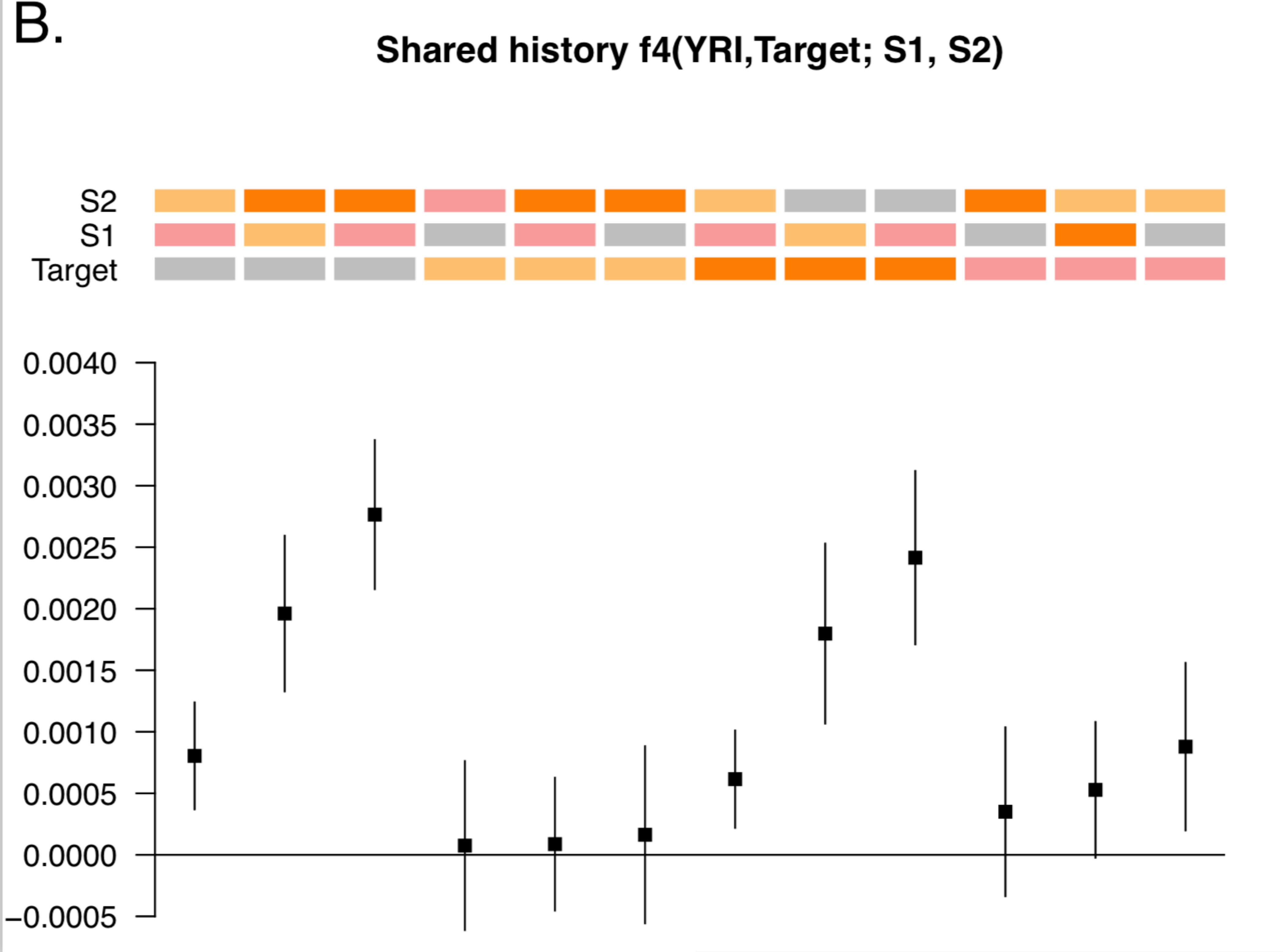

B.
Shared history f4(YRI,Target; S1, S2)
S2
S1
Target
0.0040
0.0035
0.0030
0.0025
0.0020
0.0015
0.0010
0.0005
0.0000
−0.0005

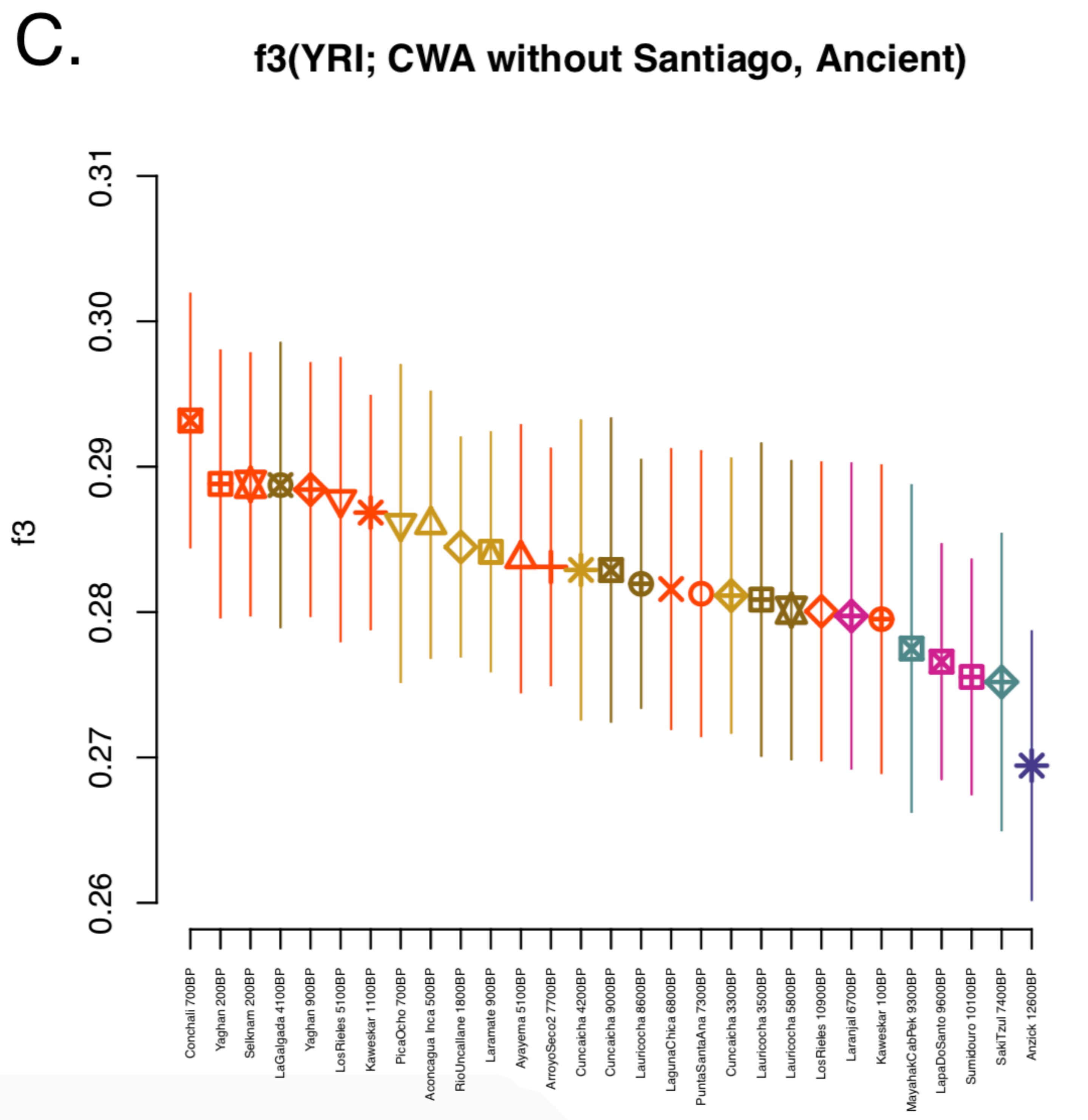
C.
f3(YRI; CWA without Santiago, Ancient)
f3
0.26
0.27
0.28
0.29
0.30
0.31
Conchali 700BP
Yaghan 200BP
Selknam 200BP
LaGalgada 4100BP
Yaghan 900BP
LosRieles 5100BP
Kaweskar 1100BP
PicaOcho 700BP
Aconcagua Inca 500BP
RioUncallane 1800BP
Laramate 900BP
Ayayema 5100BP
ArroyoSeco2 7700BP
Cuncaicha 4200BP
Cuncaicha 9000BP
Lauricocha 8600BP
LagunaChica 6800BP
PuntaSantaAna 7300BP
Cuncaicha 3300BP
Lauricocha 3500BP
Lauricocha 5800BP
LosRieles 10900BP
Laranjal 6700BP
Kaweskar 100BP
MayahakCabPek 9300BP
LapaDoSanto 9600BP
Sumidouro 10100BP
SakiTzul 7400BP
Anzick 12600BP

D.

f4(YRI, CWA without Santiago; Ancient Beringia, Ancient)

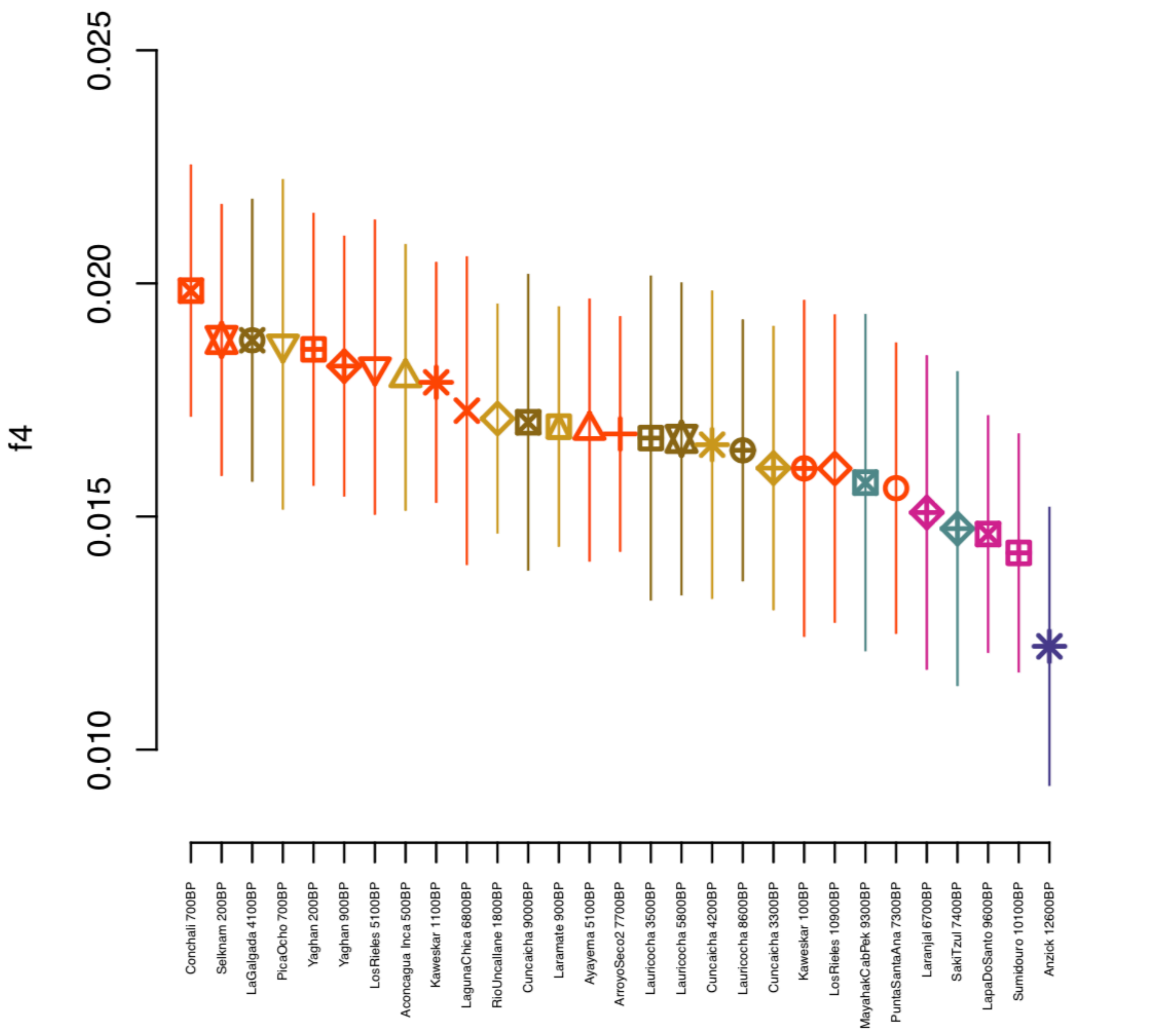

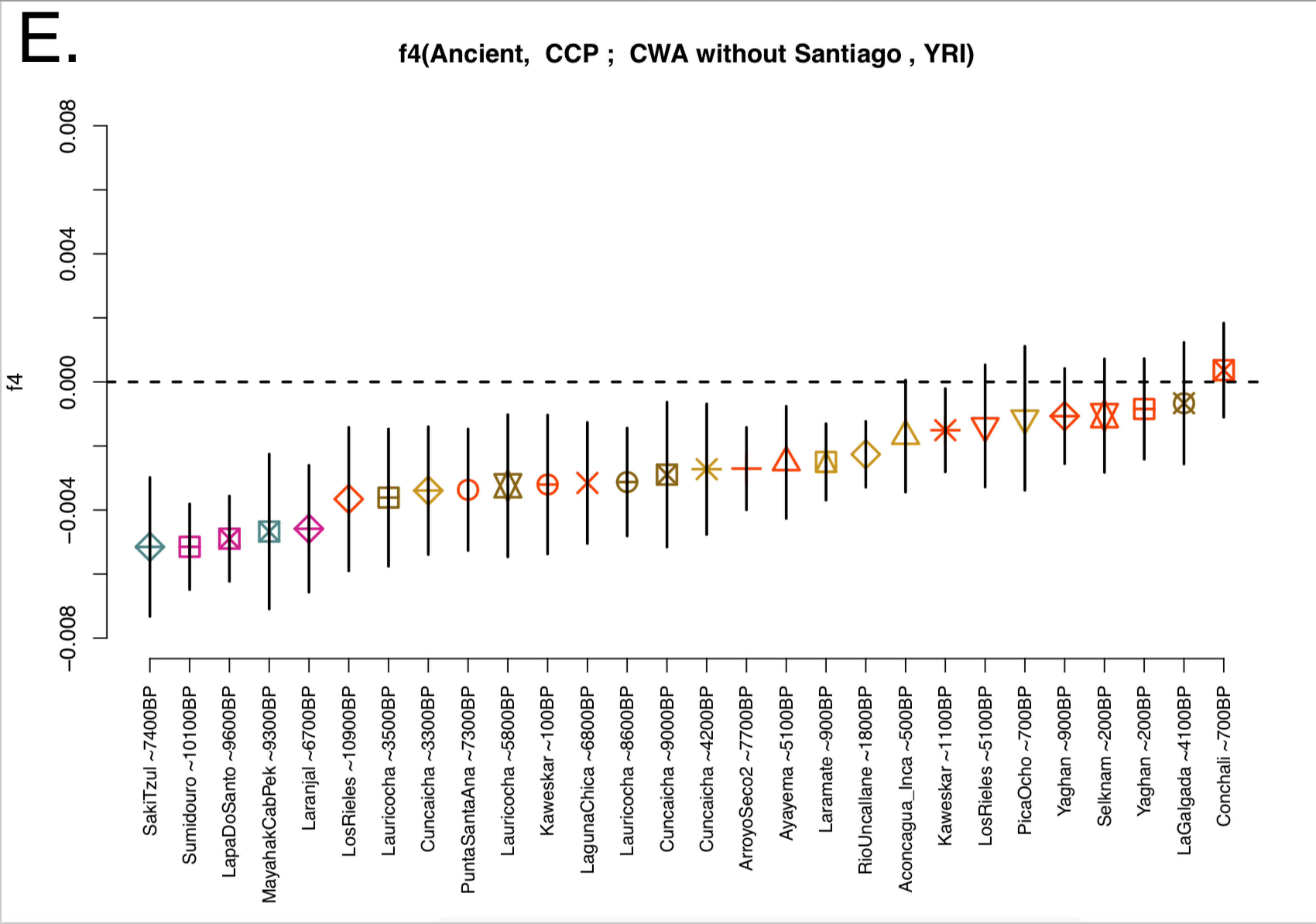
E.
f4(Ancient, CCP ; CWA without Santiago , YRI)
f4
0.008
0.004
0.000
-0.004
-0.008
SakiTzul ~7400BP
Sumidouro ~10100BP
LapaDoSanto ~9600BP
MayahakCabPek ~9300BP
Laranjal ~6700BP
LosRieles ~10900BP
Lauricocha ~3500BP
Cuncaicha ~3300BP
PuntaSantaAna ~7300BP
Lauricocha ~5800BP
Kaweskar ~100BP
LagunaChica ~6800BP
Lauricocha ~8600BP
Cuncaicha ~9000BP
Cuncaicha ~4200BP
ArroyoSeco2 ~7700BP
Ayayema ~5100BP
Laramate ~900BP
RioUncallane ~1800BP
Aconcagua_Inca ~500BP
Kaweskar ~1100BP
LosRieles ~5100BP
PicaOcho ~700BP
Yaghan ~900BP
Selknam ~200BP
Yaghan ~200BP
LaGalgada ~4100BP
Conchali ~700BP

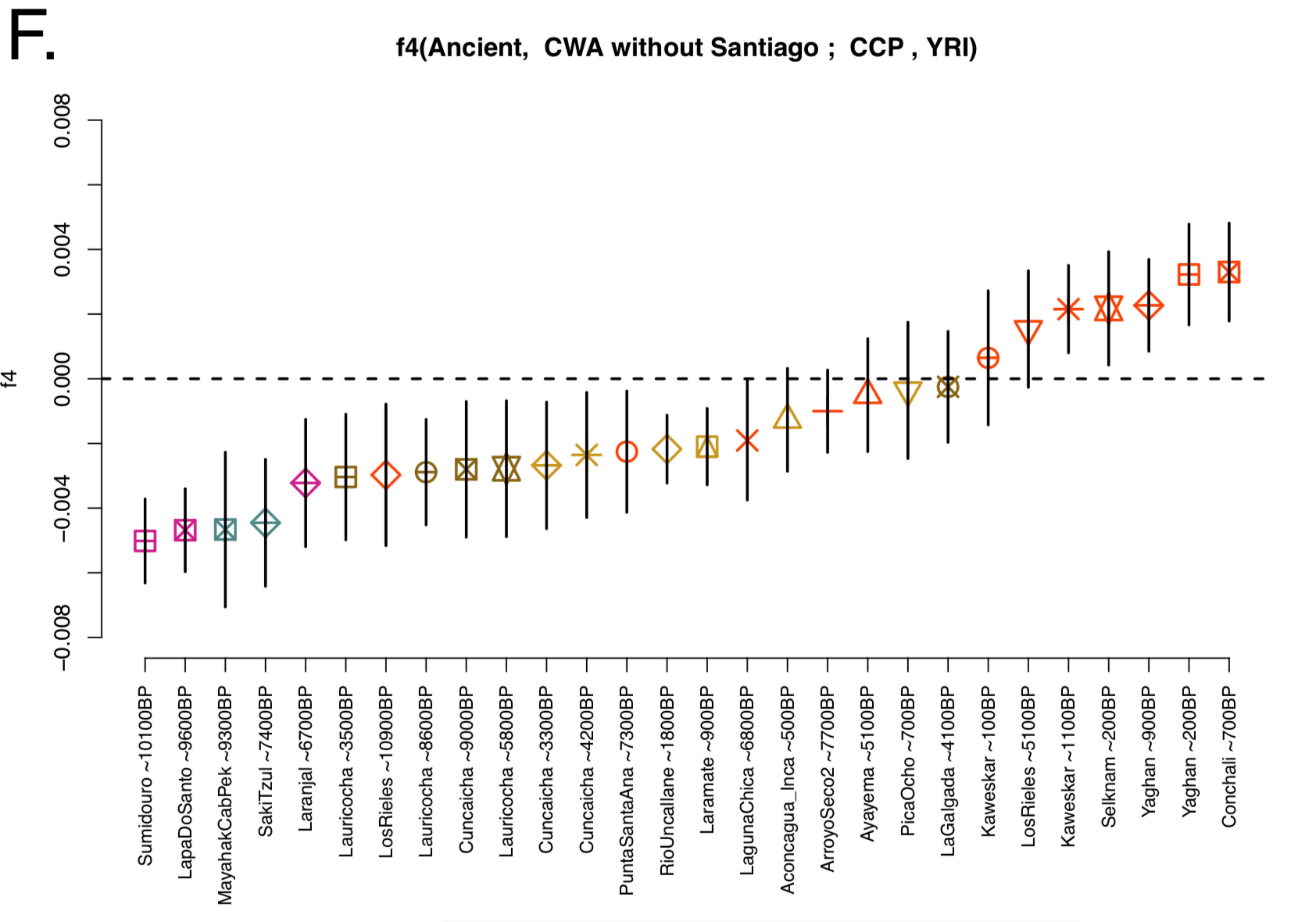

F.
f4(Ancient, CWA without Santiago ; CCP , YRI)
f4
0.008
0.004
0.000
-0.004
-0.008
Sumidouro ~10100BP
LapaDoSanto ~9600BP
MayahakCabPek ~9300BP
SakiTzul ~7400BP
Laranjal ~6700BP
Lauricocha ~3500BP
LosRieles ~10900BP
Lauricocha ~8600BP
Cuncaicha ~9000BP
Lauricocha ~5800BP
Cuncaicha ~3300BP
Cuncaicha ~4200BP
PuntaSantaAna ~7300BP
RioUncallane ~1800BP
Laramate ~900BP
LagunaChica ~6800BP
Aconcagua_Inca ~500BP
ArroyoSeco2 ~7700BP
Ayayema ~5100BP
PicaOcho ~700BP
LaGalgada ~4100BP
Kaweskar ~100BP
LosRieles ~5100BP
Kaweskar ~1100BP
Selknam ~200BP
Yaghan ~900BP
Yaghan ~200BP
Conchali ~700BP

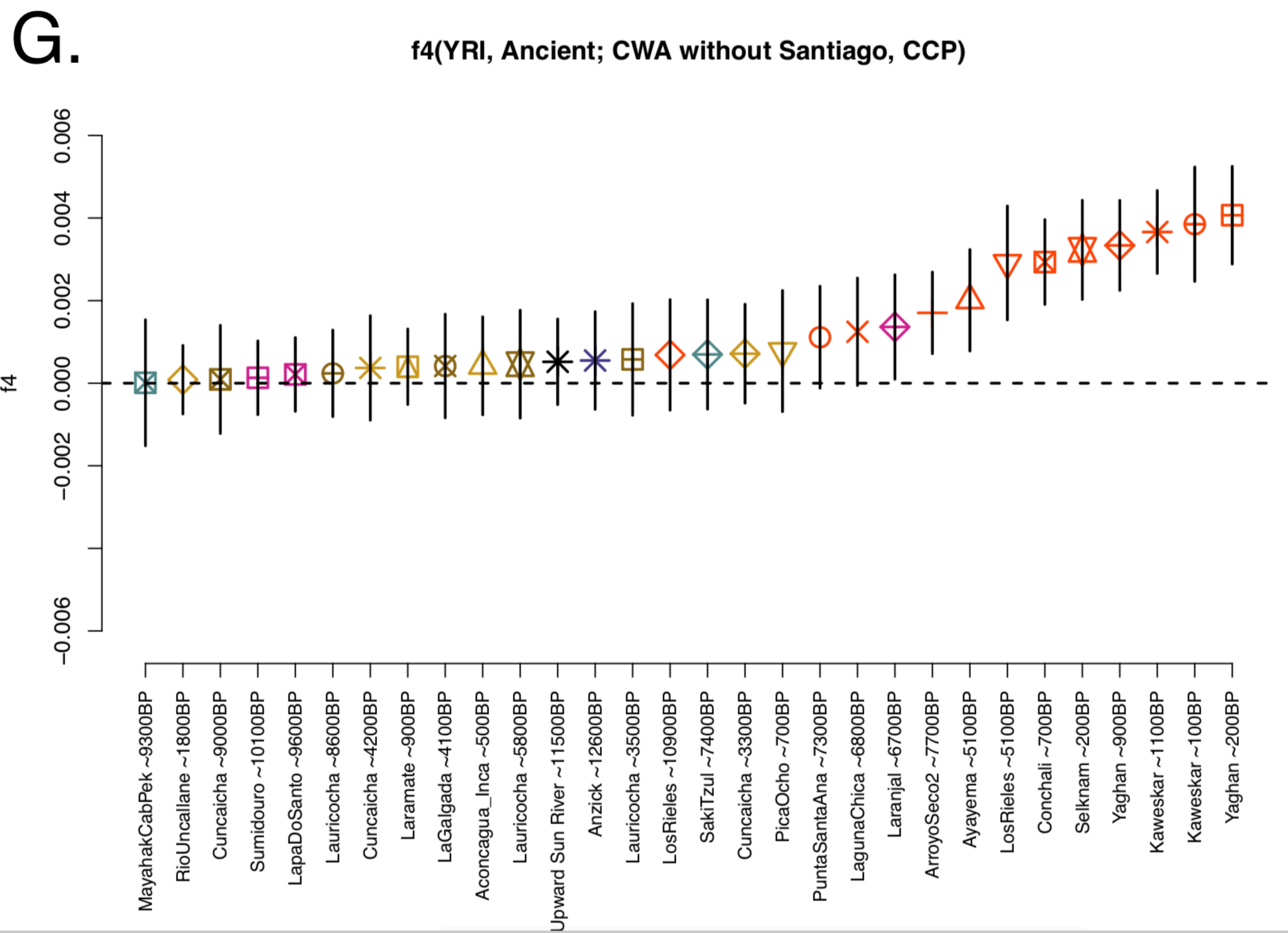
G.
f4(YRI, Ancient; CWA without Santiago, CCP)
f4
0.006
0.004
0.002
0.000
−0.002
−0.006
MayahakCabPek ~9300BP
RioUncallane ~1800BP
Cuncaicha ~9000BP
Sumidouro ~10100BP
LapaDoSanto ~9600BP
Lauricocha ~8600BP
Cuncaicha ~4200BP
Laramate ~900BP
LaGalgada ~4100BP
Aconcagua_Inca ~500BP
Lauricocha ~5800BP
Upward Sun River ~11500BP
Anzick ~12600BP
Lauricocha ~3500BP
LosRieles ~10900BP
SakiTzul ~7400BP
Cuncaicha ~3300BP
PicaOcho ~700BP
PuntaSantaAna ~7300BP
LagunaChica ~6800BP
Laranjal ~6700BP
ArroyoSeco2 ~7700BP
Ayayema ~5100BP
LosRieles ~5100BP
Conchali ~700BP
Selknam ~200BP
Yaghan ~900BP
Kaweskar ~1100BP
Kaweskar ~100BP
Yaghan ~200BP
