# Supplementary material for: Fine-scale genomic analyses of admixed individuals reveal unrecognized genetic ancestry components in Argentina
Source: PLoS One. 2020 Jul 16;15(7):e0233808. doi: 10.1371/journal.pone.0233808 (PMC7365470; doi:10.1371/journal.pone.0233808)
Supplement: S2 Table — (PDF) [file pone.0233808.s030.pdf]

	DS1	DS2	DS2p	DS3	DS3p	DS4	DS5	DS6	DS7
**Analyses**	Worlwide Context	Phasing	Local Ancestry	Phasing	Local Ancestry	European Ancestry	African Ancestry	Native American Ancestry	*F-stats*
Present Study	✔	✔	✔			✔(a)	✔(a)	✔(a)	✔(a)
(Homburger et al. 2015)	✔			✔	✔	✔(a)	✔(a)	✔(a)	✔(a)
(de la Fuente et al. 2018)	✔	✔	✔					✔(a)	✔(a)
(Reich et al. 2012)	✔							✔	✔(b)
(The 1000 Genomes Project Consortium 2015)	✔(c)	✔(d)	✔(d)	✔(d)	✔(d)	✔(e)	✔(f)		✔(g)
(Nelson et al. 2008)(h)						✔			
(Pagani et al. 2012)							✔(i)		
(Schlebusch et al. 2012)							✔(i)		
(Patin et al. 2017) and references therein							✔(i)		
(Raghavan et al. 2015)								✔	✔
(Moreno-mayar et al. 2017)								✔	✔
(Moreno-mayar et al. 2018)								✔	✔
(Lindo et al. 2018)								✔	✔
(Posth et al. 2018)								✔	✔
# SNPs without LD-pruning	64,531	608,501	608,501	694,626	694,626	29,347	137,136	47,003	88,564
# SNPs with LD-pruning	59,237	–	–	–	–	27,634	128,086	39,423	–
# Individuals	2,076	1,645	1,645	1,772	1,772	1,612	1,742	505	632

a. Masked data for the specific ancestry analyzed
b. Including individuals from Mixe population in Mexico
c. Individuals from South America, Africa, Europe, and Afro-Americans
d. Individuals from America, Africa and Europe
e. Individuals from Africa
f. Individuals from South America
g. Only individuals from Yoruba population
h. Only individuals identified in Novembre et al. 2008
i. Removing individuals with less than 99% of African ancestry estimated through Admixture analyses (K=7)
